# Supplementary material for: Investigating the molecular basis for heterophylly in the aquatic plant Potamogeton octandrus (Potamogetonaceae) with comparative transcriptomics
Source: PeerJ. 2018 Feb 28;6:e4448. doi: 10.7717/peerj.4448 (PMC5834931; doi:10.7717/peerj.4448)
Supplement: Supplemental Information 1 [file peerj-06-4448-s001.zip › Supplemental files/Additional file 1.pdf]

Additional file 1. Distribution map of the randomness test of sequence reads in *P. octandrus*..

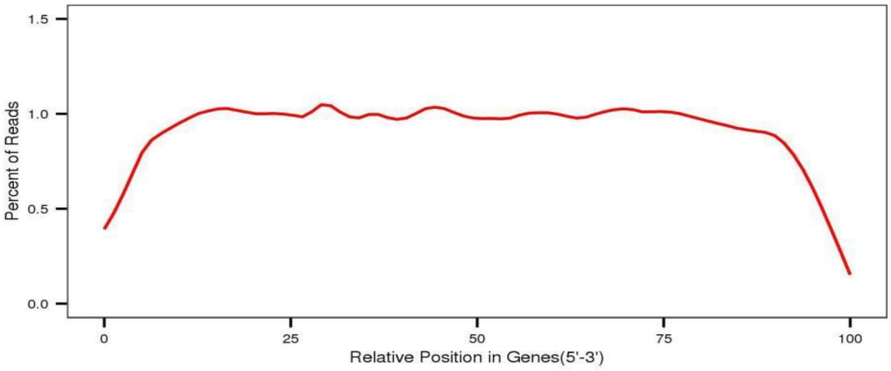

T1

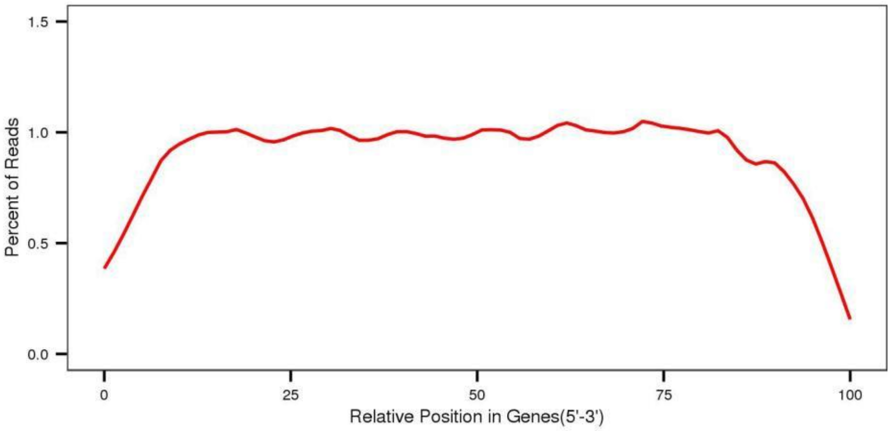

T2

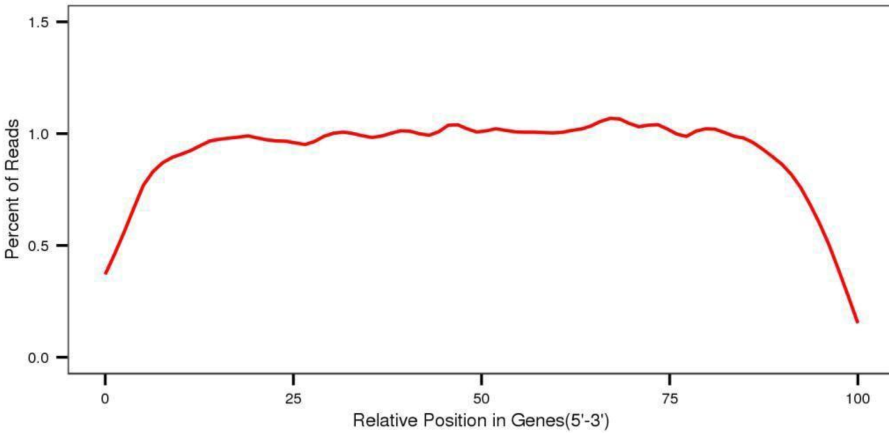

T3

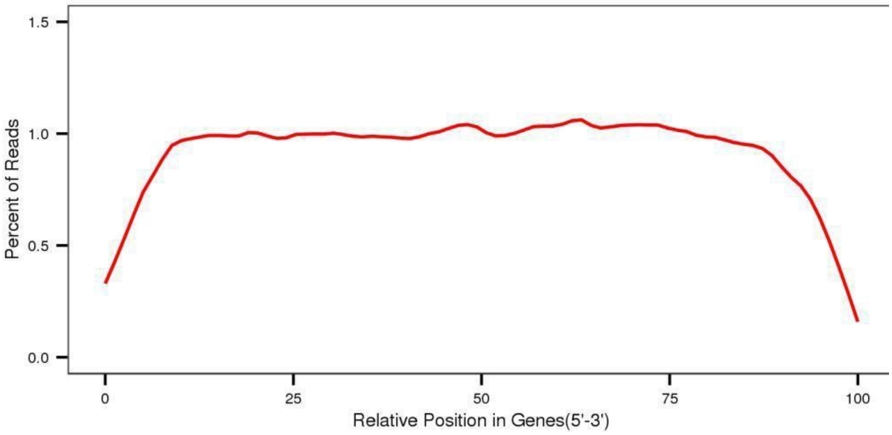
1.5
1.0
0.5
0.0
Percent of Reads
0
25
50
75
100
Relative Position in Genes(5'-3')

T4

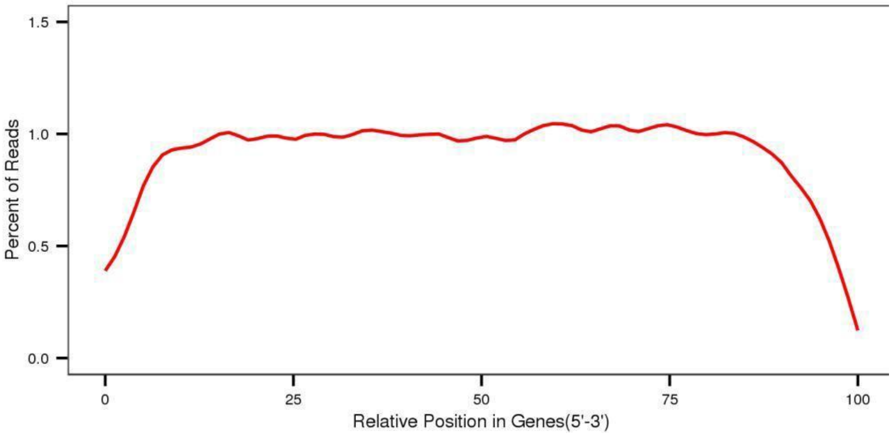
1.5
1.0
0.5
0.0
Percent of Reads
0
25
50
75
100
Relative Position in Genes(5'-3')

T5

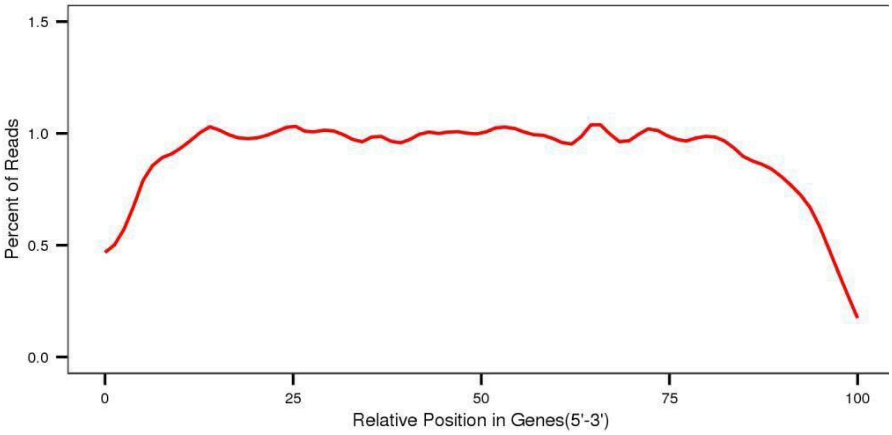
1.5
1.0
0.5
0.0
Percent of Reads
0
25
50
75
100
Relative Position in Genes(5'-3')

T6

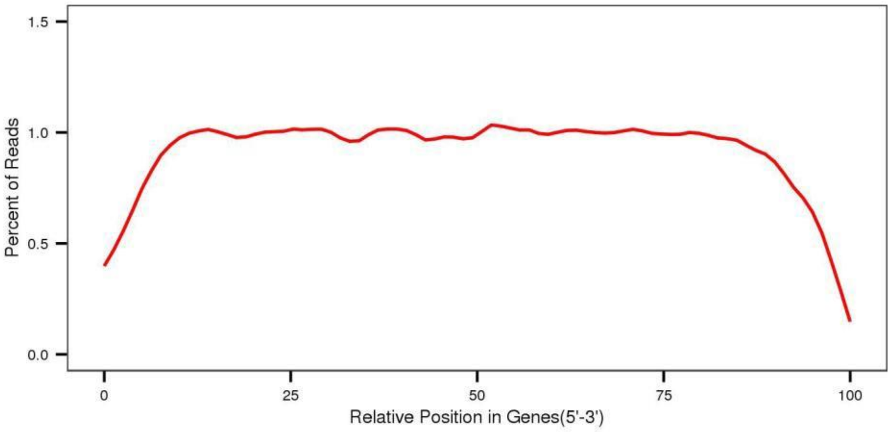
1.5
1.0
0.5
0.0
Percent of Reads
0
25
50
75
100
Relative Position in Genes(5'-3')

T7

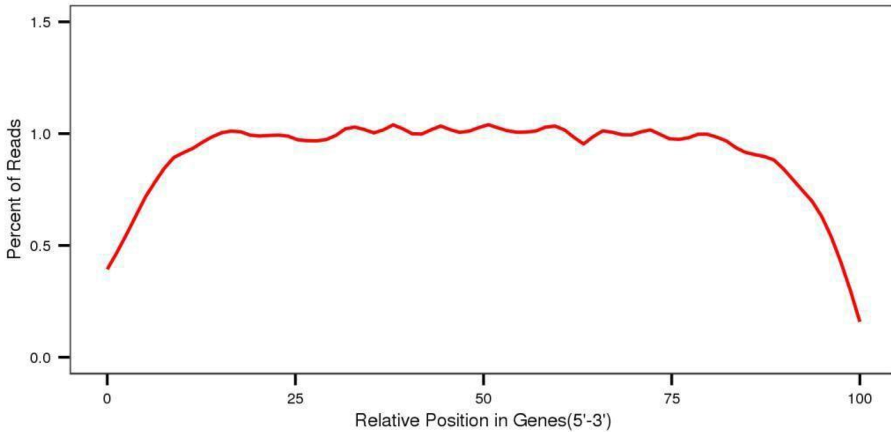
1.5
1.0
0.5
0.0
Percent of Reads
0
25
50
75
100
Relative Position in Genes(5'-3')

T8

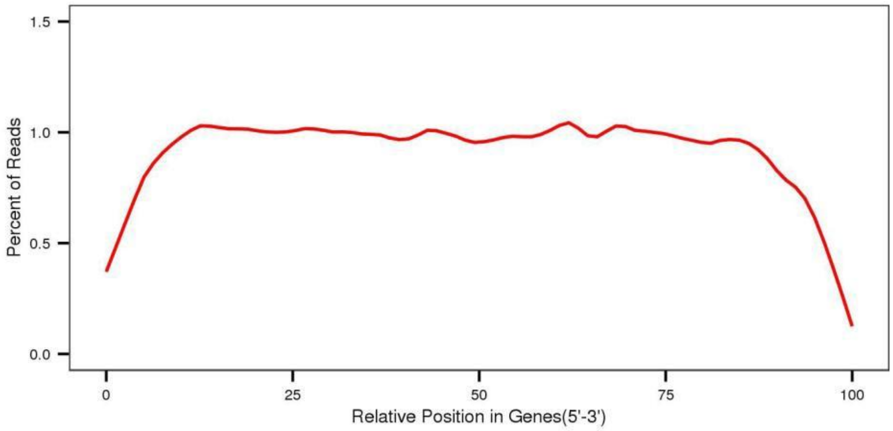
1.5
1.0
0.5
0.0
Percent of Reads
0
25
50
75
100
Relative Position in Genes(5'-3')

T9

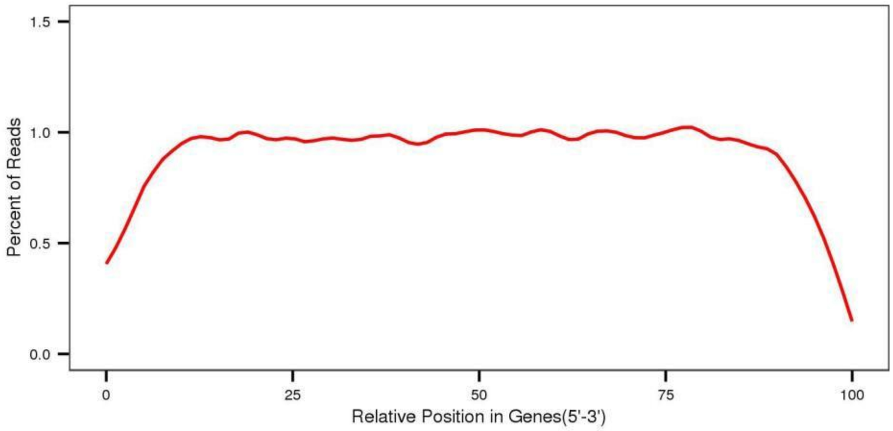
1.5
1.0
0.5
0.0
Percent of Reads
0
25
50
75
100
Relative Position in Genes(5'-3')

T10

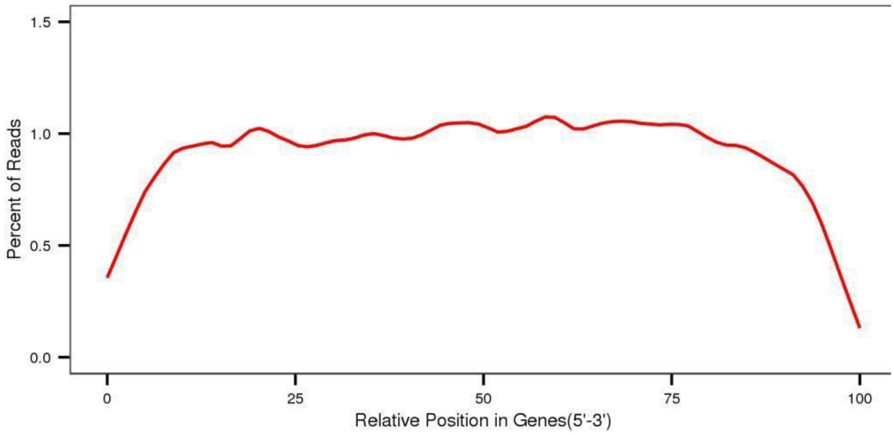
1.5
1.0
0.5
0.0
Percent of Reads
0
25
50
75
100
Relative Position in Genes(5'-3')

T11

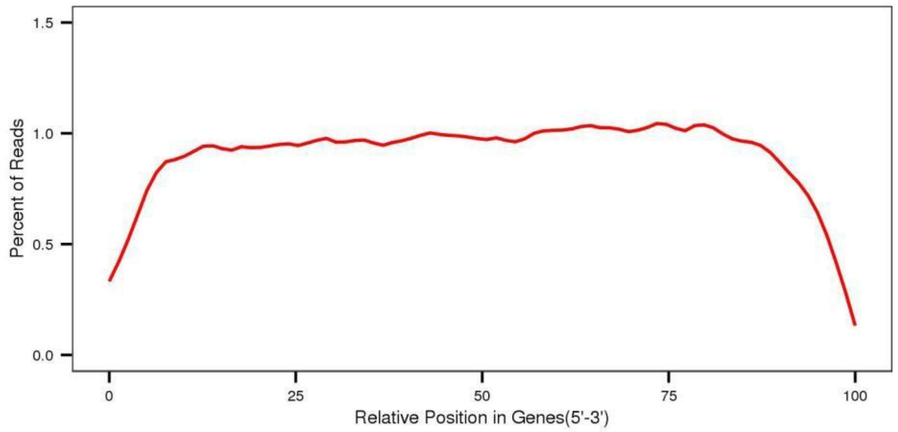

T12

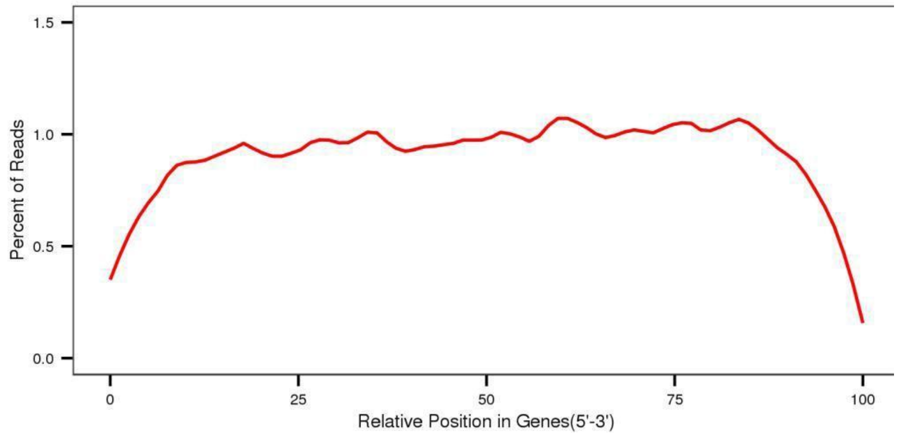

T13

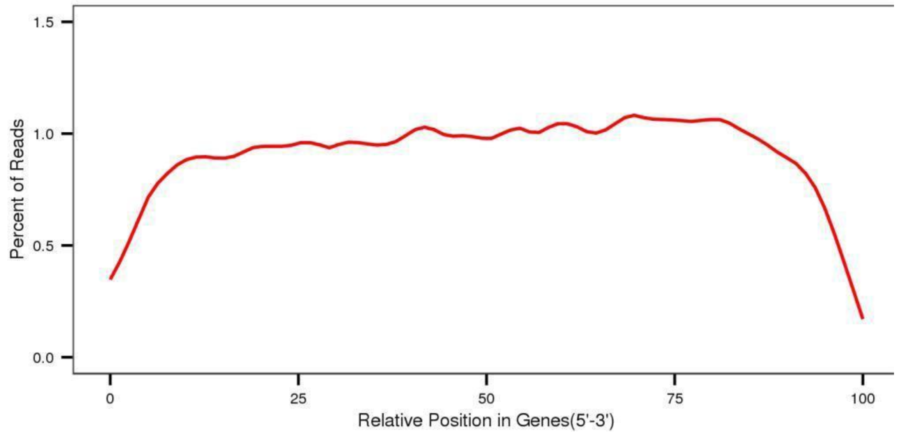

T14

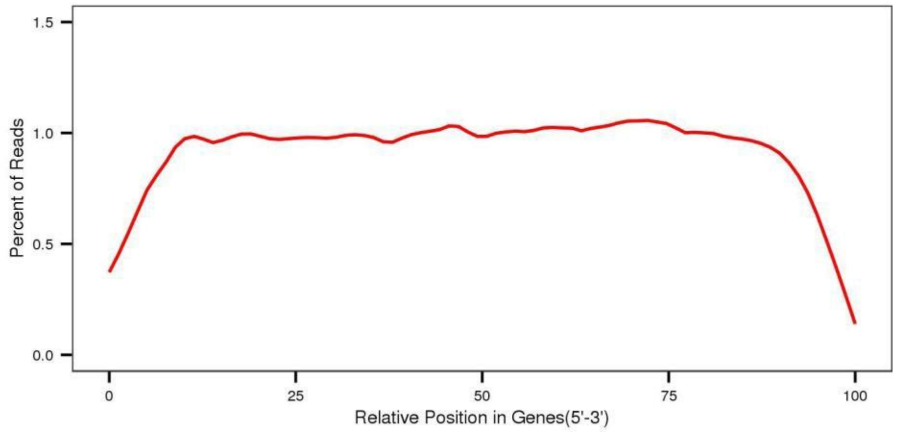

T15
