# Supplementary material for: Investigating the molecular basis for heterophylly in the aquatic plant Potamogeton octandrus (Potamogetonaceae) with comparative transcriptomics
Source: PeerJ. 2018 Feb 28;6:e4448. doi: 10.7717/peerj.4448 (PMC5834931; doi:10.7717/peerj.4448)
Supplement: Supplemental Information 1 [file peerj-06-4448-s001.zip › Supplemental files/Additional file 10.doc]

**Additional file 10.** Representative functions of TF genes showing expression gradients between floating and submerged leaf tissue samples.

TF Family Gene function in model species Description High in G1

MYB cell differentiation Atmyb91

epidermal cell patterning Atmyb0, Atmybb66

ARF auxin signaling ARF5, ARF8 leaf mesophyll ARF16

light signaling ARF8

B3 ABA signaling, GA signaling ABI3, FUS3, LEC2

HB-other

Initiation and maintenance of the shoot apical meristem

KNAT1

Maintenance of the shoot apical meristem

BEL1

GRF leaf morphogenesis AtGRF1, AtGRF2 leaf petiole development AtGRF1, AtGRF2

cell proliferation GIF1/AN3

TALE shoot system development

BP, PNY, KNAT6, ATH1

leaf proximal/distal pattern AS1, AS2

High in G2

AP2 leaf epidermal cell identity Glossy15

C3H photomorphogenesis PEI1

WRKY leaf senescence AtWRKY6 plant growth AtWRKY18

metabolic pathway SUSIBA2

ERF ethylene signaling ERF1

leaf petiole development LEAF PETIOLE

NAC cell division NAP environmental stimuli NTL

auxin signaling AtNAC2

HD-Zip organ and vascular development ATHB8 stomatal development ATHB6

RAV leaf development RAV1 brassinosteroid response RAV1

High in G3

bHLH light signaling PIF4

hormone signaling

FAMA, MUTE, SPCH1/2

stomatal development SPCH, MUTE, FAMA

CO-like Photopreiod response COL3

FAR1 response to red/ far red light FHY3, FAR1

ERF waxy cuticle SHN
