# Supplementary material for: Investigating the molecular basis for heterophylly in the aquatic plant Potamogeton octandrus (Potamogetonaceae) with comparative transcriptomics
Source: PeerJ. 2018 Feb 28;6:e4448. doi: 10.7717/peerj.4448 (PMC5834931; doi:10.7717/peerj.4448)
Supplement: Supplemental Information 1 [file peerj-06-4448-s001.zip › Supplemental files/Additional file 11.doc]

Additional file 11. A list of 70 DEGs involved in the plant hormone signal transduction pathway during heterophyllous leaf development. We defined genes with expression change of |log2FC| ≥ 1 and FDR < 0.01 as DEGs. The values of log2FC and expression patterns of DEGs are presented in the table.
ABA	
	log2FC(T01_T04_T		log2FC(T01_T04_T		log2FC(T02_T03_T		log2FC(T01_T04_T		log2FC(T01_T04_T		log2FC(T07_T10_T	
Annotation	GeneID	12/T02_T03_T05)	Up/Down	12/T06_T09_T13)	Up/Down	05/T06_T09_T13)	Up/Down	12/T07_T10_T14)	Up/Down	12/T08_T11_T15)	Up/Down	14/T08_T11_T15)  Up/Down	
PYR/PYL	c258749.graph_c0 1.995782739	up	-		-		-		-		-	
	c258501.graph_c0 -3.137580934	down	-		-		-		-		-	
	c274368.graph_c2 -		-		-		2.041923217	up	1.86084961	up	-	
SnRK2	c269564.graph_c0 3.637206032	up	3.075008469	up	-		1.923171242	up	-		-	
ABF	c275663.graph_c1 1.740889832	up	-		-		-		-		-	
	c269678.graph_c1 -5.65939075	down	-3.174514314	down	2.504500349	up	-5.195754232	down	-3.879710987	down	-	
	c277233.graph_c0 -3.009253173	down	-2.442069659	down	-		-2.063013999	down	-1.312176619	down	-	


GA	


Annotation	log2FC(T01_T04_T
GeneID	12/T02_T03_T05)	

Up/Down	log2FC(T01_T04_T
12/T06_T09_T13)	

Up/Down	log2FC(T02_T03_T	log2FC(T01_T04_T
05/T06_T09_T13)   Up/Down	12/T07_T10_T14)	

Up/Down	log2FC(T01_T04_T
12/T08_T11_T15)	

Up/Down	log2FC(T07_T10_T
14/T08_T11_T15)  Up/Down	
GID1	c276105.graph_c0 -		-		-	-		1.41512934	up	-	
DELLA	c271286.graph_c0 -		-		-	-2.780153379	down	-3.404236101	down	-	
TF	c274996.graph_c0 1.346973546	up	2.050194187	up	-	1.944974913	up	2.125826909	up	-	
	c277171.graph_c0 1.854095017	up	2.530377027	up	-	2.175010666	up	1.998419749	up	-	
	c278213.graph_c0 1.634593535	up	2.619216837	up	-	-		2.316671211	up	-	
	c263572.graph_c0 6.990946185	up	7.559497224	up	-	3.380620088	up	-		-	
	c271626.graph_c1 4.481830614	up	-		-	2.761368793	up	2.28297646	up	-	


Auxin	
	log2FC(T01_T04_T		log2FC(T01_T04_T		log2FC(T02_T03_T		log2FC(T01_T04_T		log2FC(T01_T04_T		log2FC(T07_T10_T	
Annotation	GeneID	12/T02_T03_T05)	Up/Down	12/T06_T09_T13)	Up/Down	05/T06_T09_T13)	Up/Down	12/T07_T10_T14)	Up/Down	12/T08_T11_T15)	Up/Down	14/T08_T11_T15)  Up/Down	
AUX1	c274787.graph_c0 1.332187722	up	-		-2.370768618	down	-		-		-	
	c264621.graph_c1 -		-2.671116216	down	-3.035894269	down	-1.341081747	down	-1.617168333	down	-	
TIR1	c267124.graph_c0 -		1.410348653	up	1.401197712	up	2.041425797	up	-		-	
AUX/IAA	c272437.graph_c0 1.531336031	up	-		-		-		-		-	
	c273870.graph_c1 -		-		-		-		-1.302666688	down	-	
	c264173.graph_c0 -3.931235428	down	-3.391002695	down	-		-2.460118902	down	-2.169543247	down	-	
ARF	c268917.graph_c0 -2.112499539	down	-2.152969088	down	-		-1.684400242	down	-1.834202674	down	-	
	c270886.graph_c0 -2.031148352	down	-2.007528871	down	-		-1.60996696	down	-1.788536131	down	-	
	c270886.graph_c2 -1.65031099	down	-		-		-1.360176302	down	-1.640440572	down	-	
	c276328.graph_c1 -1.545109205	down	-1.369989209	down	-		-1.840052947	down	-2.342516751	down	-	
	c278499.graph_c0 -1.509075806	down	-		-		-1.40646065	down	-		-	
GH3	c272434.graph_c0 -9.902219079	down	-10.81849906	down	-		-8.243558705	down	-6.842973591	down	-	
	c271682.graph_c0		-2.24282023	down	-		-		-		-	
	c270580.graph_c0 -3.878502534	down	-3.904561702	down	-		-2.271371091	down	-3.231953942	down	-	
SAUR	c272387.graph_c1 1.92891872	up	2.241085213	up	-		2.099648736	up	3.116274241	up	-	
	c274941.graph_c1 -2.797779296	down	-		-		-5.398070282	down	-2.729438591	down	-	
	c260733.graph_c0 -		-4.47383236	down	-		-3.886319442	down	-4.026293826	down	-	
	c256166.graph_c0 -		3.05978781	up	-		3.76237429	up	4.090345646	up	-	

Ethylene


Annotation	GeneID	log2FC(T01_T04_T   Up/Down log2FC(T01_T04_T   Up/Down log2FC(T02_T03_T   Up/Down	log2FC(T01_T04_T  Up/Down	log2FC(T01_T04_T  Up/Down	log2FC(T07_T10_T  Up/Down


12/T02_T03_T05)		12/T06_T09_T13)	05/T06_T09_T13)	12/T07_T10_T14)	12/T08_T11_T15)		14/T08_T11_T15)	
EBF1/2	c277367.graph_c1 1.788589651	up	-	-	3.552410197	up	-		-	
c273785.graph_c0			-	2.725085732	up	2.100239409	up	-	
c276416.graph_c0 -2.006830754	down	-1.973614202	down	-	-1.758477695	down	-1.664099386	down	-	
EIN3	c272352.graph_c0 1.735064954	up	2.421717763	up	-	-	2.15897112	up	-	
c272744.graph_c1 2.262631023	up	3.480080642	up	-	-	2.643871879	up	-	
c275453.graph_c0 -		-	-	-	2.099835775	up	-	
ERF1/2	c262219.graph_c0 -		-	-	5.922878715	up	5.427333738	up	-	
c268171.graph_c0 -		-	-	4.422269538	up	4.458149662	up	-	
c242782.graph_c0 -		-	-	-2.890639363	down	-		-	


Cytokinine	
log2FC(T01_T04_T
Annotation	GeneID	12/T02_T03_T05)	

Up/Down	log2FC(T01_T04_T
12/T06_T09_T13)	log2FC(T02_T03_T	log2FC(T01_T04_T
Up/Down 05/T06_T09_T13)   Up/Down	12/T07_T10_T14)  Up/Down	log2FC(T01_T04_T
12/T08_T11_T15)	

Up/Down	log2FC(T07_T10_T
14/T08_T11_T15)  Up/Down	
CRE1	c277101.graph_c0 -		-	-	1.491780007	up	1.507211229	up	-	
AHP	c258918.graph_c0 -		-	-	4.075754394	up	-		-	
B-ARR	c274996.graph_c0 1.346973546	up	2.050194187	up	-	1.944974913	up	2.125826909	up	-	
c277171.graph_c0 1.854095017	up	2.530377027	up	-	2.175010666	up	1.998419749	up	-	
A-ARR	c269659.graph_c0 3.523279979	up	-	-	-	-		-	
c274421.graph_c0 -		-1.512630604	down	-	-	-		-	


Brassinosteriod	
Annotation	GeneID	log2FC(T01_T04_T	Up/Down	log2FC(T01_T04_T	Up/Down log2FC(T02_T03_T  Up/Down	log2FC(T01_T04_T Up/Down	log2FC(T01_T04_T	Up/Down	log2FC(T07_T10_T Up/Down	


12/T02_T03_T05)	12/T06_T09_T13)	05/T06_T09_T13)	12/T07_T10_T14)	12/T08_T11_T15)	14/T08_T11_T15)


Jasmonic acid


Annotation	log2FC(T01_T04_T
GeneID	12/T02_T03_T05)	

Up/Down	log2FC(T01_T04_T
12/T06_T09_T13)	

Up/Down	log2FC(T02_T03_T
05/T06_T09_T13)	

Up/Down	log2FC(T01_T04_T
12/T07_T10_T14)	

Up/Down	log2FC(T01_T04_T
12/T08_T11_T15)	

Up/Down	log2FC(T07_T10_T
14/T08_T11_T15)	

Up/Down	
JAR1	c272102.graph_c0 7.392792842	up	4.954673813	up	-2.430432381	down	7.186907641	up	6.83170245	up	-		
	c277356.graph_c2 2.040596268	up	-		-		-		-		-		
COI1	c269415.graph_c0 -2.887179931	down	-1.686643777	down	-		-1.67230884	down	-1.546093266	down	-		
JAZ	c262934.graph_c0 4.541943785	up	2.122457416	up	-		4.337945723	up	3.227113559	up	-		
	c263809.graph_c0 3.710861816	up	2.537466643	up	-		3.956942471	up	3.511437533	up	-		
	c272735.graph_c1 1.903278685	up	1.582933432	up	-		3.272995361	up	3.26809982	up	-		
	c264821.graph_c0 4.071609774	up	-		-		3.678622922	up	2.961699082	up	-		
	c268107.graph_c0 6.342037988	up	2.604998235	up	-3.726081645	down	4.385729422	up			-1.714364715	down	
	c270391.graph_c0 2.07363705	up	-		-		2.539739478	up	2.352414849	up	-		
	c268150.graph_c0 -		-		-		3.765165524	up	2.608829435	up	-		
MYC2	c269480.graph_c0 3.601853836	up	1.889382756	up	-		3.742974623	up	2.972572478	up	-		


Salicylic acid


Annotation	log2FC(T01_T04_T
GeneID	12/T02_T03_T05)	

Up/Down	log2FC(T01_T04_T
12/T06_T09_T13)	

Up/Down	log2FC(T02_T03_T	log2FC(T01_T04_T
05/T06_T09_T13)   Up/Down	12/T07_T10_T14)	

Up/Down	log2FC(T01_T04_T
12/T08_T11_T15)	

Up/Down	log2FC(T07_T10_T
14/T08_T11_T15)  Up/Down	
NPR1	c269286.graph_c0 1.714608114	up	-		-	1.393091317	up	1.38148111	up	-	
	c263476.graph_c1 -3.366674945	down	-		-	-		-		-	
	273290.graph_c2  -2.479305888	down	-1.986128056	down	-	-		-		-	
TGA	c268088.graph_c0 -3.697978772	down	-2.705199054	down	-	-		-		-	
	c271806.graph_c2 -3.816792977	down	-3.1794917	down	-	-2.549481489	down	-2.862059115	down	-	
	c268990.graph_c0 -		-		-	-		1.569377401	up	-	
	c273104.graph_c1 -3.546197295	down	-2.727606956	down	-	-		-		-	
	c272001.graph_c0 -3.201620674	down	-		-	-2.209245477	down	-		-	
PR-1	c252920.graph_c0 -		-2.520584492	down	-	-		-		-	
	c259721.graph_c0 -		-3.349234737	down	-	-		-		-	
	c276448.graph_c2 -		-		-	-		-2.211262255	down	-	
