# Supplementary material for: Investigating the molecular basis for heterophylly in the aquatic plant Potamogeton octandrus (Potamogetonaceae) with comparative transcriptomics
Source: PeerJ. 2018 Feb 28;6:e4448. doi: 10.7717/peerj.4448 (PMC5834931; doi:10.7717/peerj.4448)
Supplement: Supplemental Information 1 [file peerj-06-4448-s001.zip › Supplemental files/Additional file 12.doc]

Additional file 12. The candidate DEG IDs, annotations, and the primers used for qPCR analysis.

	
Gene ID	Annotation	Forward Primer (5'-3')	Reverse Primer (5'-3')	
c260025.graph_c1	NF-YB	GGGAGATGGAGGGGGAGAAA	AATACAGAGGCTGACGGCG	
c260078.graph_c1	guard cell differentiation	GGTTTTTGTCTACGCCAGGA	TCTGTTCTTGCAGGTTGGTG	
c260159.graph_c0	stomatal complex morphogenesis	CACCAATGCCACGGTCCAA	ACGGCGGCGTTTATGATGT	
c262330.graph_c0 c264173.graph_c0 c264621.graph_c1 c265373.graph_c0 c266852.graph_c0	stomatal complex morphogenesis AUX/IAA
AUX1 MYB
GA2ox	ATGGCAGAAAGAGGGCACA CTCTGCTGACGATGGATCAC ACCAATCTGGTTCCTTGCTG GACCAGCAGAAGATGAGAAGC CCTTTGTTACTTCGCATTTCC	CAGGAATCAAAAGGGTAGGTAAAAC TGTGTTGTTGCTGCTCTTCC GTGTATGTTCCCACCCATCC GCCCCTGTAAATGGGTTCC CTCTTCGCTGCTTTTATTCTTG	
c267010.graph_c0 c267324.graph_c0 c268319.graph_c0 c268628.graph_c0 c268917.graph_c0 c269000.graph_c0 c271037.graph_c1
c271065.graph_c0	GRF
stomatal complex morphogenesis NAC
GA20ox ARF NE-YA
wax biosynthetic process
cuticle development	GCACAGTCACCACTCTCTGC ACGCTCCGACCAGATGTTAG CCCAATAGAGCCACGATTTC CTCCTGGGATTGAGTTTGGA TCCAACACCCTCAGAACCTC GGTAGCGGTGGAGAAACAGA GGGTCTACTCCAAGCCCTTC
ATTGGTACTTCGGCAACGAC	CCGTCCTTCGTTAGATCCTG AAGTCGCTGTCGTCGTTCTC CATGGACCCATTTGGTTTTC TCGTGGAAAACTTGGAGACC GAAGCGCCATTCATAACCAT TTGTAATGCAACTCCCACCA ATTCTTGTTCAGGGCAGCAA
CCTTGGTCGTCTTCCTTCTG	
