# Supplementary material for: Investigating the molecular basis for heterophylly in the aquatic plant Potamogeton octandrus (Potamogetonaceae) with comparative transcriptomics
Source: PeerJ. 2018 Feb 28;6:e4448. doi: 10.7717/peerj.4448 (PMC5834931; doi:10.7717/peerj.4448)
Supplement: Supplemental Information 1 [file peerj-06-4448-s001.zip › Supplemental files/Additional file 13.pdf]

Additional file 13. Quantitative real-time polymerase chain reaction (qPCR) validation of RNA-seq data. (a) Expression profiles of the candidate genes by qPCR. The value of relative expression level on the y-axis was calculated according to the log(2−ΔΔCT) method. The expression level of each gene in shoots was arbitrarily set as 1, and the value of the y-axis was 0. (b) Correlation of gene expression results. The x-axis represents the value of log2 fold change by RNA-seq and the y-axis represents the value of log2 relative expression level by qPCR.

a

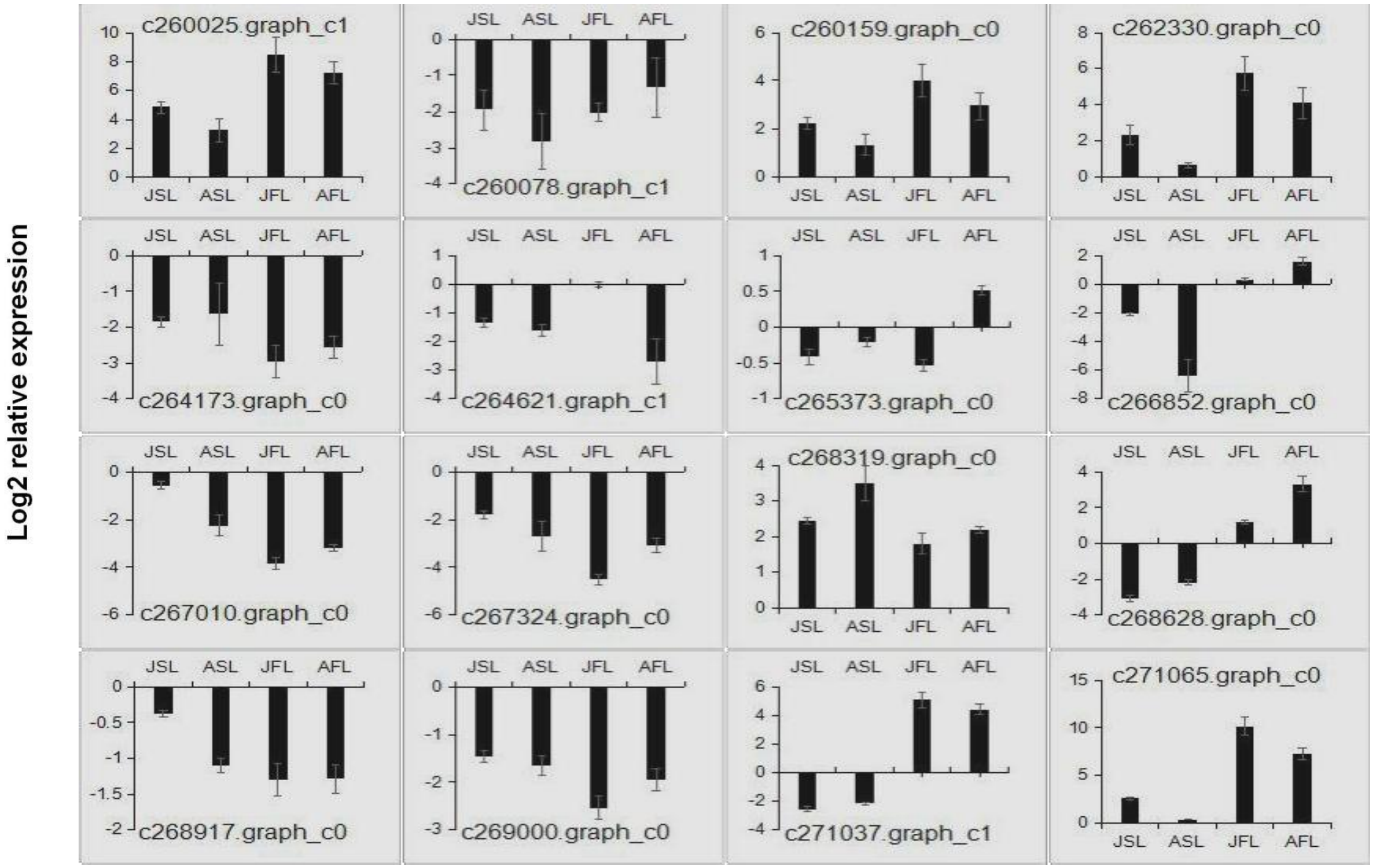

b

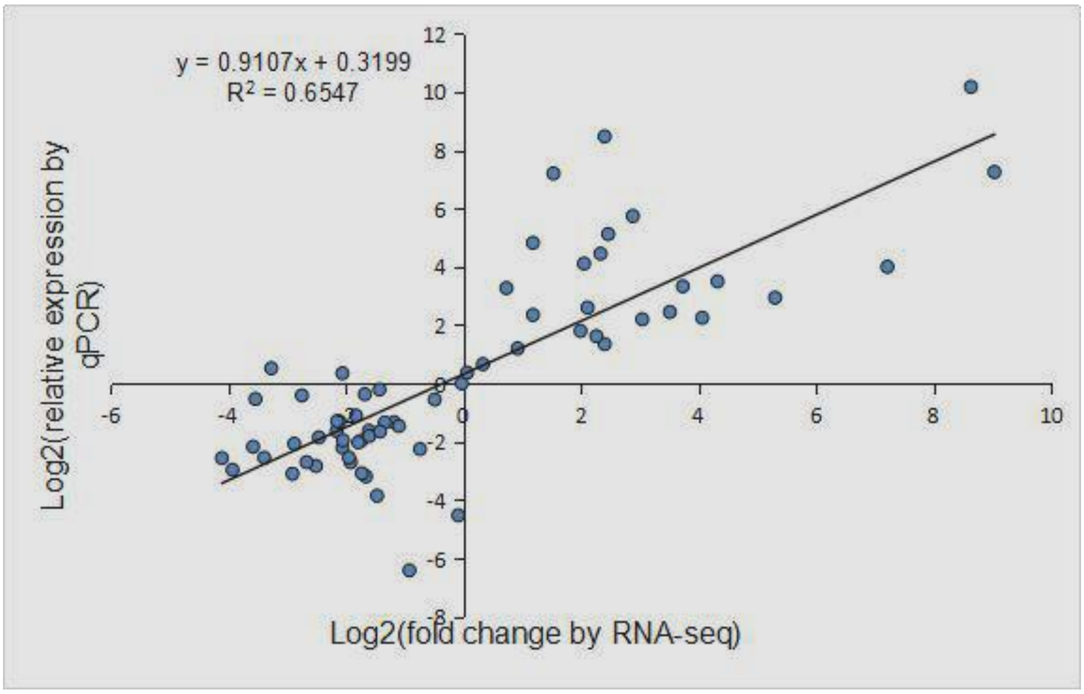

y = 0.9107x + 0.3199
R² = 0.6547
Log2(relative expression by qPCR)
Log2(fold change by RNA-seq)
