# Supplementary material for: Investigating the molecular basis for heterophylly in the aquatic plant Potamogeton octandrus (Potamogetonaceae) with comparative transcriptomics
Source: PeerJ. 2018 Feb 28;6:e4448. doi: 10.7717/peerj.4448 (PMC5834931; doi:10.7717/peerj.4448)
Supplement: Supplemental Information 1 [file peerj-06-4448-s001.zip › Supplemental files/Additional file 2.pdf]

Additional file 2. Alignment statistics of clean reads with assembled unigenes. Q30 percentage is proportion of nucleotides in reads with quality values > 30, GC percentage is proportion of guanine and cytosine nucleotides among total nucleotides.

Libraries	Clean reads	Mapped reads	Mapped Ratio	GC Content	%≥Q30
T01	31,857,874	22,904,147	71.89%	46.82%	96.01%
T02	33,502,995	25,268,322	75.42%	48.17%	96.22%
T03	24,505,452	18,654,035	76.12%	48.63%	96.41%
T04	34,683,841	25,018,483	72.13%	47.25%	96.22%
T05	29,049,232	21,178,061	72.90%	47.74%	96.16%
T06	33,120,060	24,384,370	73.62%	47.25%	96.12%
T07	28,157,357	20,984,070	74.52%	47.19%	96.02%
T08	29,642,773	21,414,638	72.24%	47.04%	96.18%
T09	31,810,475	23,614,411	74.23%	47.67%	96.23%
T10	32,188,101	23,541,376	73.14%	47.22%	96.47%
T11	35,671,865	25,532,937	71.58%	47.35%	96.21%
T12	31,080,852	22,133,012	71.21%	46.63%	96.19%
T13	20,951,664	15,866,895	75.73%	47.27%	96.23%
T14	29,066,748	21,229,060	73.04%	47.37%	96.17%
T15	29,911,305	21,699,902	72.55%	47.09%	96.08%

T01, T04, T12, T02, T03, T05, T06, T08, T13, T07, T10, T14, T08, T11 and T15 indicate three biological replicates of shoot, juvenile floating leaves, adult floating leaves, juvenile submerged and adult submerged leaves of *Potamogeton octandrus*, respectively; Mapped ratio means the ratio of mapped reads in clean reads.
