# Supplementary material for: Investigating the molecular basis for heterophylly in the aquatic plant Potamogeton octandrus (Potamogetonaceae) with comparative transcriptomics
Source: PeerJ. 2018 Feb 28;6:e4448. doi: 10.7717/peerj.4448 (PMC5834931; doi:10.7717/peerj.4448)
Supplement: Supplemental Information 1 [file peerj-06-4448-s001.zip › Supplemental files/Additional file 3.doc]

Additional file 3. Functional annotation of the P. octandrus.
	
Annotated_database	Annotated_number	300<=length<1000	length>=1000	
COG_annotation	20856	7108	8047	
GO_annotation	24025	7997	9787	
KEGG_annotation	22346	8547	8116	
KOG_annotation	30177	10306	12070	
Pfam_annotation	36985	12214	15990	
Swissprot_annotation	28515	9740	13104	
eggNOG_annotation	43041	13554	17857	
nr_annotation	42539	13750	18095	
All_annotated	48235	15313	18676	
