# Supplementary material for: Investigating the molecular basis for heterophylly in the aquatic plant Potamogeton octandrus (Potamogetonaceae) with comparative transcriptomics
Source: PeerJ. 2018 Feb 28;6:e4448. doi: 10.7717/peerj.4448 (PMC5834931; doi:10.7717/peerj.4448)

Additional file 4. Clusters of orthologous groups (COG) classifications for *P. octandrus*. In total, 20,856 of the 48,235 sequences with nr hits were grouped into 25 classifications.

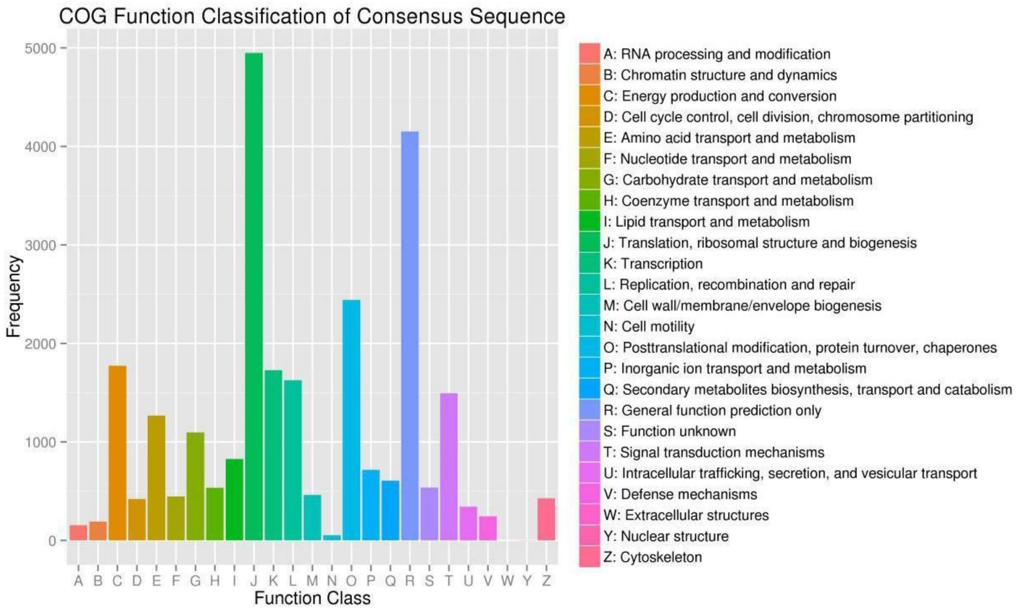

Supplement: Supplemental Information 1 [file peerj-06-4448-s001.zip › Supplemental files/Additional file 4.pdf]
