# Supplementary material for: Investigating the molecular basis for heterophylly in the aquatic plant Potamogeton octandrus (Potamogetonaceae) with comparative transcriptomics
Source: PeerJ. 2018 Feb 28;6:e4448. doi: 10.7717/peerj.4448 (PMC5834931; doi:10.7717/peerj.4448)

Additional file 5. Clusters of euKaryotic orthologous groups (KOG) classifications for *P. octandrus*. In total, 30,177 of the 48,235 sequences with nr hits were grouped into 25 classifications.

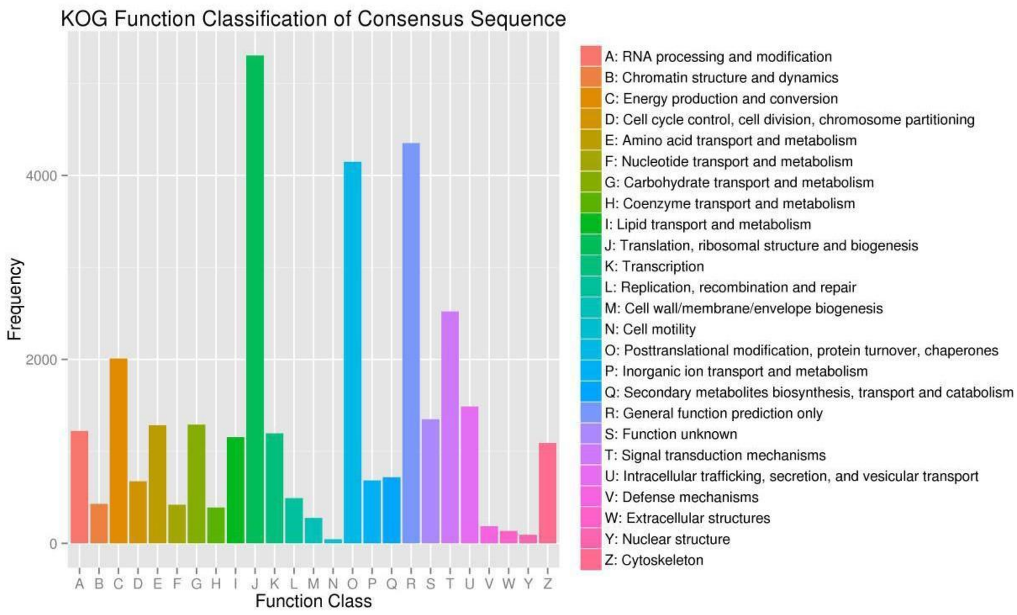

Supplement: Supplemental Information 1 [file peerj-06-4448-s001.zip › Supplemental files/Additional file 5.pdf]
