# Supplementary material for: Investigating the molecular basis for heterophylly in the aquatic plant Potamogeton octandrus (Potamogetonaceae) with comparative transcriptomics
Source: PeerJ. 2018 Feb 28;6:e4448. doi: 10.7717/peerj.4448 (PMC5834931; doi:10.7717/peerj.4448)
Supplement: Supplemental Information 1 [file peerj-06-4448-s001.zip › Supplemental files/Additional file 8.pdf]

Aditional file 8. A list of KEGG pathways mapped by 1,490; 1,141; 135, 1,425; 1,362; and 30 DEGs among all comparisons, respectively.

Pathway name	ko ID	Number	P-value	Corrected P-value
Plant hormone signal transduction	ko04075	49	6.83E-13	7.79E-11
Starch and sucrose metabolism	ko00500	44	2.68E-11	3.06E-09
Plant-pathogen interaction	ko04626	43	3.88E-09	4.43E-07
Amino sugar and nucleotide sugar metabolism	ko00520	34	1.96E-07	2.23E-05
Carotenoid biosynthesis	ko00906	13	2.27E-06	0.000259106
Phenylpropanoid biosynthesis	ko00940	23	4.46E-06	0.000507925
Terpenoid backbone biosynthesis	ko00900	19	9.96E-06	0.001135454
Linoleic acid metabolism	ko00591	7	0.000366893	0.041825762
alpha-Linolenic acid metabolism	ko00592	15	0.000960288	0.109472823
Fructose and mannose metabolism	ko00051	21	0.001070293	0.122013447
Zeatin biosynthesis	ko00908	5	0.001667145	0.190054578
Fatty acid elongation	ko00062	12	0.002235859	0.254887923
Cutin, suberine and wax biosynthesis	ko00073	5	0.003094339	0.352754654
Steroid biosynthesis	ko00100	11	0.003459126	0.394340402
Galactose metabolism	ko00052	13	0.00458357	0.522526963
Glycolysis / Gluconeogenesis	ko00010	39	0.005335074	0.608198425
Glycerophospholipid metabolism	ko00564	17	0.007364019	0.839498131
Pentose and glucuronate interconversions	ko00040	15	0.01463479	1
Flavonoid biosynthesis	ko00941	4	0.015037999	1
Phenylalanine metabolism	ko00360	15	0.01775181	1
Glycerolipid metabolism	ko00561	15	0.024042624	1
Histidine metabolism	ko00340	10	0.024804677	1
Pentose phosphate pathway	ko00030	21	0.029302514	1
Taurine and hypotaurine metabolism	ko00430	5	0.031972784	1
Ascorbate and aldarate metabolism	ko00053	12	0.034708677	1
Stilbenoid, diarylheptanoid and gingerol biosynthesis	ko00945	3	0.034957024	1
Carbon fixation in photosynthetic organisms	ko00710	32	0.037508248	1
Glycine, serine and threonine metabolism	ko00260	21	0.051897479	1
Diterpenoid biosynthesis	ko00904	3	0.052726187	1
Glyoxylate and dicarboxylate metabolism	ko00630	24	0.06954068	1
Ribosome biogenesis in eukaryotes	ko03008	26	0.072667899	1
Cyanoamino acid metabolism	ko00460	7	0.090663377	1
ABC transporters	ko02010	9	0.097851704	1
Butanoate metabolism	ko00650	8	0.106840962	1
Nicotinate and nicotinamide metabolism	ko00760	6	0.119038402	1
Biosynthesis of amino acids	ko01230	59	0.126092923	1
Protein export	ko03060	13	0.129705366	1

(A)

(A)

DNA replication	ko03030	9	0.132237796	1
Mismatch repair	ko03430	7	0.138284067	1
Carbon metabolism	ko01200	72	0.14847252	1
beta-Alanine metabolism	ko00410	11	0.169994798	1
Riboflavin metabolism	ko00740	3	0.17066125	1
Porphyrin and chlorophyll metabolism	ko00860	12	0.189112742	1
Inositol phosphate metabolism	ko00562	11	0.195587291	1
Pyrimidine metabolism	ko00240	26	0.204901111	1
Folate biosynthesis	ko00790	4	0.211237362	1
SNARE interactions in vesicular transport	ko04130	5	0.211748744	1
Alanine, aspartate and glutamate metabolism	ko00250	18	0.216812298	1
Phenylalanine, tyrosine and tryptophan biosynthesis	ko00400	8	0.22441094	1
Ether lipid metabolism	ko00565	4	0.238336611	1
Circadian rhythm - plant	ko04712	5	0.246697551	1
RNA degradation	ko03018	21	0.309176056	1
Degradation of aromatic compounds	ko01220	2	0.311254911	1
Sphingolipid metabolism	ko00600	6	0.33811372	1
Arginine and proline metabolism	ko00330	19	0.375911755	1
Pantothenate and CoA biosynthesis	ko00770	5	0.408420997	1
Other glycan degradation	ko00511	3	0.409605924	1
Vitamin B6 metabolism	ko00750	3	0.409605924	1
Peroxisome	ko04146	18	0.416060867	1
Valine, leucine and isoleucine biosynthesis	ko00290	5	0.420978267	1
Fatty acid biosynthesis	ko00061	7	0.424165055	1
Tryptophan metabolism	ko00380	8	0.429470577	1
Pyruvate metabolism	ko00620	21	0.439693432	1
Lipoic acid metabolism	ko00785	1	0.440463107	1
Lysine degradation	ko00310	8	0.44868407	1
2-Oxocarboxylic acid metabolism	ko01210	15	0.456082811	1
Thiamine metabolism	ko00730	2	0.468631121	1
Ubiquitin mediated proteolysis	ko04120	22	0.474986351	1
Ubiquinone and other terpenoid-quinone biosynthesis	ko00130	3	0.508579599	1
Synthesis and degradation of ketone bodies	ko00072	2	0.509643241	1
Tyrosine metabolism	ko00350	6	0.517185544	1
Tropane, piperidine and pyridine alkaloid biosynthesis	ko00960	3	0.524238441	1
C5-Branched dibasic acid metabolism	ko00660	2	0.52935627	1
Phosphatidylinositol signaling system	ko04070	12	0.534537877	1
Fatty acid degradation	ko00071	12	0.534537877	1
RNA polymerase	ko03020	7	0.535613794	1
One carbon pool by folate	ko00670	5	0.542241511	1
Selenocompound metabolism	ko00450	5	0.57630388	1
Cysteine and methionine metabolism	ko00270	17	0.614083388	1

(A)

(B)

Glutathione metabolism	ko00480	18	0.644584206	1
Aminoacyl-tRNA biosynthesis	ko00970	12	0.673295116	1
Nitrogen metabolism	ko00910	7	0.679142162	1
Lysine biosynthesis	ko00300	2	0.695900531	1
Glycosaminoglycan degradation	ko00531	1	0.706604009	1
Valine, leucine and isoleucine degradation	ko00280	12	0.715791691	1
Nucleotide excision repair	ko03420	7	0.717737626	1
Biosynthesis of unsaturated fatty acids	ko01040	6	0.723872515	1
Fatty acid metabolism	ko01212	13	0.741673483	1
RNA transport	ko03013	33	0.742012273	1
Brassinosteroid biosynthesis	ko00905	1	0.758284806	1
Isoquinoline alkaloid biosynthesis	ko00950	2	0.759087433	1
Basal transcription factors	ko03022	3	0.762242386	1
Endocytosis	ko04144	22	0.770902302	1
Sulfur metabolism	ko00920	6	0.774769784	1
Limonene and pinene degradation	ko00903	1	0.800870207	1
N-Glycan biosynthesis	ko00510	4	0.821520849	1
Homologous recombination	ko03440	3	0.835751248	1
Glycosylphosphatidylinositol(GPI)-anchor biosynthesis	ko00563	1	0.835959517	1
mRNA surveillance pathway	ko03015	15	0.847309108	1
Base excision repair	ko03410	3	0.855476001	1
Biotin metabolism	ko00780	1	0.86487107	1
Photosynthesis	ko00195	15	0.881372387	1
Sulfur relay system	ko04122	1	0.902192628	1
Propanoate metabolism	ko00640	5	0.908287543	1
Purine metabolism	ko00230	20	0.920382431	1
Arachidonic acid metabolism	ko00590	2	0.947508818	1
Regulation of autophagy	ko04140	2	0.955304052	1
Phagosome	ko04145	18	0.988721858	1
Protein processing in endoplasmic reticulum	ko04141	33	0.991944411	1
Citrate cycle (TCA cycle)	ko00020	10	0.994848143	1
Spliceosome	ko03040	25	0.999551122	1
Photosynthesis - antenna proteins	ko00196	3	0.999730918	1
Oxidative phosphorylation	ko00190	27	0.999996705	1
Ribosome	ko03010	124	1	1

(A)

(A list of KEGG pathways mapped by 1,141 DEGs in T01_T04_T12 and T06_T09_T13 comparison.)

Patyway name	ko ID	Number	P-value	Corrected P-value
Plant hormone signal transduction	ko04075	38	8.43E-13	9.62E-11
Starch and sucrose metabolism	ko00500	29	3.59E-06	0.00040947
Plant-pathogen interaction	ko04626	31	4.53E-06	0.000515892
Cutin, suberine and wax biosynthesis	ko00073	6	0.000107073	0.012206363
Phenylpropanoid biosynthesis	ko00940	17	0.00019181	0.021866354
Diterpenoid biosynthesis	ko00904	5	0.000390324	0.044496884
Glycerolipid metabolism	ko00561	17	0.000395809	0.045122209
Fatty acid elongation	ko00062	11	0.001035054	0.117996172
Amino sugar and nucleotide sugar metabolism	ko00520	21	0.001329174	0.151525819
Carotenoid biosynthesis	ko00906	8	0.001400948	0.159708118
Glycerophospholipid metabolism	ko00564	16	0.001647721	0.187840212
Glycolysis / Gluconeogenesis	ko00010	34	0.001871189	0.213315501
Terpenoid backbone biosynthesis	ko00900	12	0.00331381	0.377774395
Zeatin biosynthesis	ko00908	4	0.005111416	0.582701386
alpha-Linolenic acid metabolism	ko00592	11	0.007951017	0.906415907
Degradation of aromatic compounds	ko01220	4	0.010191802	1
Pentose and glucuronate interconversions	ko00040	13	0.010894343	1
Taurine and hypotaurine metabolism	ko00430	5	0.012575557	1
Fructose and mannose metabolism	ko00051	15	0.013017508	1
Phenylalanine metabolism	ko00360	13	0.013050248	1
Histidine metabolism	ko00340	9	0.015155956	1
Circadian rhythm - plant	ko04712	7	0.015960938	1
Glycine, serine and threonine metabolism	ko00260	19	0.018835874	1
Steroid biosynthesis	ko00100	8	0.019442286	1
Glyoxylate and dicarboxylate metabolism	ko00630	22	0.019763797	1
Brassinosteroid biosynthesis	ko00905	4	0.020862284	1
Biosynthesis of amino acids	ko01230	53	0.021263944	1
Arginine and proline metabolism	ko00330	21	0.033683781	1
Galactose metabolism	ko00052	9	0.036683712	1
Pentose phosphate pathway	ko00030	17	0.03779486	1
Valine, leucine and isoleucine biosynthesis	ko00290	7	0.048800775	1
Carbon metabolism	ko01200	60	0.082218335	1
Cyanoamino acid metabolism	ko00460	6	0.083125878	1
Fatty acid degradation	ko00071	14	0.088215873	1
2-Oxocarboxylic acid metabolism	ko01210	16	0.096734293	1
Riboflavin metabolism	ko00740	3	0.100967394	1

(A)

(C)

Other glycan degradation	ko00511	4	0.106474853	1
Thiamine metabolism	ko00730	3	0.122189119	1
Ascorbate and aldarate metabolism	ko00053	8	0.15582081	1
C5-Branched dibasic acid metabolism	ko00660	3	0.156684636	1
Tyrosine metabolism	ko00350	7	0.166136732	1
Porphyrin and chlorophyll metabolism	ko00860	10	0.171225805	1
beta-Alanine metabolism	ko00410	9	0.172731016	1
Flavonoid biosynthesis	ko00941	2	0.184166203	1
Biotin metabolism	ko00780	3	0.193747248	1
Carbon fixation in photosynthetic organisms	ko00710	22	0.20235276	1
Tryptophan metabolism	ko00380	8	0.204398661	1
Lysine degradation	ko00310	8	0.21740058	1
Glycosaminoglycan degradation	ko00531	2	0.239120143	1
SNARE interactions in vesicular transport	ko04130	4	0.240781343	1
Glutathione metabolism	ko00480	18	0.252481864	1
ABC transporters	ko02010	6	0.26410313	1
Vitamin B6 metabolism	ko00750	3	0.272862938	1
Nicotinate and nicotinamide metabolism	ko00760	4	0.273542727	1
Nitrogen metabolism	ko00910	8	0.279314472	1
Protein export	ko03060	9	0.297236774	1
Glycosphingolipid biosynthesis - globo series	ko00603	2	0.313184268	1
Ether lipid metabolism	ko00565	3	0.313683309	1
Endocytosis	ko04144	22	0.331452361	1
Limonene and pinene degradation	ko00903	2	0.349733349	1
Fatty acid biosynthesis	ko00061	6	0.359583529	1
Other types of O-glycan biosynthesis	ko00514	1	0.364674568	1
Valine, leucine and isoleucine degradation	ko00280	12	0.385247652	1
Pantothenate and CoA biosynthesis	ko00770	4	0.419702989	1
Mismatch repair	ko03430	4	0.441911483	1
Stilbenoid, diarylheptanoid and gingerol biosynthesis	ko00945	1	0.45386344	1
Inositol phosphate metabolism	ko00562	7	0.465589911	1
Homologous recombination	ko03440	4	0.4747201	1
Butanoate metabolism	ko00650	4	0.527645518	1
Lysine biosynthesis	ko00300	2	0.563506199	1
Folate biosynthesis	ko00790	2	0.563506199	1
Phosphatidylinositol signaling system	ko04070	9	0.599471757	1
Alanine, aspartate and glutamate metabolism	ko00250	11	0.609419831	1
Pyruvate metabolism	ko00620	15	0.612424378	1
Fatty acid metabolism	ko01212	11	0.637244818	1

(A)

(D)

Tropane, piperidine and pyridine alkaloid biosynthesis	ko00960	2	0.643803744	1
Biosynthesis of unsaturated fatty acids	ko01040	5	0.665185568	1
Peroxisome	ko04146	12	0.676109456	1
Linoleic acid metabolism	ko00591	1	0.686452245	1
Arachidonic acid metabolism	ko00590	3	0.702776129	1
Sphingolipid metabolism	ko00600	3	0.728369261	1
Phagosome	ko04145	20	0.732748508	1
Synthesis and degradation of ketone bodies	ko00072	1	0.743774106	1
One carbon pool by folate	ko00670	3	0.744450182	1
Selenocompound metabolism	ko00450	3	0.767136941	1
N-Glycan biosynthesis	ko00510	3	0.825476111	1
Cysteine and methionine metabolism	ko00270	11	0.833810256	1
Sulfur relay system	ko04122	1	0.837350305	1
RNA transport	ko03013	24	0.838695104	1
Phenylalanine, tyrosine and tryptophan biosynthesis	ko00400	3	0.841932319	1
DNA replication	ko03030	3	0.847111402	1
Sulfur metabolism	ko00920	4	0.851721534	1
RNA degradation	ko03018	11	0.86933177	1
Propanoate metabolism	ko00640	4	0.880210985	1
Isoquinoline alkaloid biosynthesis	ko00950	1	0.885801898	1
Ubiquinone and other terpenoid-quinone biosynthesis	ko00130	1	0.885801898	1
Base excision repair	ko03410	2	0.88922752	1
Nucleotide excision repair	ko03420	4	0.89088981	1
Regulation of autophagy	ko04140	2	0.893575041	1
Aminoacyl-tRNA biosynthesis	ko00970	7	0.898958214	1
Ubiquitin mediated proteolysis	ko04120	12	0.916140328	1
Protein processing in endoplasmic reticulum	ko04141	28	0.959679817	1
Photosynthesis	ko00195	9	0.971926727	1
Pyrimidine metabolism	ko00240	10	0.979984343	1
Citrate cycle (TCA cycle)	ko00020	8	0.987572265	1
mRNA surveillance pathway	ko03015	7	0.99263688	1
Ribosome biogenesis in eukaryotes	ko03008	7	0.994335185	1
RNA polymerase	ko03020	1	0.996164833	1
Photosynthesis - antenna proteins	ko00196	3	0.997184665	1
Purine metabolism	ko00230	9	0.998994891	1
Oxidative phosphorylation	ko00190	25	0.999442589	1
Spliceosome	ko03040	18	0.999523266	1
Proteasome	ko03050	1	0.999999997	1

(A)

Ribosome	ko03010	128	1	1

(A list of KEGG pathways mapped by 135 DEGs in T02_T03_T05 and T06_T09_T13 comparison.)

**Patyway name**	**ko ID**	**Number**	**P-value**	**Corrected P-value**
Phenylpropanoid biosynthesis	ko00940	7	6.76E-06	0.000385143
Phenylalanine metabolism	ko00360	7	7.86E-06	0.000448172
Starch and sucrose metabolism	ko00500	7	0.000281707	0.01605728
Plant hormone signal transduction	ko04075	6	0.000783586	0.044664395
Pentose and glucuronate interconversions	ko00040	4	0.005525598	0.314959064
alpha-Linolenic acid metabolism	ko00592	3	0.016243756	0.925894097
Glyoxylate and dicarboxylate metabolism	ko00630	5	0.018636641	1
Photosynthesis - antenna proteins	ko00196	4	0.023499779	1
Carotenoid biosynthesis	ko00906	2	0.025304423	1
Glycerophospholipid metabolism	ko00564	3	0.043245298	1
Diterpenoid biosynthesis	ko00904	1	0.074204132	1
Zeatin biosynthesis	ko00908	1	0.079291263	1
Cutin, suberine and wax biosynthesis	ko00073	1	0.089382832	1
Phenylalanine, tyrosine and tryptophan biosynthesis	ko00400	2	0.092528048	1
Degradation of aromatic compounds	ko01220	1	0.094387564	1
Glycosaminoglycan degradation	ko00531	1	0.099365124	1
Amino sugar and nucleotide sugar metabolism	ko00520	3	0.105258419	1
Ascorbate and aldarate metabolism	ko00053	2	0.116903744	1
Linoleic acid metabolism	ko00591	1	0.119006545	1
Glycosphingolipid biosynthesis - globo series	ko00603	1	0.119006545	1
Taurine and hypotaurine metabolism	ko00430	1	0.147677929	1
Nitrogen metabolism	ko00910	2	0.152166441	1
Inositol phosphate metabolism	ko00562	2	0.161770623	1
Glycerolipid metabolism	ko00561	2	0.173437715	1
Peroxisome	ko04146	3	0.183764215	1
Ether lipid metabolism	ko00565	1	0.197868452	1
Glutathione metabolism	ko00480	3	0.237791498	1
Carbon fixation in photosynthetic organisms	ko00710	3	0.330355333	1
Fatty acid elongation	ko00062	1	0.338927988	1
Base excision repair	ko03410	1	0.338927988	1
Sphingolipid metabolism	ko00600	1	0.342575404	1
Histidine metabolism	ko00340	1	0.3533988	1
Galactose metabolism	ko00052	1	0.398301794	1
Tyrosine metabolism	ko00350	1	0.398301794	1

(B)

(E)

Plant-pathogen interaction	ko04626	2	0.419238017	1
Biosynthesis of unsaturated fatty acids	ko01040	1	0.467378939	1
Tryptophan metabolism	ko00380	1	0.470325369	1
Photosynthesis	ko00195	2	0.511915588	1
Fructose and mannose metabolism	ko00051	1	0.589785366	1
Phosphatidylinositol signaling system	ko04070	1	0.652916237	1
Fatty acid degradation	ko00071	1	0.652916237	1
Glycolysis / Gluconeogenesis	ko00010	2	0.659925099	1
Aminoacyl-tRNA biosynthesis	ko00970	1	0.689567326	1
Phagosome	ko04145	2	0.718983073	1
Glycine, serine and threonine metabolism	ko00260	1	0.720833874	1
Alanine, aspartate and glutamate metabolism	ko00250	1	0.728531167	1
Fatty acid metabolism	ko01212	1	0.736018745	1
Ribosome biogenesis in eukaryotes	ko03008	1	0.819713677	1
Carbon metabolism	ko01200	4	0.819947392	1
Ubiquitin mediated proteolysis	ko04120	1	0.85110918	1
Purine metabolism	ko00230	1	0.904169728	1
Biosynthesis of amino acids	ko01230	2	0.942347517	1
Oxidative phosphorylation	ko00190	2	0.958986735	1
RNA transport	ko03013	1	0.96169784	1
Spliceosome	ko03040	1	0.98090429	1
Protein processing in endoplasmic reticulum	ko04141	1	0.986350307	1
Ribosome	ko03010	4	0.999999981	1

(F)

(A list of KEGG pathways mapped by 1,425 DEGs in T01_T04_T12 and T07_T10_T14 comparison.)

**Patyway name**	**ko ID**	**Number**	**P-value**	**Corrected P-value**
Plant hormone signal transduction	ko04075	46	7.84E-13	8.94E-11
Starch and sucrose metabolism	ko00500	46	2.14E-12	2.44E-10
Phenylpropanoid biosynthesis	ko00940	28	2.91E-09	3.31E-07
Plant-pathogen interaction	ko04626	41	1.80E-08	2.05E-06
Phenylalanine metabolism	ko00360	23	4.40E-06	0.000501775
Amino sugar and nucleotide sugar metabolism	ko00520	30	8.45E-06	0.000963658
Zeatin biosynthesis	ko00908	7	1.27E-05	0.001449188
alpha-Linolenic acid metabolism	ko00592	18	1.96E-05	0.00223374
Linoleic acid metabolism	ko00591	8	3.97E-05	0.004520281
Pentose and glucuronate interconversions	ko00040	19	0.000297279	0.033889758
Cutin, suberine and wax biosynthesis	ko00073	6	0.000347848	0.039654683
Glycerolipid metabolism	ko00561	19	0.000641732	0.073157475
Glycolysis / Gluconeogenesis	ko00010	41	0.00110939	0.126470461

(C)

(G)

Galactose metabolism	ko00052	14	0.001295723	0.147712464
Fructose and mannose metabolism	ko00051	20	0.001876955	0.213972817
Biosynthesis of amino acids	ko01230	68	0.004627166	0.527496978
Diterpenoid biosynthesis	ko00904	4	0.008373358	0.954562779
Glycerophospholipid metabolism	ko00564	16	0.012726962	1
Carotenoid biosynthesis	ko00906	7	0.018179718	1
Glyoxylate and dicarboxylate metabolism	ko00630	26	0.020452646	1
Histidine metabolism	ko00340	10	0.02128288	1
Ascorbate and aldarate metabolism	ko00053	12	0.029425847	1
Endocytosis	ko04144	34	0.032911242	1
Photosynthesis - antenna proteins	ko00196	19	0.033284555	1
Riboflavin metabolism	ko00740	4	0.048018359	1
Lysine degradation	ko00310	12	0.052795929	1
Other glycan degradation	ko00511	5	0.071988284	1
Fatty acid biosynthesis	ko00061	10	0.07889343	1
Cyanoamino acid metabolism	ko00460	7	0.081866139	1
Pyruvate metabolism	ko00620	26	0.082109452	1
Regulation of autophagy	ko04140	8	0.090543677	1
Carbon fixation in photosynthetic organisms	ko00710	29	0.095546604	1
Porphyrin and chlorophyll metabolism	ko00860	13	0.100443155	1
Carbon metabolism	ko01200	72	0.10384359	1
Tyrosine metabolism	ko00350	9	0.107348986	1
Circadian rhythm - plant	ko04712	6	0.10900567	1
Phenylalanine, tyrosine and tryptophan biosynthesis	ko00400	9	0.112819671	1
Sulfur metabolism	ko00920	11	0.119256048	1
Steroid biosynthesis	ko00100	7	0.125911001	1
Photosynthesis	ko00195	24	0.138566546	1
Terpenoid backbone biosynthesis	ko00900	9	0.148763205	1
Tryptophan metabolism	ko00380	10	0.162851352	1
Fatty acid degradation	ko00071	15	0.188390574	1
Limonene and pinene degradation	ko00903	3	0.192890282	1
Cysteine and methionine metabolism	ko00270	21	0.212286237	1
Ether lipid metabolism	ko00565	4	0.224970823	1
beta-Alanine metabolism	ko00410	10	0.245804544	1
Ubiquinone and other terpenoid-quinone biosynthesis	ko00130	4	0.265409619	1
RNA degradation	ko03018	21	0.270261194	1
Tropane, piperidine and pyridine alkaloid biosynthesis	ko00960	4	0.279182066	1
ABC transporters	ko02010	7	0.288761861	1

(C)

(H)

Biotin metabolism	ko00780	3	0.292098703	1
Nitrogen metabolism	ko00910	9	0.360645877	1
SNARE interactions in vesicular transport	ko04130	4	0.377447865	1
Pantothenate and CoA biosynthesis	ko00770	5	0.387597713	1
Vitamin B6 metabolism	ko00750	3	0.39391328	1
Valine, leucine and isoleucine biosynthesis	ko00290	5	0.399888376	1
Inositol phosphate metabolism	ko00562	9	0.404596875	1
Folate biosynthesis	ko00790	3	0.410622054	1
Glycosphingolipid biosynthesis - globo series	ko00603	2	0.413007587	1
Nicotinate and nicotinamide metabolism	ko00760	4	0.419483948	1
Arginine and proline metabolism	ko00330	18	0.42951367	1
Other types of O-glycan biosynthesis	ko00514	1	0.432135378	1
Pentose phosphate pathway	ko00030	14	0.445062286	1
Thiamine metabolism	ko00730	2	0.45544645	1
Arachidonic acid metabolism	ko00590	5	0.460726881	1
Fatty acid elongation	ko00062	5	0.484587027	1
Isoquinoline alkaloid biosynthesis	ko00950	3	0.491569295	1
Synthesis and degradation of ketone bodies	ko00072	2	0.4960619	1
Fatty acid metabolism	ko01212	15	0.497661605	1
Glutathione metabolism	ko00480	19	0.5059592	1
C5-Branched dibasic acid metabolism	ko00660	2	0.51563266	1
2-Oxocarboxylic acid metabolism	ko01210	14	0.527588018	1
Stilbenoid, diarylheptanoid and gingerol biosynthesis	ko00945	1	0.52978938	1
Taurine and hypotaurine metabolism	ko00430	2	0.534692204	1
Protein export	ko03060	9	0.543081679	1
Selenocompound metabolism	ko00450	5	0.553651567	1
Protein processing in endoplasmic reticulum	ko04141	45	0.603075844	1
Flavonoid biosynthesis	ko00941	1	0.634408454	1
Glycine, serine and threonine metabolism	ko00260	13	0.650520089	1
Degradation of aromatic compounds	ko01220	1	0.677643331	1
Glycosaminoglycan degradation	ko00531	1	0.697305813	1
Butanoate metabolism	ko00650	4	0.697977412	1
Biosynthesis of unsaturated fatty acids	ko01040	6	0.701135964	1
Brassinosteroid biosynthesis	ko00905	1	0.749391961	1
Alanine, aspartate and glutamate metabolism	ko00250	12	0.776024447	1
Valine, leucine and isoleucine degradation	ko00280	11	0.78187091	1
Propanoate metabolism	ko00640	6	0.799369382	1
Phosphatidylinositol signaling system	ko04070	9	0.82973478	1
Base excision repair	ko03410	3	0.843374272	1

(C)

(I)

Sphingolipid metabolism	ko00600	3	0.849754914	1
One carbon pool by folate	ko00670	3	0.861839701	1
Sulfur relay system	ko04122	1	0.896232657	1
Peroxisome	ko04146	12	0.90049787	1
Basal transcription factors	ko03022	2	0.90350321	1
Lysine biosynthesis	ko00300	1	0.908520142	1
mRNA surveillance pathway	ko03015	13	0.926558044	1
Mismatch repair	ko03430	2	0.92884966	1
Ubiquitin mediated proteolysis	ko04120	15	0.932160291	1
Homologous recombination	ko03440	2	0.939026684	1
Citrate cycle (TCA cycle)	ko00020	13	0.94696871	1
Aminoacyl-tRNA biosynthesis	ko00970	8	0.948296232	1
Purine metabolism	ko00230	18	0.95870836	1
RNA polymerase	ko03020	3	0.967297357	1
N-Glycan biosynthesis	ko00510	2	0.976401792	1
Pyrimidine metabolism	ko00240	13	0.982777495	1
RNA transport	ko03013	24	0.986216175	1
DNA replication	ko03030	1	0.997335273	1
Nucleotide excision repair	ko03420	2	0.997550899	1
Phagosome	ko04145	14	0.999025784	1
Ribosome biogenesis in eukaryotes	ko03008	6	0.999882381	1
Spliceosome	ko03040	22	0.999909044	1
Oxidative phosphorylation	ko00190	26	0.999996749	1
Ribosome	ko03010	150	1	1

(J)

(A list of KEGG pathways mapped by 1,362 DEGs in T01_T04_T12 and T08_T11_T15 comparison.)

Patyway name	ko ID	Number	P-value	Corrected P-value
Plant hormone signal transduction	ko04075	46	5.75E-13	6.38E-11
Starch and sucrose metabolism	ko00500	40	1.57E-09	1.74E-07
alpha-Linolenic acid metabolism	ko00592	21	2.52E-07	2.80E-05
Plant-pathogen interaction	ko04626	37	1.01E-06	0.000112309
Phenylpropanoid biosynthesis	ko00940	22	8.94E-06	0.000992626
Cutin, suberine and wax biosynthesis	ko00073	7	3.28E-05	0.003641726
Linoleic acid metabolism	ko00591	8	3.75E-05	0.004163785
Amino sugar and nucleotide sugar metabolism	ko00520	27	0.000131438	0.014589588
Fatty acid biosynthesis	ko00061	16	0.000276793	0.030724022
Carotenoid biosynthesis	ko00906	9	0.001278646	0.141929701
Biotin metabolism	ko00780	7	0.002108646	0.234059697
Phenylalanine metabolism	ko00360	17	0.002512706	0.278910356

(C)

(K)

Biosynthesis of amino acids	ko01230	69	0.002546626	0.282675482
Galactose metabolism	ko00052	13	0.003476927	0.385938896
Glycerolipid metabolism	ko00561	17	0.003717374	0.412628556
Fatty acid degradation	ko00071	21	0.00579316	0.643040726
Diterpenoid biosynthesis	ko00904	4	0.008149649	0.90461105
Steroid biosynthesis	ko00100	10	0.008203979	0.910641678
Glycolysis / Gluconeogenesis	ko00010	37	0.008945012	0.992896332
Zeatin biosynthesis	ko00908	4	0.010589481	1
Fatty acid metabolism	ko01212	24	0.010638206	1
Glycerophospholipid metabolism	ko00564	16	0.011891771	1
Porphyrin and chlorophyll metabolism	ko00860	16	0.01347704	1
Nitrogen metabolism	ko00910	14	0.019968623	1
Valine, leucine and isoleucine biosynthesis	ko00290	9	0.020697081	1
Photosynthesis	ko00195	28	0.022830031	1
Taurine and hypotaurine metabolism	ko00430	5	0.028267559	1
Circadian rhythm - plant	ko04712	7	0.042924104	1
Pantothenate and CoA biosynthesis	ko00770	8	0.048167331	1
2-Oxocarboxylic acid metabolism	ko01210	20	0.060474164	1
Fructose and mannose metabolism	ko00051	15	0.063110733	1
Glycine, serine and threonine metabolism	ko00260	20	0.064945638	1
Vitamin B6 metabolism	ko00750	5	0.070121046	1
Lysine biosynthesis	ko00300	5	0.07692163	1
Cyanoamino acid metabolism	ko00460	7	0.079293276	1
C5-Branched dibasic acid metabolism	ko00660	4	0.085746278	1
Degradation of aromatic compounds	ko01220	3	0.091264693	1
Endocytosis	ko04144	31	0.097318258	1
Histidine metabolism	ko00340	8	0.103839702	1
Arginine and proline metabolism	ko00330	22	0.118811368	1
Glyoxylate and dicarboxylate metabolism	ko00630	22	0.122046052	1
Peroxisome	ko04146	21	0.13793102	1
Brassinosteroid biosynthesis	ko00905	3	0.144656338	1
Other glycan degradation	ko00511	4	0.182833723	1
SNARE interactions in vesicular transport	ko04130	5	0.193536826	1
Ascorbate and aldarate metabolism	ko00053	9	0.198280029	1
Carbon metabolism	ko01200	68	0.211583505	1
Ether lipid metabolism	ko00565	4	0.220946243	1
Pyruvate metabolism	ko00620	23	0.222719351	1
beta-Alanine metabolism	ko00410	10	0.238572162	1
Biosynthesis of unsaturated fatty acids	ko01040	9	0.251779525	1
Lysine degradation	ko00310	9	0.276054818	1

(C)

(L)

Arachidonic acid metabolism	ko00590	6	0.278829369	1
ABC transporters	ko02010	7	0.282216406	1
Carbon fixation in photosynthetic organisms	ko00710	25	0.305598256	1
Sulfur metabolism	ko00920	9	0.309388465	1
Photosynthesis - antenna proteins	ko00196	14	0.311028771	1
Pentose phosphate pathway	ko00030	15	0.326941659	1
Valine, leucine and isoleucine degradation	ko00280	15	0.339840501	1
Alanine, aspartate and glutamate metabolism	ko00250	16	0.347970779	1
Pentose and glucuronate interconversions	ko00040	9	0.378258804	1
Terpenoid backbone biosynthesis	ko00900	7	0.391106702	1
Glycosphingolipid biosynthesis - globo series	ko00603	2	0.40914049	1
Riboflavin metabolism	ko00740	2	0.40914049	1
Nicotinate and nicotinamide metabolism	ko00760	4	0.41368319	1
Limonene and pinene degradation	ko00903	2	0.451400598	1
Tyrosine metabolism	ko00350	6	0.484886274	1
Ubiquinone and other terpenoid-quinone biosynthesis	ko00130	3	0.486340058	1
Regulation of autophagy	ko04140	5	0.489471623	1
Stilbenoid, diarylheptanoid and gingerol biosynthesis	ko00945	1	0.526964694	1
Inositol phosphate metabolism	ko00562	8	0.540347027	1
Tryptophan metabolism	ko00380	7	0.5481453	1
Glutathione metabolism	ko00480	18	0.58861715	1
Flavonoid biosynthesis	ko00941	1	0.631476826	1
Phenylalanine, tyrosine and tryptophan biosynthesis	ko00400	5	0.669590767	1
Fatty acid elongation	ko00062	4	0.672037385	1
Folate biosynthesis	ko00790	2	0.678203674	1
Sphingolipid metabolism	ko00600	4	0.682179928	1
Butanoate metabolism	ko00650	4	0.69210051	1
One carbon pool by folate	ko00670	4	0.701798771	1
Purine metabolism	ko00230	23	0.719597785	1
Tropane, piperidine and pyridine alkaloid biosynthesis	ko00960	2	0.754000524	1
Thiamine metabolism	ko00730	1	0.789917244	1
N-Glycan biosynthesis	ko00510	4	0.801154394	1
Phosphatidylinositol signaling system	ko04070	9	0.823015871	1
Base excision repair	ko03410	3	0.839524568	1
Protein processing in endoplasmic reticulum	ko04141	40	0.843231658	1
Citrate cycle (TCA cycle)	ko00020	15	0.853714993	1
Cysteine and methionine metabolism	ko00270	13	0.886020282	1

(C)

(M)

Propanoate metabolism	ko00640	5	0.892838864	1
Basal transcription factors	ko03022	2	0.901070863	1
RNA degradation	ko03018	13	0.915611234	1
Mismatch repair	ko03430	2	0.926844954	1
Ubiquitin mediated proteolysis	ko04120	15	0.927470024	1
Isoquinoline alkaloid biosynthesis	ko00950	1	0.931799859	1
Homologous recombination	ko03440	2	0.937217604	1
RNA transport	ko03013	27	0.94166787	1
Protein export	ko03060	5	0.947222304	1
Nucleotide excision repair	ko03420	4	0.960318749	1
RNA polymerase	ko03020	3	0.965932609	1
Pyrimidine metabolism	ko00240	13	0.981253943	1
Selenocompound metabolism	ko00450	1	0.993686337	1
mRNA surveillance pathway	ko03015	9	0.99495663	1
DNA replication	ko03030	1	0.997206891	1
Aminoacyl-tRNA biosynthesis	ko00970	4	0.999056936	1
Spliceosome	ko03040	21	0.999950701	1
Ribosome biogenesis in eukaryotes	ko03008	5	0.999968746	1
Oxidative phosphorylation	ko00190	26	0.999995815	1
Phagosome	ko04145	7	0.999999639	1
Proteasome	ko03050	4	0.999999926	1
Ribosome	ko03010	178	0.999999987	1

(N)

(A list of KEGG pathways mapped by 30 DEGs in T07_T10_T14 and T08_T11_T15 comparison.)

Patyway name	ko ID	Number	P-value	Corrected P-value
Ribosome	ko03010	14	8.89E-05	0.000622134
Zeatin biosynthesis	ko00908	1	0.019906441	0.139345086
Circadian rhythm - plant	ko04712	1	0.06991647	0.489415288
Sulfur metabolism	ko00920	1	0.150219793	1
Glycerolipid metabolism	ko00561	1	0.168398036	1
Plant hormone signal transduction	ko04075	1	0.23540973	1
Ubiquitin mediated proteolysis	ko04120	1	0.370950525	1

(C)
