# Supplementary material for: Investigating the molecular basis for heterophylly in the aquatic plant Potamogeton octandrus (Potamogetonaceae) with comparative transcriptomics
Source: PeerJ. 2018 Feb 28;6:e4448. doi: 10.7717/peerj.4448 (PMC5834931; doi:10.7717/peerj.4448)
Supplement: Supplemental Information 1 [file peerj-06-4448-s001.zip › Supplemental files/Additional file 9.pdf]

Additional file 9. A list of putative transcription factors (TF) that were differentially expressed during heterophyllous leaf development. 469 putative TF genes were identified in 42 families.

AP2 Family

GeneID	T01_FPKM	T04_FPKM	T012_FPKM	T02_FPKM	T03_FPKM	T05_FPKM	T06_FPKM	T09_FPKM	T013_FPKM	T07_FPKM	T10_FPKM	T14_FPKM	T08_FPKM	T11_FPKM	T15_FPKM
c258655.graph_c0	20.486107	14.49863186	19.73934967	68.14869079	216.46457	71.60241146	86.23033867	157.6858557	109.5555854	62.79411009	150.598571	83.81646275	119.7860552	251.7514196	122.6925423
c271761.graph_c1	2.384167174	1.351176397	1.529415399	31.1509648	5.827744104	35.39672252	19.35477783	21.83159352	47.41065975	51.43947229	39.24004002	61.54151998	32.06663059	47.6339468	44.17010556
c276354.graph_c0	67.49979312	53.79993669	70.02538769	10.36515531	12.64042055	22.94581082	25.24323158	31.63158183	30.13283501	114.6781695	87.40729385	58.52562361	72.18781681	105.1850965	81.23004481
c273737.graph_c0	5.620997795	4.991262795	3.792250915	114.881842	65.30936412	198.5254317	81.99632488	54.70062416	71.40005279	335.0987798	235.5707182	343.7389629	276.204521	188.3156334	209.8253236
c274576.graph_c0	0.679396592	3.346975619	1.789008392	82.74738097	54.179832	38.73630745	16.60975646	6.919809818	8.897195804	17.34451671	105.6840786	69.46840121	24.13095724	21.22449044	65.74212301
c274534.graph_c0	102.1551176	96.7398697	108.6209259	34.31930048	48.6425697	28.90673269	35.52646432	37.22312119	33.38406605	36.13286155	47.68613541	45.0420801	44.35115549	70.55316861	53.85507282
c260047.graph_c0	1.08303656	0.35059808	0.21646192	21.62336892	4.82495915	7.599091666	9.293924854	3.608972194	8.647653152	42.02809385	33.26613676	43.90573483	21.19772703	25.05431517	46.91681596
c259122.graph_c0	0.385361982	0.301340499	1.250668873	2.889246884	7.670248818	2.904663301	5.569214893	6.005754916	2.772855662	13.96441478	17.2235063	12.3904216	7.762547928	10.11662878	8.936536373
c272749.graph_c0	2.452725449	2.279047053	3.822624328	5.363539976	5.662251077	8.852432884	6.658385141	9.844413732	9.44520561	8.702896871	10.9225804	14.20922152	11.69315634	9.771866948	10.79641085
c269493.graph_c0	4.120761728	1.392352388	7.801314233	2.175104517	2.145089186	1.561227653	0.964976874	1.510274673	0.988358826	16.49074388	13.08649437	11.01177966	11.57528317	16.55427767	11.78417132
c269541.	3.283015795	1.786056757	5.753962617	10.73812682	6.44196405	17.12416041	1.918468975	5.437589467	14.43374737	11.18141702	8.4867096	9.568358989	11.32527285	3.779946351	18.76340421

graph_c0															
c271309. graph_c0	44.8051811	71.66323256	59.31744929	74.94576192	170.9050468	200.6751384	513.042951	292.8446516	199.1711477	372.8314093	439.5204293	404.4416044	722.335256	568.3173101	341.4240847
c259843. graph_c1	0	0	0	2.191052895	7.438442799	4.369038636	0.245202385	0	0.941789181	0.213738399	0.635858051	0.634273819	1.468061118	0.058664643	0.068983144
c262219. graph_c0	0.344726089	0.353803479	0.311480625	10.41073182	2.580560342	72.89357333	4.318842194	1.123223714	2.480461828	28.8975397	8.875950983	28.39909874	33.55608064	9.07743511	7.994190846
c262237. graph_c0	0.718514223	8.314463588	0.820068058	239.6700346	15.76049746	88.89235142	50.83151603	7.666892923	33.03382405	140.8003928	133.8921163	168.1902401	145.749755	43.15402391	399.6168839
c273150. graph_c0	7.392167822	4.384539785	6.050037885	18.16162574	9.062501255	7.910486952	37.00066079	68.75863062	105.0955898	93.60660252	69.57764551	95.1071231	78.67103643	149.9061771	90.46451157
c268772. graph_c0	1.919407238	1.114314824	1.390013429	6.857026368	8.317117251	7.300862976	12.72542508	7.027123551	10.69008255	20.29896959	18.77037612	20.48188445	13.81984898	31.01537205	20.07462499
c267961. graph_c0	1.262520804	6.156128383	1.438597684	547.4143571	117.5357641	231.1556102	50.2808968	9.792978502	75.87052344	270.2013896	215.5583418	292.874137	192.7169121	95.33999793	640.2575261
c251825. graph_c1	18.25759842	22.71316465	23.11244388	192.6964492	94.05074284	84.1080127	95.0730837	135.6693555	214.7548416	319.3379498	387.6763868	369.5027808	269.9806433	650.7809811	786.0292375
c265780. graph_c0	0.774423528	0.519487444	0.564494476	84.62611418	62.85753317	47.80137083	89.28828416	129.7388273	191.3276366	6.025622046	8.254738559	9.001909795	9.975691608	3.654801367	3.841468566
c269264. graph_c0	87.32294484	154.9110445	124.5532984	10.04207781	5.889783685	8.182857156	6.988074087	4.384061542	12.03643375	14.73028247	11.45816014	21.73148285	6.731791806	6.812197106	7.52195328
c273737. graph_c1	2.164560507	2.197221792	1.57409239	48.93851985	27.83336772	74.45319209	25.61552244	22.25110135	29.2634555	127.6487756	91.91534606	117.3698092	103.8545282	71.00054041	77.57581139
c260032. graph_c0	2.783976442	3.691582194	3.275985823	84.88898067	121.9760045	109.2782019	37.8122875	111.3840833	32.09775949	62.90733934	110.1798334	77.21644551	150.9422742	171.1233834	80.70044492

c271042. graph_c1	0.415981918	15.9389302	0.585762899	302.4961602	14.65125423	109.5782491	113.4587776	33.11367562	105.7953113	133.8015429	117.1739733	274.9686444	200.0019504	35.49274394	314.9499251
c274936. graph_c0	3.808995854	4.01451415	3.322556429	15.39215213	63.1785769	28.99050473	24.26599682	30.3091587	18.92987767	20.29583079	58.75439287	22.77671636	36.1393384	69.20107224	48.47954821
c274599. graph_c0	22.73187869	11.00275952	16.12739833	0.304731528	2.67533818	1.518501648	1.025213488	1.361459453	1.060151691	12.51123579	5.848876845	4.329262427	7.059935912	3.207536607	6.079107404
c271217. graph_c0	9.894130562	11.58004704	6.324854334	185.3274252	82.14004524	141.305249	92.31463701	93.86146758	102.900508	352.5910586	407.6900999	410.7034835	314.0822595	413.1547936	570.8948288
c278861. graph_c1	412.6560684	521.6894936	391.2605006	196.8439332	148.8641229	161.019046	191.3364187	235.3663054	155.5345445	133.3289733	93.99101227	75.00033616	99.03442651	96.60578583	98.40281783
c267545. graph_c2	0.446731103	0.724534532	0.615083052	3.997417608	1.213297915	8.765494472	1.813291734	0.335444022	0.785750668	6.916870477	7.041276632	16.83771701	10.60457367	5.339444217	5.755380568
c273976. graph_c0	23.08207097	27.45861228	24.30887965	2.59725468	3.191326821	9.315042327	23.31997305	17.47311744	9.609469475	17.14309753	11.98813793	12.14931003	44.56510943	26.1230999	12.33337661
c277074. graph_c2	6.80033854	8.341021738	9.471939271	28.17334945	52.82692683	35.61204574	21.8223276	20.06779717	24.80715544	16.33465096	30.92379863	24.01320682	24.0416717	26.69637862	30.370947
c268171. graph_c0	0	0.110491516	0.125066887	4.456746343	1.036156419	45.2099975	1.1344697	0.467704808	0.63907721	1.889890756	1.098310547	2.60850981	1.85438645	1.519961787	2.808625717
c264426. graph_c0	2.018562764	0.598495712	0.729556843	3.516080619	4.378994391	3.749386812	0.803582704	0.58463101	0	4.230278728	5.001235526	3.695388898	3.180488387	3.799904468	1.702197404
c269779. graph_c0	22.11327452	25.8905434	20.48202017	0.515470363	1.993744535	2.063268733	5.020707554	4.749710311	6.893746464	7.047436758	4.453108968	3.346149984	6.804399909	5.152989851	4.148742248
c274671. graph_c0	12.48996536	6.300263721	11.08537568	1.835508986	3.20340825	2.797785515	2.391101937	3.300580043	3.935956713	8.957439208	5.137638348	4.638861972	5.50235556	4.821740177	6.174353325
c278502.	15.9122301	13.15077756	17.28767987	19.28009022	17.13380286	22.53007201	49.84928317	36.25493343	43.39443147	22.68444514	18.51423074	22.73202261	25.78736025	21.39604169	28.72004234

graph_c0															
c262844.graph_c0	2.180111206	2.757345775	1.432626022	6.545829054	3.633381454	8.749811749	11.55645709	5.166162482	1.497385618	43.42280188	40.9685318	22.78362461	65.89536357	15.9452578	4.282698512
c264629.graph_c0	0.029588054	0.18895129	0.0611076	36.25107528	3.97779919	6.212693061	8.148227001	2.085247412	2.469635057	50.8943386	30.47313465	57.28950291	23.76806206	11.56415785	28.53745038
c242782.graph_c0	8.094236825	4.161013602	12.07328863	9.300604514	9.657044824	4.772911076	17.93168162	5.271602102	16.0858125	0.699360909	0.936249266	1.521938185	2.607645167	1.497234535	4.401456337
c256220.graph_c0	0	0.128081356	0.144977072	38.74682872	24.53693441	26.07765171	12.5589478	10.50438251	65.86527094	2.980444877	2.932809268	1.436293075	0.974817649	2.391196232	4.88362277
c249970.graph_c0	0.987340482	0.840696318	0.135942269	27.21934799	34.35083781	27.50022841	4.007637529	9.087199388	4.357344612	32.46159679	37.54124365	14.03491691	20.95325437	10.91587464	9.505477353
c261641.graph_c0	68.60684589	10.06019613	35.45911334	15.72530638	61.92761157	29.4597389	352.6994464	477.253929	144.88324	96.7629003	70.59265804	41.78858811	407.2535907	418.8977968	108.2559764
c262333.graph_c0	0.040733329	0.111482471	0.294439981	8.587992613	7.318145113	4.649268888	1.907740528	0.314599646	0.527570872	3.059813744	2.928710859	4.693563055	4.742812355	2.300390597	6.526362246
c259713.graph_c0	18.0078792	11.84485996	16.23266829	54.06084609	37.20824569	34.656697	44.45307068	47.35319075	78.89036849	29.94850319	5.896695453	13.60682144	13.86928402	10.88065276	8.91416357
c268527.graph_c0	10.91242608	6.783737295	7.179995977	0.196612544	0.560622153	0.623911531	4.658536402	6.223181624	5.802133844	7.017624613	3.893909354	2.573872591	5.13210452	1.926122979	3.002911731
c277205.graph_c0	0.553873929	0.918721032	0.7279392	42.60059667	193.848554	56.49562892	71.31311753	311.9868475	35.57853998	54.10425021	61.15375382	26.0814832	139.8463457	196.9763592	67.72462234
c273921.graph_c0	0.684538814	1.38476425	0.76834998	38.21540534	9.566647336	35.22060607	9.645966162	3.333086359	5.696528506	34.99371009	50.08561366	35.09565337	23.62210481	18.67580833	109.1759967

ARF family

GeneID	T01_FPKM	T04_FPKM	T012_FPKM	T02_FPKM	T03_FPKM	T05_FPKM	T06_FPKM	T09_FPKM	T013_FPKM	T07_FPKM	T10_FPKM	T14_FPKM	T08_FPKM	T11_FPKM	T15_FPKM
c277575.graph_c0	2.386909277	1.429027722	3.533546776	3.830342708	8.46678506	11.90719666	4.751100751	5.121846379	3.515488086	7.724610603	13.11775087	6.908016339	8.396205472	11.45894218	10.09355305
c272898.graph_c0	54.26319082	43.41957759	56.61125713	7.598978696	10.17832867	16.6378222	17.54783059	25.0473314	24.49724949	37.44437068	26.10705036	22.58064254	24.71153552	18.0766283	29.86898858
c270886.graph_c2	14.79985614	12.2579074	16.13360031	1.876413132	4.096713113	5.475221487	5.08220912	5.759136875	6.213721019	6.679893123	6.292880536	4.925095062	5.613723726	4.710886329	5.120278228
c278499.graph_c0	13.74425757	7.213804588	11.7200622	2.279695769	3.841262669	2.900830363	3.247759637	4.069815543	5.492240032	4.969546611	4.410988688	3.438338474	3.570540974	3.711837035	4.312123732
c268917.graph_c2	36.42522044	13.61494725	35.61641451	2.125648923	5.606975267	5.713692945	3.934864299	6.136672538	6.124856121	9.357140691	8.028840608	5.803504967	7.557641376	6.565136773	8.914272056
c272664.graph_c0	30.05506517	17.62656036	26.34654086	1.419566442	2.099004643	1.77817777	2.815250648	4.17701382	6.820505675	8.180006987	5.744128966	5.335025645	7.197240006	2.546160983	6.109857506
c274716.graph_c1	6.590269547	7.828160945	10.62260188	7.597951309	26.35079045	33.33820904	28.06092191	58.55353112	15.4054831	12.05482576	30.84594172	15.02249186	27.69479728	37.3501035	24.06674114
c276328.graph_c1	20.41052134	15.9187984	16.57595847	3.957611414	5.642378121	5.183737272	3.257539607	9.309940157	8.741847228	7.373703662	4.751224705	3.990374223	4.071815461	2.232594257	5.458670597
c270886.graph_c0	24.21272719	13.902906	22.46648556	2.494853798	3.737319156	5.829740509	4.695652734	4.936453889	5.627034432	9.00485065	7.354758126	4.642629215	6.961446128	5.52729718	6.65801551
c277982.graph_c0	7.045404388	5.109029816	8.532107668	17.85219621	15.72241618	25.00128136	9.164367268	10.40924204	10.97363416	15.94059142	14.60543368	16.53595245	14.14810813	12.32443834	16.65698583
c268917.graph_c0	20.63339091	9.917082357	23.93315217	1.187786629	3.960622828	4.947086843	3.150763942	3.881030284	5.135825575	7.993769919	5.57988857	4.064053525	6.134655693	4.81709313	5.404932983

ARR-B Family

GeneID	T01_FPKM	T04_FPKM	T012_FPKM	T02_FPKM	T03_FPKM	T05_FPKM	T06_FPKM	T09_FPKM	T013_FPKM	T07_FPKM	T10_FPKM	T14_FPKM	T08_FPKM	T11_FPKM	T15_FPKM
c277171. graph_c0	3.900803257	3.086292643	5.311420736	11.86984458	6.848840993	17.56950273	16.8142825	34.35995633	21.31185669	17.21222732	19.33055001	20.40877408	16.34803375	16.48322737	21.08054331
c274996. graph_c0	8.937006696	6.90125976	11.70859146	11.85860358	17.35875089	28.33404701	22.06095407	49.58205136	44.73021084	27.63834715	45.8000506	35.00519853	36.75740344	34.582385	60.66309498

B3 Family

GeneID	T01_FPKM	T04_FPKM	T012_FPKM	T02_FPKM	T03_FPKM	T05_FPKM	T06_FPKM	T09_FPKM	T013_FPKM	T07_FPKM	T10_FPKM	T14_FPKM	T08_FPKM	T11_FPKM	T15_FPKM
c277575. graph_c0	2.386909277	1.429027722	3.533546776	3.830342708	8.46678506	11.90719666	4.751100751	5.121846379	3.515488086	7.724610603	13.11775087	6.908016339	8.396205472	11.45894218	10.09355305
c259122. graph_c0	0.385361982	0.301340499	1.250668873	2.889246884	7.670248818	2.904663301	5.569214893	6.005754916	2.772855662	13.96441478	17.2235063	12.3904216	7.762547928	10.11662878	8.936536373
c274580. graph_c3	7.15053159	3.8710263	4.543952654	0.426612936	0.480176294	1.522994493	2.94412552	3.641300406	6.106313383	1.454259303	1.145204553	1.184660597	1.1751262	0.774819868	1.656550242
c272898. graph_c0	54.26319082	43.41957759	56.61125713	7.598978696	10.17832867	16.6378222	17.54783059	25.0473314	24.49724949	37.44437068	26.10705036	22.58064254	24.71153552	18.0766283	29.86898858
c269493. graph_c0	4.120761728	1.392352388	7.801314233	2.175104517	2.145089186	1.561227653	0.964976874	1.510274673	0.988358826	16.49074388	13.08649437	11.01177966	11.57528317	16.55427767	11.78417132
c271309. graph_c0	44.8051811	71.66323256	59.31744929	74.94576192	170.9050468	200.6751384	513.042951	292.8446516	199.1711477	372.8314093	439.5204293	404.4416044	722.335256	568.3173101	341.4240847
c271105. graph_c0	11.35904811	6.745121624	9.318784292	0.653930968	1.336187024	1.037561142	1.093506596	1.896293972	1.92000497	4.922290974	6.913652682	3.651754095	4.196399839	2.441799077	3.379132699
c270886. graph_c2	14.79985614	12.2579074	16.13360031	1.876413132	4.096713113	5.475221487	5.08220912	5.759136875	6.213721019	6.679893123	6.292880536	4.925095062	5.613723726	4.710886329	5.120278228

c278499. graph_c0	13.74425757	7.213804588	11.7200622	2.279695769	3.841262669	2.900830363	3.247759637	4.069815543	5.492240032	4.969546611	4.410988688	3.438338474	3.570540974	3.711837035	4.312123732
c272664. graph_c0	30.05506517	17.62656036	26.34654086	1.419566442	2.099004643	1.77817777	2.815250648	4.17701382	6.820505675	8.180006987	5.744128966	5.335025645	7.197240006	2.546160983	6.109857506
c274340. graph_c0	3.156623809	2.716766963	1.558074175	0.323358321	1.23745568	0.748208501	1.338929139	1.916655406	0.885677862	1.037439866	0.173605341	0.192414228	0.254487518	2.420336431	1.360243751
c274716. graph_c1	6.590269547	7.828160945	10.62260188	7.597951309	26.35079045	33.33820904	28.06092191	58.55353112	15.4054831	12.05482576	30.84594172	15.02249186	27.69479728	37.3501035	24.06674114
c276328. graph_c1	20.41052134	15.9187984	16.57595847	3.957611414	5.642378121	5.183737272	3.257539607	9.309940157	8.741847228	7.373703662	4.751224705	3.990374223	4.071815461	2.232594257	5.458670597
c270886. graph_c0	24.21272719	13.902906	22.46648556	2.494853798	3.737319156	5.829740509	4.695652734	4.936453889	5.627034432	9.00485065	7.354758126	4.642629215	6.961446128	5.52729718	6.65801551
c277982. graph_c0	7.045404388	5.109029816	8.532107668	17.85219621	15.72241618	25.00128136	9.164367268	10.40924204	10.97363416	15.94059142	14.60543368	16.53595245	14.14810813	12.32443834	16.65698583
c274580. graph_c4	6.127007961	4.879844797	6.728705662	1.848047045	0.475446506	0.418886898	2.550734872	2.628961713	5.178428821	1.376408162	1.322911526	1.047313896	0.519442447	0.523082597	1.435203914
c267307. graph_c0	6.484985509	4.902012207	6.751320294	1.245466648	2.005711151	1.425088451	0.669431406	1.047719717	1.333212454	3.630859658	3.034728214	2.593900355	3.081973923	2.965951633	1.980974813
c275671. graph_c0	14.51247194	7.252733401	9.913322311	0.101797594	0.137494541	0.646069592	1.194287092	1.773964166	1.888803872	1.592175831	1.34811525	0.726895372	1.562264526	2.252248761	2.174056352
c272877. graph_c0	3.789397295	3.008424531	2.748118816	0.170136712	0.282828133	0.373773908	0.880605917	0.432861418	0.811749161	0.803036005	0.534007449	0.513986557	0.602549864	0.492678456	0.9299859
c268917. graph_c0	20.63339091	9.917082357	23.93315217	1.187786629	3.960622828	4.947086843	3.150763942	3.881030284	5.135825575	7.993769919	5.57988857	4.064053525	6.134655693	4.81709313	5.404932983

BBR-BPC Family

GeneID	T01_FPKM	T04_FPKM	T012_FPKM	T02_FPKM	T03_FPKM	T05_FPKM	T06_FPKM	T09_FPKM	T013_FPKM	T07_FPKM	T10_FPKM	T14_FPKM	T08_FPKM	T11_FPKM	T15_FPKM
c267730.graph_c0	67.7875555	41.90129076	50.76199433	16.41266948	10.40770376	17.01505623	22.66290116	26.05878903	20.57229408	33.00002631	17.62334164	10.16362717	27.65113783	21.24745329	18.01747176
c262200.graph_c0	23.65188272	12.01474477	18.38729148	0.729608916	3.654406362	1.121459624	1.353196627	2.692089443	1.885584748	11.15549611	6.267425149	2.966715365	5.23968827	4.366889332	6.268216719

C2H2 Family

GeneID	T01_FPKM	T04_FPKM	T012_FPKM	T02_FPKM	T03_FPKM	T05_FPKM	T06_FPKM	T09_FPKM	T013_FPKM	T07_FPKM	T10_FPKM	T14_FPKM	T08_FPKM	T11_FPKM	T15_FPKM
c249410.graph_c0	3.629143394	5.355787356	4.739608833	8.113165406	19.56817142	14.48187008	21.99618103	18.23966311	19.96902936	15.33893244	32.04634916	23.44900123	35.80258592	41.81362059	42.64244526
c278216.graph_c2	9.55937698	8.942459502	10.90553842	22.73710694	10.0142072	23.23365446	25.75411471	20.68435918	18.99751008	29.13960175	21.52468301	29.41240348	30.01853945	27.74791354	36.75502672
c274119.graph_c0	11.5907244	7.682453179	9.575234078	2.119022298	1.618960677	3.387622672	5.317715935	8.403895681	7.625194212	9.06469414	6.182464748	3.668140501	8.262696261	8.693796735	6.707249452
c272321.graph_c3	85.51578409	83.02727285	75.93419049	30.52952507	36.63587945	27.10762618	36.9921679	33.11548614	48.74047606	57.82744065	43.37247242	26.17850848	40.66135259	32.60969483	41.67383692
c268062.graph_c0	4.911716722	4.540681763	8.926779604	17.83710531	14.72731734	23.23573193	44.35164562	19.06240531	40.05432063	44.73853618	98.21403441	101.3439405	52.81758451	85.03577487	119.9083828
c274527.graph_c2	0	0.03410232	0.077201782	2.097133221	2.329981366	1.56979158	3.676522177	7.398108454	6.616708484	0	0	0.120764343	0.039930802	0	0
c268092.graph_c0	9.147627221	5.068812118	8.970723336	26.24882307	26.5619054	28.6906779	28.12097939	22.93778563	27.16787696	25.73231809	27.20081407	19.9663235	38.824282	42.51398391	42.87318537
c263667.	5.910493146	3.235268977	5.141660622	5.413050489	2.40788775	7.482907803	27.41329399	15.00886455	11.90803619	24.6463632	37.44957683	27.88990544	39.22140758	95.85068817	55.05223781

graph_c0															
c274705.graph_c2	0.296073417	0.918361689	0.12229487	6.96560105	4.848841504	33.15596297	11.81431125	4.744886616	4.772059288	10.83015533	33.48476581	31.1822351	24.8588766	37.79379746	36.82638615
c271146.graph_c0	17.86501381	17.15331843	15.17415069	4.665044488	2.66188455	6.441944437	11.59508895	11.36513399	9.363295835	12.6359075	7.286049607	4.661191796	8.893952989	8.748684162	5.899321536
c276952.graph_c3	2.622142192	0.985010324	3.312985122	43.65760373	53.95239406	32.3870073	111.5671933	203.9479759	191.1167981	10.94840628	9.981054247	11.42785513	11.03930307	7.328149368	10.86079107
c269783.graph_c1	14.34460166	9.398839031	11.68756056	0.844074431	1.900104277	2.232087877	5.980431328	6.063778956	4.086875024	9.049462042	4.813657304	3.95116017	5.735109292	4.162378239	5.440758192
c260096.graph_c0	0.449679824	0.546987704	0.567547756	14.28676193	4.457768659	12.75082041	3.603719758	0.820026994	6.830820761	17.62389492	21.79726996	23.62078809	15.63824631	22.39453364	252.6435937
c278928.graph_c0	31.28397637	34.18795299	35.99797261	43.15528403	44.40494071	47.47120152	47.60079658	84.65933292	57.69197959	77.04742401	142.4321025	138.9639757	83.11182263	102.9341265	120.152204
c263548.graph_c0	1.890910395	0.548885469	1.745532176	42.1794002	9.769334748	27.6724969	29.73487844	14.35531433	19.13081123	50.3727351	42.45137162	65.90179334	58.51597397	35.10804565	55.41671293
c262501.graph_c0	1.114270417	2.764998686	1.15063991	0	0	0	0.041749376	0.215148801	0.256565924	1.067503394	0.519668876	0.143992813	0.666558335	1.518257077	0.892651362
c269911.graph_c2	6.096816652	1.431826954	6.389317351	0.218487998	0.995976986	0.454997166	1.696299011	1.136409112	0.912135696	2.03722711	1.935488389	1.917665026	2.643779033	2.029196734	1.877074269

C3H Family

GeneID	T01_FPKM	T04_FPKM	T012_FPKM	T02_FPKM	T03_FPKM	T05_FPKM	T06_FPKM	T09_FPKM	T013_FPKM	T07_FPKM	T10_FPKM	T14_FPKM	T08_FPKM	T11_FPKM	T15_FPKM
c270519.graph_c0	2.509715251	0.846157025	2.3099326	16.39043707	16.87022649	12.86588376	3.475160954	12.06203086	3.69022518	6.533641411	6.202222601	5.992882469	5.478403397	6.700362683	6.038562929

c277186. graph_c0	58.24589684	34.02774817	35.42640462	5.074341133	18.63180477	17.50631955	25.3592434	20.22259881	6.578198769	13.9800706	14.19395517	6.952426461	28.21512763	40.57470408	12.79091988
c261539. graph_c0	6.252176893	11.44783068	17.73194408	3.147428237	15.1210868	6.021095618	48.25342754	74.04732911	36.11654186	11.83956804	11.46496085	18.15978311	75.86418844	138.2197746	22.78778347
c272140. graph_c1	21.99755138	21.18705029	30.1554805	101.8826125	40.8893744	58.55631446	54.09723821	60.8990635	64.58144163	96.66838747	105.0413764	112.4599603	81.01543699	97.32404455	163.8468416
c268786. graph_c0	19.85925384	20.62924821	25.75083797	3.34895981	6.784988811	3.329122013	1.839072713	1.721177582	2.403257833	7.591624631	5.70096628	3.329429266	5.231178326	5.279975266	8.611256086
c270816. graph_c0	6.814111583	2.828099012	3.956156369	0.793890139	0.178713392	0.755776619	2.273687664	3.980987074	2.483097219	0.031838259	0.36939545	0.094480796	0.18744079	0.367022325	0.092480944
c269516. graph_c0	4.310672137	7.654193759	7.534760273	25.0018606	24.56873508	23.75174434	30.52967792	29.86222331	53.56150722	10.73643171	15.52743046	19.90439479	15.56624645	39.52762199	21.87690915
c274743. graph_c1	8.74497146	3.158708875	10.07237484	2.774940389	4.787789978	2.812153021	0.833965403	2.311212258	3.359746625	5.75100017	4.248379165	4.090781908	2.282545371	2.589405755	3.962482958
c267301. graph_c0	76.00451468	109.1684288	68.48042822	18.29621057	11.43668625	23.26266029	24.88663285	21.00209778	9.26633953	27.38472054	15.37753264	11.56759915	22.65972825	13.89070854	14.52610574
c268793. graph_c0	57.48675254	68.05281226	60.84879755	12.90544381	13.34078759	18.15264541	65.21489839	82.79575181	29.88675339	32.72213623	25.19252329	24.21698818	41.22844593	33.58846931	16.58318452
c265661. graph_c0	18.36539028	6.549979907	16.17114552	14.71164752	15.13338871	21.59626905	37.57329028	28.50425254	15.79778881	35.68305242	27.19584777	38.77042142	32.92860613	36.0508971	40.47245023
c265345. graph_c0	1.907039959	1.831351096	1.710168763	0.59034431	1.165369295	0.756542138	0.188033652	1.501952594	1.299981265	0.71025703	0.585129928	0.108087423	0.268043782	0.49485733	0.317398687
c277000. graph_c0	3.493666322	2.304630736	2.109116147	0.437720019	1.116736631	0.810261107	0.553809173	0.622683916	0.309397251	3.276816228	2.088925385	1.67855291	1.148305907	0.915446131	1.30308751
c278325.	9.169700411	5.118578071	9.794932827	264.01426	260.673884	243.3172254	121.6664017	133.6094002	227.3634176	48.52752696	61.69451174	83.65076263	50.76080548	19.96190537	26.77137773

graph_c1															
c262799. graph_c1	20.18709161	2.70453394	8.940938712	0	0	0.050669352	0.088154777	1.362875082	0	0.76842894	0.228602689	0.050674029	2.865173024	2.404376326	0.496014236
c250126. graph_c0	1.994043415	0.385881657	2.37111763	15.8017194	19.6442784	13.79384753	10.58428851	6.241985886	10.52190635	7.104612855	4.520706005	4.945420592	9.423951924	5.63332925	3.503168593
c274230. graph_c2	19.00764571	10.64846624	14.9592422	25.50692795	16.63362485	22.35488894	40.14636415	38.39341233	53.37962925	28.17668946	19.31982784	15.55050226	22.12773643	33.87085348	25.9200599
c275793. graph_c0	12.77691725	7.362595504	10.07067273	24.55137118	15.99431275	29.37473733	30.25194798	34.78341229	36.37089139	28.30154119	34.18939714	34.55703096	30.66977305	32.57494776	37.12259168
c276931. graph_c0	35.42802616	13.52006526	29.63208222	6.909314128	2.70934116	11.00688423	27.99412732	17.67621822	24.28098914	18.3238197	13.32224749	8.266977169	24.20665469	21.233574	24.44906686
c260585. graph_c0	0.995060781	0.813880015	0.283459118	1.800799818	1.258075313	2.844927658	4.853197355	1.52379933	2.567694479	5.677945134	5.300826693	5.727330968	7.440586774	8.397023811	13.67165501
c263953. graph_c0	0.136526039	0.041517353	0.422946638	2.38840549	2.002306278	0.931057978	0.980440999	0.659027942	0.916876121	0.792702466	2.122415171	0.980151482	1.750073431	2.814844778	2.878214549
c276799. graph_c0	10.66764432	9.489044206	10.83704962	22.57145843	18.96250509	39.45077071	26.59638461	23.82482752	23.61212702	32.92677055	34.71774854	37.04498199	37.70000618	30.35942188	36.75219928
c267690. graph_c0	1.652013454	1.391191445	1.312257492	0.206980087	0.124249311	0	0	0.073610508	0.03657536	0.221353403	0.074082622	0.164217881	0.135746851	0.205047207	0.294693521
c279070. graph_c0	2.10219738	1.196266913	2.20520166	2.71583393	2.721884348	9.121692262	8.827910633	4.726152912	4.253327783	6.612008394	5.5977445	7.750791893	6.759043423	4.309979679	4.760905157
c263215. graph_c0	6.83913895	3.471167725	8.256006083	13.16151426	11.5961302	8.297780764	2.030135333	2.371382409	5.683492972	7.602861467	8.891224034	10.42504915	3.910085118	6.346606083	4.67066361
c278319. graph_c2	2.499057967	2.72594201	5.49785659	5.751620101	11.35398674	9.242819581	16.58958384	13.68917705	10.39820873	23.06636167	38.42773145	26.56093272	33.48527329	23.9117365	13.91561649

c266431. graph_c0	2.580066088	2.686080746	3.479234625	13.0738859	8.755030867	9.347744391	24.82135818	9.055005511	10.26521758	27.29530453	35.39060771	16.80134286	41.11409117	58.8816928	53.7525245
c270153. graph_c0	14.2271857	4.795342712	15.27053494	20.99117692	13.87623834	23.62088637	37.1896653	40.22473171	16.69203753	34.94495072	34.21794295	40.11392904	37.50768193	56.60553238	53.19038553
c261051. graph_c0	2.62646623	1.732573818	2.461837084	7.422331297	2.370464609	7.527200045	15.0261723	7.606964871	8.606143272	10.95352247	10.48249334	11.35711289	10.83407212	21.98661043	9.66856455
c275978. graph_c1	19.37334888	7.040849602	20.46809198	6.018236324	10.33381848	12.86258341	2.602998244	4.590051554	3.240981328	14.85414893	10.69776796	9.786109887	7.386026582	6.446081547	10.49521891
c263435. graph_c0	6.646487149	6.371130635	5.620052334	0.479392904	10.3599945	1.244667115	0.496255304	1.766912383	0.970352459	4.194682188	2.620571294	1.192916072	1.543454652	0.906658012	2.132259474
c263751. graph_c0	6.04634129	7.274788709	6.831866193	40.87502858	15.74326028	25.38193557	23.06477806	15.39690002	33.16558845	36.29896315	40.61222797	50.3439122	25.55440363	36.56574095	41.10373218
c266778. graph_c0	16.76729189	8.008283423	12.09473772	29.85354837	87.93728917	48.21684026	98.16175974	58.9893552	37.40098284	57.76865698	113.6323547	83.64375796	171.5778821	211.8419391	80.15785016
c277430. graph_c0	1.457435008	0.57700103	1.292032856	5.125855758	8.267652392	5.507508327	7.00618539	3.849458702	4.926869636	4.565403457	6.091763637	4.930563271	4.832131742	3.426409409	5.405922847
c266656. graph_c0	0.090569653	0	0	0	0	0.09752402	0.141393957	0.116584222	0	0.098600405	0.439994836	0.227577049	0.80623379	1.271951157	1.432028443
c262171. graph_c0	1.986828694	1.331109345	3.077517392	29.15839641	17.68072788	18.15135997	13.40543059	6.653524476	25.95643082	6.421404714	5.067393159	7.856285954	12.73418662	12.16110428	28.14210507
c262313. graph_c0	1.231069256	1.16320933	1.770671082	0	0	0	0	0	0	0	0	0	0	0	0
c272247. graph_c1	11.61446393	8.118556949	7.940178929	21.04347039	18.23652892	14.90910394	19.84453987	17.52013832	24.02677132	15.10292676	12.12071593	15.43162292	21.51081062	19.81063363	15.55841841
c274524.	13.76406068	13.2023919	14.36496147	32.31450684	21.94542377	28.13245478	30.37489751	34.61881966	30.12467454	64.16742522	68.89045992	88.94816643	57.54279974	73.9105317	83.15331788

graph_c0

c275995.graph_c0	6.280296904	4.791472121	7.374858519	5.707992931	6.486925014	4.757707641	3.487995598	11.93850876	6.038611305	1.542900446	1.755011484	1.077316243	1.632653269	2.540873444	2.343362107
c277520.graph_c0	28.23261546	30.2057779	33.462887	163.6271738	122.0266419	107.364351	129.8160604	89.87830716	120.055338	105.894605	226.9266643	197.5628026	101.82133	107.6693897	119.838552
c278384.graph_c0	4.801634922	3.157817365	5.459643673	4.879602813	4.398108032	10.65882836	10.61650123	12.54163415	10.82818487	11.54621908	15.45204135	17.37617557	12.45660102	18.74962198	20.54576492
c267070.graph_c0	10.78546362	11.54777878	12.33632626	73.26353214	32.22202004	37.50232295	44.33970481	42.951774	102.9900833	145.5494399	178.585319	154.2576605	118.2124863	256.4448408	281.8921894
c265006.graph_c0	21.04290604	17.58464279	27.45178907	1.661095721	6.855399761	1.793659646	0.923448028	2.986584412	2.592861555	6.6986862	5.185726271	3.807302438	5.556048575	4.723434431	5.195899574
c272639.graph_c1	25.39108301	12.03727353	20.42628684	5.116838996	9.946621151	7.879861224	11.69455031	6.700981377	7.435669046	12.16752555	7.691992005	8.716408465	12.19951328	9.183935821	7.363155491
c278920.graph_c0	7.31316796	5.45272937	8.335024507	18.77398153	13.85938446	24.22687829	45.76966251	38.0036538	18.6319398	15.53007696	15.64985249	7.44763303	17.45655898	20.9133769	10.84933351
c277996.graph_c0	14.18955067	9.788963241	13.640187	2.745383127	3.459381702	3.266976555	3.30983268	5.143800328	4.525958761	3.746124575	4.871216584	5.020451671	4.960283936	6.733028283	5.733215799
c270099.graph_c0	5.418387164	5.555783961	6.814717696	8.192505971	9.791834152	7.903916236	15.35630846	20.96888556	13.7290278	10.13389083	10.66417805	13.34064812	13.50536591	19.01344339	19.55079274
c276057.graph_c1	3.720078541	2.200382353	2.955000713	14.24155066	20.08494023	15.36261101	11.90886848	8.998371074	7.94205476	11.12619629	24.66591249	16.94885706	18.38452226	16.52716582	18.14138717
c275291.graph_c0	3.611405903	2.248586835	2.821859694	38.8166298	94.20853209	74.54316206	75.27472013	66.57532402	42.20944017	24.00975703	34.14150715	38.00205692	40.14594681	21.30684127	21.48496224
c269049.graph_c0	9.877529318	16.16666994	13.22961125	33.75153081	63.97007093	51.49495741	26.38267543	16.7305033	26.88120376	38.88245826	67.12317852	84.04247153	73.44154675	52.56536735	55.44028203

c266780.graph_c1	3.938160335	3.02195192	6.613139707	14.62042405	12.28021688	25.44330562	49.74796731	44.84224072	25.9533897	16.86893652	20.88417542	22.037046	22.13489913	26.39433527	25.37260414
c270503.graph_c0	14.81773351	11.5596886	15.70689405	30.20378473	21.69345955	28.35908536	35.06717489	29.54596606	31.11958436	30.21114643	29.02323795	27.23400079	33.58674309	25.36893108	38.60652769
c263825.graph_c0	1.493183414	0.482454394	0.770960264	20.12629317	11.29177311	14.50403058	14.21972296	3.273533221	10.47553396	19.98112439	31.25268453	24.95727494	37.88229725	18.7950265	36.75828256
c273205.graph_c1	11.1650545	9.976349298	16.39023614	98.2242233	64.72042945	53.80701597	23.79325778	47.54959961	66.25276408	17.12331305	26.71601167	24.94329088	9.014011602	12.63292344	19.99817594
c269569.graph_c0	36.99973342	15.4039223	39.79936979	25.19851216	63.88248859	19.81497528	55.37874723	79.44965322	112.1264971	11.24230198	9.03493994	5.191370211	13.40984337	26.89356649	19.03622569
c276459.graph_c1	20.24064353	10.54895683	18.61251488	22.11817567	19.06433059	22.42524456	51.27645521	51.37538394	20.88318911	31.55064058	36.46769227	34.02376688	63.16665806	64.01043712	38.5492957
c274221.graph_c0	3.204902891	1.960286907	3.967651288	6.113106324	8.479318657	7.882376057	22.2252173	21.39494232	28.30181934	16.43435748	36.99568809	28.78705472	34.00189889	50.50491474	27.83222256
c269684.graph_c0	35.29324907	18.1875124	40.9384567	13.58750181	12.44333118	8.727575412	4.661839329	9.829269008	6.99849025	15.72821959	13.9542374	7.809603963	8.718891889	12.95074338	12.2908352
c261954.graph_c0	2.219203038	2.773381429	1.035945603	0.247572567	1.523322332	0.556483113	1.423782255	3.84471196	3.36862987	5.030530172	5.612064434	2.22613789	10.81379041	32.42890359	17.97689653
c270441.graph_c0	2.014195943	2.218495152	1.902383021	4.534233328	2.761909639	4.44350872	10.72193511	5.264524775	6.999087883	7.567811256	5.322155962	7.247819983	14.43145403	11.07559268	9.787174773
c256522.graph_c1	51.32291847	87.99317755	58.84820108	159.1315858	46.00271629	77.75178462	97.40729537	70.17426124	155.5240389	347.368128	371.1276257	484.3390572	256.1189505	402.7428195	575.7568011
c267819.graph_c1	17.62533212	6.351250431	18.02527519	65.42314925	107.5402739	68.96687223	32.03308862	25.04849545	18.66902797	82.9638306	45.50185343	38.47287682	63.62958296	16.13455334	17.68375868
c275725.	26.424658	16.62636617	25.11863652	42.70927613	57.80817964	53.81162072	53.85941316	38.30272298	27.95398956	52.77256323	82.10052941	67.6543689	82.78575579	85.04666619	64.85204583

graph_c0															
c267638.graph_c0	1.991481261	2.16933605	2.424807943	0.268959726	0.326947262	1.568290068	9.076500057	2.898285273	2.865923648	4.756798426	4.274231162	7.234005445	12.70050381	13.21582332	7.707511971
c275402.graph_c0	7.535822989	1.484719258	8.055796972	0.384165326	1.383677368	0.628584865	0.447387913	0.580655229	0.610971178	5.122697582	3.145336022	2.400272809	2.362058443	1.20516305	2.051121817

CO-like Family

GeneID	T01_FPKM	T04_FPKM	T012_FPKM	T02_FPKM	T03_FPKM	T05_FPKM	T06_FPKM	T09_FPKM	T013_FPKM	T07_FPKM	T10_FPKM	T14_FPKM	T08_FPKM	T11_FPKM	T15_FPKM
c262904.graph_c0	2.487701876	1.891265553	1.766118621	0.093793539	0	0.279033272	0.145639019	0.750527639	0.447503811	0.902761586	0.201424344	0.558118048	0.110725239	0.46458825	0
c267490.graph_c0	13.29696834	7.887982763	14.03535918	0.237084096	0	0.705318844	1.533896296	2.244932487	3.487760965	2.353241663	2.10022298	0.952268319	2.099120586	3.112028692	6.524783182
c274032.graph_c0	170.513912	227.0847782	242.685408	25.27309086	29.18663394	13.801407	21.9211772	97.48433151	96.30952705	38.1818614	50.49529265	30.83285404	34.3652195	67.28000331	68.02312931
c268944.graph_c0	19.15958233	13.65707879	8.230537564	42.60258407	102.4388698	87.33682388	28.11655024	17.46129166	28.0503125	24.52953702	7.644874494	4.511672769	14.19017182	3.73270359	1.507962287
c276691.graph_c1	17.70745796	11.15852309	12.02097367	1.394588728	2.084008938	2.577592209	3.071580154	5.033590935	3.633637315	10.53126322	5.543787532	3.354710405	4.763880999	3.380425943	4.666313442
c278011.graph_c0	10.67246525	16.53118241	12.41799993	36.47761609	25.70089965	28.4983963	45.34126421	30.72726126	41.52659619	41.7119913	63.72845946	58.3365492	53.30143727	51.03156878	39.17414876
c278425.graph_c3	0.244121189	0.296947476	0.392138818	42.92779773	23.03995068	33.17952685	13.08487532	20.13759681	18.03405269	10.8078683	17.05148144	16.0947241	7.214759356	7.489016874	12.15149118
c278192.graph_c1	9.807400148	19.43153889	17.37976139	40.00708189	38.95028277	25.78050515	7.409058362	67.5280183	63.01417369	22.22286661	33.39468921	57.90644874	10.49537752	63.68700631	43.31441737

c264745.graph_c0	2.495665081	8.400545863	14.3074756	108.3181584	51.1687636	46.19365833	13.7036614	38.00998847	128.3648161	44.50183368	67.0172651	116.7227328	17.28246517	76.95065749	56.46838145
c273047.graph_c0	7.497028835	6.135437386	10.89155136	125.2680932	81.74556044	78.98569452	71.84240396	41.19168601	47.54524585	27.4059952	71.37837842	56.63274575	41.88735809	74.05705654	82.04698922

Dof Family

GeneID	T01_FPKM	T04_FPKM	T012_FPKM	T02_FPKM	T03_FPKM	T05_FPKM	T06_FPKM	T09_FPKM	T013_FPKM	T07_FPKM	T10_FPKM	T14_FPKM	T08_FPKM	T11_FPKM	T15_FPKM
c271335.graph_c1	16.01669248	21.03865421	20.99101621	4.535012699	2.869889754	3.283256255	16.93970067	11.77481835	20.98158823	4.99831848	5.652747605	8.5297057	14.78813032	36.34972437	15.14670521
c262022.graph_c0	3.223800656	2.897807247	3.867543358	1.029571095	1.622374352	1.837767885	0.266446433	0.183078604	0.682256463	1.806439009	1.520085709	1.174237845	1.873788318	0.382483674	4.047827204
c270455.graph_c0	22.84455462	16.28048258	26.53203095	8.754918415	12.94177263	11.22856832	18.47818126	25.06451071	18.41022577	4.974033239	4.648134019	3.704611837	5.512194517	9.203187255	3.399559879
c277922.graph_c0	9.183595361	19.79338089	21.98950738	16.22172095	28.48082497	24.04203634	43.60266204	53.19641008	73.18378569	23.14096969	27.45660699	59.54404252	46.02559643	101.0006533	50.3780805

EIL Family

GeneID	T01_FPKM	T04_FPKM	T012_FPKM	T02_FPKM	T03_FPKM	T05_FPKM	T06_FPKM	T09_FPKM	T013_FPKM	T07_FPKM	T10_FPKM	T14_FPKM	T08_FPKM	T11_FPKM	T15_FPKM
c273785.graph_c0	7.914980753	11.98684602	11.99836694	14.67187	14.73447023	23.68679393	11.68555291	15.21516034	17.4250201	61.6871352	56.59943893	104.7094312	49.70297264	58.89123094	48.64046764
c276416.graph_c0	8.410678182	9.484131639	11.37683022	2.660269257	0.926065726	2.415063969	3.151304723	3.145537214	1.526597211	2.408723116	3.018469301	3.606610786	3.431890559	2.825611788	4.201181657
c272744.graph_c1	34.32563859	32.27263362	34.16513381	153.6822316	106.9644143	133.7696829	281.8224724	430.8966972	466.2061227	88.55400398	127.8198248	268.5401939	141.7256157	272.0561105	291.6208795

c272352.graph_c0	70.29946553	70.82447754	108.2574188	239.7737497	163.6947621	271.8939554	367.3166965	441.9090109	571.3054506	288.7000635	350.6902657	556.3925649	304.3442444	498.7729515	426.9024459
c277367.graph_c1	6.074197045	8.910669697	7.242597294	23.49508192	11.28408212	31.55186723	25.36841156	15.52834573	6.363684608	68.38508722	76.8806499	131.5741845	30.69522069	14.31678982	20.1131725
c275453.graph_c0	2.112643425	2.295638932	2.167704013	3.056230492	3.502998307	4.998900997	8.129908503	6.28767733	4.744397041	4.219053474	6.393376474	4.100926223	8.725187679	13.7874486	9.446663782

ERF Family

GeneID	T01_FPKM	T04_FPKM	T012_FPKM	T02_FPKM	T03_FPKM	T05_FPKM	T06_FPKM	T09_FPKM	T013_FPKM	T07_FPKM	T10_FPKM	T14_FPKM	T08_FPKM	T11_FPKM	T15_FPKM
c258655.graph_c0	20.486107	14.49863186	19.73934967	68.14869079	216.46457	71.60241146	86.23033867	157.6858557	109.5555854	62.79411009	150.598571	83.81646275	119.7860552	251.7514196	122.6925423
c271761.graph_c1	2.384167174	1.351176397	1.529415399	31.1509648	5.827744104	35.39672252	19.35477783	21.83159352	47.41065975	51.43947229	39.24004002	61.54151998	32.06663059	47.6339468	44.17010556
c274576.graph_c0	0.679396592	3.346975619	1.789008392	82.74738097	54.179832	38.73630745	16.60975646	6.919809818	8.897195804	17.34451671	105.6840786	69.46840121	24.13095724	21.22449044	65.74212301
c260047.graph_c0	1.08303656	0.35059808	0.21646192	21.62336892	4.82495915	7.599091666	9.293924854	3.608972194	8.647653152	42.02809385	33.26613676	43.90573483	21.19772703	25.05431517	46.91681596
c259122.graph_c0	0.385361982	0.301340499	1.250668873	2.889246884	7.670248818	2.904663301	5.569214893	6.005754916	2.772855662	13.96441478	17.2235063	12.3904216	7.762547928	10.11662878	8.936536373
c269493.graph_c0	4.120761728	1.392352388	7.801314233	2.175104517	2.145089186	1.561227653	0.964976874	1.510274673	0.988358826	16.49074388	13.08649437	11.01177966	11.57528317	16.55427767	11.78417132
c271309.graph_c0	44.8051811	71.66323256	59.31744929	74.94576192	170.9050468	200.6751384	513.042951	292.8446516	199.1711477	372.8314093	439.5204293	404.4416044	722.335256	568.3173101	341.4240847
c259843.	0	0	0	2.191052895	7.438442799	4.369038636	0.245202385	0	0.941789181	0.213738399	0.635858051	0.634273819	1.468061118	0.058664643	0.068983144

graph_c1

c266853.graph_c0	0.235262371	0.739276803	0.188954324	11.28272941	67.79366518	19.44987876	9.451411206	12.94629982	7.335554769	4.724045161	42.46597534	7.713109452	14.1572029	47.35735085	14.38328689
c273150.graph_c0	7.392167822	4.384539785	6.050037885	18.16162574	9.062501255	7.910486952	37.00066079	68.75863062	105.0955898	93.60660252	69.57764551	95.1071231	78.67103643	149.9061771	90.46451157
c267961.graph_c0	1.262520804	6.156128383	1.438597684	547.4143571	117.5357641	231.1556102	50.2808968	9.792978502	75.87052344	270.2013896	215.5583418	292.874137	192.7169121	95.33999793	640.2575261
c265780.graph_c0	0.774423528	0.519487444	0.564494476	84.62611418	62.85753317	47.80137083	89.28828416	129.7388273	191.3276366	6.025622046	8.254738559	9.001909795	9.975691608	3.654801367	3.841468566
c273737.graph_c1	2.164560507	2.197221792	1.57409239	48.93851985	27.83336772	74.45319209	25.61552244	22.25110135	29.2634555	127.6487756	91.91534606	117.3698092	103.8545282	71.00054041	77.57581139
c260032.graph_c0	2.783976442	3.691582194	3.275985823	84.88898067	121.9760045	109.2782019	37.8122875	111.3840833	32.09775949	62.90733934	110.1798334	77.21644551	150.9422742	171.1233834	80.70044492
c265315.graph_c0	57.39005456	39.42295996	41.66727377	186.752425	171.1034472	166.6505768	226.1374529	260.1395085	116.1354054	391.2963191	486.8833397	356.1347146	613.632862	919.3396098	446.1153842
c267545.graph_c2	0.446731103	0.724534532	0.615083052	3.997417608	1.213297915	8.765494472	1.813291734	0.335444022	0.785750668	6.916870477	7.041276632	16.83771701	10.60457367	5.339444217	5.755380568
c273976.graph_c0	23.08207097	27.45861228	24.30887965	2.59725468	3.191326821	9.315042327	23.31997305	17.47311744	9.609469475	17.14309753	11.98813793	12.14931003	44.56510943	26.1230999	12.33337661
c264426.graph_c0	2.018562764	0.598495712	0.729556843	3.516080619	4.378994391	3.749386812	0.803582704	0.58463101	0	4.230278728	5.001235526	3.695388898	3.180488387	3.799904468	1.702197404
c269779.graph_c0	22.11327452	25.8905434	20.48202017	0.515470363	1.993744535	2.063268733	5.020707554	4.749710311	6.893746464	7.047436758	4.453108968	3.346149984	6.804399909	5.152989851	4.148742248
c262844.graph_c0	2.180111206	2.757345775	1.432626022	6.545829054	3.633381454	8.749811749	11.55645709	5.166162482	1.497385618	43.42280188	40.9685318	22.78362461	65.89536357	15.9452578	4.282698512

c264629.graph_c0	0.029588054	0.18895129	0.0611076	36.25107528	3.97779919	6.212693061	8.148227001	2.085247412	2.469635057	50.8943386	30.47313465	57.28950291	23.76806206	11.56415785	28.53745038
c256220.graph_c0	0	0.128081356	0.144977072	38.74682872	24.53693441	26.07765171	12.5589478	10.50438251	65.86527094	2.980444877	2.932809268	1.436293075	0.974817649	2.391196232	4.88362277
c277205.graph_c0	0.553873929	0.918721032	0.7279392	42.60059667	193.848554	56.49562892	71.31311753	311.9868475	35.57853998	54.10425021	61.15375382	26.0814832	139.8463457	196.9763592	67.72462234
c273921.graph_c0	0.684538814	1.38476425	0.76834998	38.21540534	9.566647336	35.22060607	9.645966162	3.333086359	5.696528506	34.99371009	50.08561366	35.09565337	23.62210481	18.67580833	109.1759967

FAR1 Family

GeneID	T01_FPKM	T04_FPKM	T012_FPKM	T02_FPKM	T03_FPKM	T05_FPKM	T06_FPKM	T09_FPKM	T013_FPKM	T07_FPKM	T10_FPKM	T14_FPKM	T08_FPKM	T11_FPKM	T15_FPKM
c272244.graph_c0	0.247066746	0	0.637827348	2.235631023	1.962733908	2.460848729	0.867850575	0.178893225	0.622215371	0.537947982	1.38031119	2.32804472	0.725782554	0.775163768	1.106829955
c275251.graph_c1	7.526023719	4.140274895	6.892964619	62.76212948	12.50298038	52.3292517	33.97273462	7.430110194	20.98095637	50.90994396	37.27858567	61.74199785	53.81164487	12.20467258	46.4824206
c274949.graph_c0	13.82414185	13.44937671	14.81968306	3.215731314	3.096475714	5.456231523	16.67607005	20.89750194	10.22850848	10.08881434	6.790227531	4.467930746	13.29591571	14.67861396	7.617531261
c277736.graph_c0	0.911561119	0.981116344	0.856701719	202.8003186	75.47008588	160.1478831	49.99381201	36.23010446	25.76473065	221.7486845	108.2330395	168.2805425	140.9195299	20.11635494	99.51995468
c278580.graph_c2	0.388677411	0.225647269	0.291900902	0	0.259108488	1.331660602	3.574536738	0.409351322	0.355945124	1.115554021	2.609186231	2.016700735	4.2274081	1.330322795	1.48982167
c278805.graph_c0	0.133679653	0	0.034510731	0.030240652	0.367605163	0.323874473	0.344347867	0	0.192377246	0.945964109	0.19482803	1.187649334	2.39188149	1.408037764	0.739779501
c278277.graph_c1	17.58928471	24.34168206	20.54349487	160.8214447	130.7975279	155.5572491	98.21467001	103.2727848	152.9436954	48.87704725	46.48756244	58.88625075	43.69187425	27.64761781	41.49778758

c278656.graph_c4	0.944935757	0.492605957	0.75672596	2.547680082	16.05044541	8.119174041	7.731243739	4.300673945	4.051794875	4.261850714	11.09232994	9.137509684	7.827973305	7.796395657	9.127045665
c274453.graph_c0	4.374908162	1.072905387	4.007640659	57.27373782	52.34795449	57.44178634	63.43034223	54.6802906	48.70854788	28.26969103	28.0639781	35.76511814	45.67824523	19.65092666	13.5619145
c270327.graph_c0	2.674670689	1.040038117	5.705055344	199.3711814	51.92757413	111.0941783	34.21250533	1.269930849	8.076791001	361.7354539	357.3499903	707.423116	609.2710573	185.2853949	207.1524218

GATA Family

GeneID	T01_FPKM	T04_FPKM	T012_FPKM	T02_FPKM	T03_FPKM	T05_FPKM	T06_FPKM	T09_FPKM	T013_FPKM	T07_FPKM	T10_FPKM	T14_FPKM	T08_FPKM	T11_FPKM	T15_FPKM
c276841.graph_c0	5.700706756	4.339391354	4.924158814	20.24426435	13.27724532	10.25646103	8.127300848	8.111178937	10.80086053	15.15766274	9.695979026	10.55341813	7.621565562	5.388767341	5.139823017
c256376.graph_c0	0	0	0	0.270672093	0.243725048	0.26841408	1.167470191	0.14439295	0.07174552	4.287750261	8.961340856	4.348709412	7.615562251	0.044690738	0.262756872
c273790.graph_c0	2.241067172	1.600055209	2.394709451	0.052901029	0.33344088	0.041967773	0.109523592	0.39508875	0.056088706	0.551602688	0.359753894	0.230844052	0.395521568	0.174690046	0.267041074
c265601.graph_c0	7.315057095	5.683468473	9.712052534	22.47608367	25.24894435	28.21777839	4.4048547	6.635303272	23.94611508	15.22725316	6.873119098	9.608780606	5.538543599	9.836035901	7.456528453
c271924.graph_c1	16.22474415	13.20454451	18.82717505	29.66299829	52.11006549	35.59118093	33.95797538	51.49372068	41.46328561	34.70238301	59.37615068	50.5961515	50.52751287	37.19249033	38.34105994
c256083.graph_c0	2.994082303	3.511912029	5.123568145	0.696665064	1.045513064	0.552682649	0.400649945	0.330349776	0.61553689	4.190870062	2.576631152	2.947912842	1.827617138	3.450796348	2.885515076
c276374.graph_c0	22.63756137	11.15313839	37.13165757	76.9004074	31.68221386	81.92272635	51.52318576	78.6844189	48.00081991	113.8234939	149.6568884	141.3191126	103.8802356	83.31128729	185.1057425
c270767.graph_c0	10.58392642	9.810294151	10.30706937	21.79550187	17.44670733	27.90344471	20.23360686	19.94506513	19.73014457	19.62087749	18.1699869	23.14009361	31.92618011	23.01004554	18.58078356

c263742.graph_c0	21.26509084	8.369183547	18.41837687	1.905161082	6.910977063	2.720545571	2.310261145	4.58799832	3.5060753	17.45303781	9.643987477	7.644142986	9.992352673	7.765178477	7.767684761
c268641.graph_c0	25.72522077	16.5617339	25.65865837	36.1080886	34.42709447	65.64746507	53.5286372	45.33564391	47.24813605	64.79842827	84.49831039	89.94699538	110.4167821	62.17872924	81.58588866
c275551.graph_c0	18.65469432	21.2353937	17.40330929	5.83402306	2.40450271	5.462054993	4.37400247	5.652345401	9.039567882	7.406129357	7.346812514	3.77784461	4.760261272	6.629474806	8.495129447

GRAS Family

GeneID	T01_FPKM	T04_FPKM	T012_FPKM	T02_FPKM	T03_FPKM	T05_FPKM	T06_FPKM	T09_FPKM	T013_FPKM	T07_FPKM	T10_FPKM	T14_FPKM	T08_FPKM	T11_FPKM	T15_FPKM
c273415.graph_c0	17.29623224	14.26352815	16.49702283	2.82691489	3.852928663	5.825833734	6.743906994	5.552359501	6.437291347	10.58987377	6.001894347	4.771201787	10.60329714	5.588301974	6.421539988
c271286.graph_c0	32.91504731	53.28478759	33.46241859	22.12272047	48.61944383	24.55243085	17.51243447	33.77005474	25.33624535	8.030363423	5.093489285	6.569567927	4.261868755	3.971518423	5.159079531
c277288.graph_c0	54.02005353	29.72202535	45.0586593	194.5988832	82.79456308	130.1320589	84.20925618	99.89720907	78.91726723	221.807746	185.8085999	204.9183349	170.5331088	228.4541435	199.8066232
c277458.graph_c0	14.15582019	11.37018822	14.66119603	26.44105611	20.30975757	31.64531827	79.09974693	41.72836219	31.42286792	50.14668718	50.2728754	56.20160842	101.4873093	71.62322128	66.24736715

GRF Family

GeneID	T01_FPKM	T04_FPKM	T012_FPKM	T02_FPKM	T03_FPKM	T05_FPKM	T06_FPKM	T09_FPKM	T013_FPKM	T07_FPKM	T10_FPKM	T14_FPKM	T08_FPKM	T11_FPKM	T15_FPKM
c267010.graph_c0	40.16564122	31.43594242	35.98546723	11.00283873	12.82479051	14.50565292	8.235232575	10.57551703	16.37639176	25.93522738	23.25002569	27.63963679	20.07059284	16.23890798	27.72722326
c258669.graph_c0	49.86926719	19.59004206	39.55968002	0.334485737	3.65529865	1.121733448	1.353527034	2.6278613	2.127845807	18.9506817	12.37465685	6.369138956	8.758877413	6.476623799	11.0517578

c201309. graph_c0	79.78504495	72.32508282	98.40642363	258.7829628	293.0297271	246.0535096	243.7653784	203.4789959	273.6957084	268.1939813	267.7263293	333.7422359	358.3464159	353.7873428	448.0735453
c267426. graph_c0	2.220188798	1.575363803	2.037915713	0	0	0	0	0.714474918	0.08875152	2.349911154	1.857564787	6.707763637	0.527031933	1.658516464	1.820216794
c266903. graph_c0	6.822216113	2.503857966	4.210739721	0.402086711	0.990331135	0.450333068	1.003848595	0.681344986	0.677089162	4.296926299	2.793653378	1.604459621	2.149983995	1.359014483	2.782812969

HB-PHD Family

GeneID	T01_FPKM	T04_FPKM	T012_FPKM	T02_FPKM	T03_FPKM	T05_FPKM	T06_FPKM	T09_FPKM	T013_FPKM	T07_FPKM	T10_FPKM	T14_FPKM	T08_FPKM	T11_FPKM	T15_FPKM
c276207. graph_c0	3.797670479	2.535683186	7.843250892	0.971155182	0.672669849	1.185296697	6.598991223	7.917221423	5.029563609	1.468014226	2.032107384	2.015190354	4.350703066	7.672019662	4.554235916
c276838. graph_c0	12.85851719	10.87680076	14.60094996	15.66973113	23.81390651	42.06805765	46.03230055	40.04799784	32.61343973	41.27195095	49.97418851	63.00180201	55.54796542	64.08094696	65.84871668
c276184. graph_c0	4.276762464	3.984432883	6.423788899	3.553281798	4.023172744	13.84336764	20.2729549	10.76014134	9.45578396	12.47238324	12.22686985	14.37498451	17.42956131	12.58174192	12.02157974
c268566. graph_c0	19.88297761	8.393380212	15.5022318	12.56210904	20.27438108	16.21562076	84.41563303	116.6042322	54.11153842	35.76070933	34.13338754	26.58086889	86.03816709	66.14726535	41.91689439
c276459. graph_c1	20.24064353	10.54895683	18.61251488	22.11817567	19.06433059	22.42524456	51.27645521	51.37538394	20.88318911	31.55064058	36.46769227	34.02376688	63.16665806	64.01043712	38.5492957

HB-other Family

GeneID	T01_FPKM	T04_FPKM	T012_FPKM	T02_FPKM	T03_FPKM	T05_FPKM	T06_FPKM	T09_FPKM	T013_FPKM	T07_FPKM	T10_FPKM	T14_FPKM	T08_FPKM	T11_FPKM	T15_FPKM
c276901. graph_c1	66.46671183	48.57864978	57.74175888	20.15355966	22.37929028	23.8374356	21.70805529	22.00958087	19.13809136	42.48030521	30.30672049	22.53375174	35.56520862	29.01196921	27.46801197

c273560.graph_c0	22.45044563	11.92668954	23.67798617	0.371610696	0.920190152	0.368510628	2.564546079	0.92511877	2.659520108	6.929949387	4.854772149	4.348826731	5.264330597	4.11090131	6.349089859
c269000.graph_c0	63.70767631	49.29233099	53.80523916	12.83688393	11.66592121	9.853804647	16.77457295	8.665646471	16.25686762	33.74877558	25.35536698	23.95308493	24.13448352	22.09776202	23.07684617
c271722.graph_c0	16.33064129	4.900778572	13.40217915	2.916531954	5.357394017	7.357760676	2.495805431	2.738305591	2.535665089	4.912526812	2.881134964	1.943738799	3.167584942	4.160586199	3.170993575
c276249.graph_c1	13.33894814	6.150954665	14.16530227	1.712991845	3.256949119	2.806777793	1.500438463	3.19131962	4.505603413	5.04137754	3.579657709	3.293171708	3.951112206	2.72824597	4.297954573
c277865.graph_c0	5.051992377	4.769019742	6.912700336	11.74042512	8.64112959	10.16439437	10.55642157	11.06170751	13.58441712	14.58919528	13.5077824	14.70125771	16.60499931	19.31722076	23.05848401
c267038.graph_c0	4.142116679	3.483612104	3.375068299	0.029281828	0	0.034845063	0	0	0	0	0.157208938	0.104544836	0.518517184	0.174050284	0.238774574
c269200.graph_c2	25.72817006	21.14391724	28.01709063	5.002449443	3.274370707	5.311783955	7.090435628	7.923720846	13.89209588	11.43527118	10.86686173	12.77693534	11.72013908	11.47443063	11.56514037

HD-ZIP Family

GeneID	T01_FPKM	T04_FPKM	T012_FPKM	T02_FPKM	T03_FPKM	T05_FPKM	T06_FPKM	T09_FPKM	T013_FPKM	T07_FPKM	T10_FPKM	T14_FPKM	T08_FPKM	T11_FPKM	T15_FPKM
c269903.graph_c0	30.80936216	15.51933247	26.34982315	48.5164752	44.19971963	43.96203064	40.83939839	44.34245269	67.54893604	66.19072736	51.60553589	54.34696988	46.64086746	44.22285085	50.07522086
c276850.graph_c0	34.45211573	47.0120067	44.66001704	45.72283516	25.62228435	47.12173376	98.91927861	175.221347	104.6449087	45.0083545	48.38333873	46.52089016	62.48567522	190.4046675	167.9275918
c269903.graph_c1	25.28925637	12.4523737	22.13695666	36.67417143	33.21066648	39.58425968	42.55281075	46.12146564	55.31337952	65.2281027	48.73610396	50.18376671	39.89546315	39.18417841	49.29807634
c261527.graph_c0	18.89230945	5.396509796	23.34444682	1.24478665	6.276819372	2.221924199	0.987904728	2.03640428	2.441619308	4.892264198	5.034555671	3.506026174	7.347483677	9.207611335	9.473742697

c276080.graph_c1	122.6756217	89.43120862	149.1219924	214.188564	415.3972932	370.5046531	291.8694703	336.7587517	305.1385265	400.50095	501.4060876	352.757167	489.4184414	383.6499778	310.3774559
c277556.graph_c0	36.98231776	19.30180988	35.39651311	4.703728215	9.963451249	7.229096649	5.821454396	9.962944401	10.46199958	19.96369749	14.51842226	9.563944736	14.95786098	12.4318717	15.18464743
c266322.graph_c1	14.45843643	12.18126439	11.91165014	25.30808794	60.25437744	21.94920711	33.74694595	18.99275307	45.36610855	25.11591956	37.00088806	32.58652009	64.31092373	83.00590283	36.89365188
c277530.graph_c1	51.14194695	28.21651046	60.94336712	68.59078832	46.19895123	87.42515311	88.99656358	68.29132765	77.79782078	129.1053208	136.5584661	147.4458687	178.4899889	82.99648109	81.09019259
c269574.graph_c1	0.996464632	0.763618778	1.934499275	2.488609781	26.01338755	4.635264553	0.522696159	0.192402282	0.229440597	5.510888106	5.692906937	4.464000043	13.32679613	16.04276963	10.2515465
c276170.graph_c0	14.07749527	11.01560015	16.16205004	25.68371206	35.91406008	22.2446909	21.87005319	24.11531093	51.34700688	27.72338691	65.27595726	57.15749303	40.74271566	55.53020976	41.65159581
c274433.graph_c0	31.65513108	11.90582147	45.97818745	0.040861277	0.827849005	0.729367232	1.903434801	2.790129797	2.729378591	5.604371832	3.071277144	2.33419055	7.718014982	7.650662226	6.997148845
c252474.graph_c1	0.967637146	1.000473568	0.466302932	3.268851057	2.128721325	1.597636594	0.120851115	0.06227871	0.185669112	2.458019417	2.131056967	0.4862821	1.240375885	0.231308925	1.767959452
c278766.graph_c0	24.76899582	16.35383788	22.73069368	3.86207591	3.83104211	4.505419271	6.186948846	7.714176593	9.283771672	12.38573178	7.043033639	5.364806992	6.861314956	9.069525301	10.84176905

HRT-like Family

GeneID	T01_FPKM	T04_FPKM	T012_FPKM	T02_FPKM	T03_FPKM	T05_FPKM	T06_FPKM	T09_FPKM	T013_FPKM	T07_FPKM	T10_FPKM	T14_FPKM	T08_FPKM	T11_FPKM	T15_FPKM
c277380.graph_c0	5.514613445	5.206863326	6.291945586	6.944131671	8.986438072	21.93895514	30.30682155	8.724317113	9.952414395	18.9763842	21.39488791	38.04026049	31.47255004	16.97350982	17.09994909
c270635.graph_c2	16.55337591	20.7296378	27.03855983	75.85305019	55.88297979	62.82132047	63.98309541	18.24479079	50.69669303	17.92998341	17.90072782	15.27089768	22.54307812	21.36982083	21.65645553

c274803. graph_c0	7.571081429	7.408092193	7.41311148	0.5501969	0.311636084	0.950412231	3.637769655	2.878276655	3.641235051	4.057141973	1.962920482	1.372944368	4.106636096	5.06377297	2.336289921

LBD Family

GeneID	T01_FPKM	T04_FPKM	T012_FPKM	T02_FPKM	T03_FPKM	T05_FPKM	T06_FPKM	T09_FPKM	T013_FPKM	T07_FPKM	T10_FPKM	T14_FPKM	T08_FPKM	T11_FPKM	T15_FPKM
c264398. graph_c0	8.09344923	4.767705995	7.736765718	0.125545944	0.678282509	0.348596104	0.996375012	2.232461291	4.126441376	1.409774405	0.988583024	0.597648474	1.630305802	1.326651042	1.852494273
c272398. graph_c2	129.2569125	106.064625	126.9464612	8.753589104	69.83507564	11.70307431	210.7069855	530.629438	199.0115884	119.7499079	85.95257203	62.34894053	380.2961225	552.9345266	125.6519981
c277690. graph_c1	25.19549947	28.34782337	24.87189909	3.198503143	20.84703849	4.425795962	3.503509369	8.610741792	4.613659	11.90255306	13.05885561	6.108162112	12.7765919	14.8483662	11.69779325
c263619. graph_c0	1.576056923	1.132835192	3.797497683	13.13764056	7.588135638	13.01090095	5.457794546	5.94793213	3.299040054	3.379615613	3.062645739	2.108680522	4.540539959	2.825615216	2.164731785
c271451. graph_c0	11.74425469	3.646035811	12.48960151	0	0.342771271	0.26424549	0.032838253	0.304608135	0.201803659	2.175461999	1.29437203	1.170338032	1.160918906	0.879933417	2.291131533
c262170. graph_c0	1.033198002	1.060404361	1.111376368	0.038954546	0.052614578	0.092710986	0	0	0.061952767	0.140601373	0.334624066	0.834475887	0.183946633	0.231544619	0.499163338

LSD Fmaily

GeneID	T01_FPKM	T04_FPKM	T012_FPKM	T02_FPKM	T03_FPKM	T05_FPKM	T06_FPKM	T09_FPKM	T013_FPKM	T07_FPKM	T10_FPKM	T14_FPKM	T08_FPKM	T11_FPKM	T15_FPKM
c275350. graph_c0	13.42550156	5.508532334	15.36154403	27.04375556	32.77542634	36.75842123	29.98562137	35.26908873	20.92247749	26.4041584	28.02260024	17.40010749	34.73633683	67.46183396	63.75370317

M-type Family

GeneID	T01_FPKM	T04_FPKM	T012_FPKM	T02_FPKM	T03_FPKM	T05_FPKM	T06_FPKM	T09_FPKM	T013_FPKM	T07_FPKM	T10_FPKM	T14_FPKM	T08_FPKM	T11_FPKM	T15_FPKM
c274605.graph_c0	9.566299509	7.612946138	9.701717486	8.976469604	9.194310027	12.42739211	23.83468502	19.39683886	22.112274	15.88279027	18.65609808	14.67821527	24.4009413	24.11963125	19.74632205
c256393.graph_c0	4.158997468	1.157562834	6.041761397	0.287035089	0.086153003	0.151808305	0.396175202	0.476379451	0.253609224	0.383709579	0.308208085	0.113866737	0.301200835	0.631899149	1.523239471
c266100.graph_c0	16.15677491	9.101965058	16.79945587	1.246385827	1.00097078	0.841808158	4.70758431	1.365736375	3.535878004	0.607928069	0.614906007	0.561257234	1.193014538	0.567315948	0.706342161
c264894.graph_c0	0	0	0	0	0.153833136	0.203299481	3.595964138	2.187292981	4.256693435	0	0	0	0	0	0
c266248.graph_c0	0	0.034299933	0.038824572	0.544331738	0.229753227	0.728717564	2.852609036	1.923765118	6.709156438	0	0.109590767	0.040488045	0.321297514	0.370733347	0.118893134
c265199.graph_c0	0	0	0.110787597	0.58247782	0.524488626	0.423587007	5.728178704	2.244154466	6.690411018	0.155731705	0.312721999	0.231068782	0.114604546	0.19234612	0.075392602
c271790.graph_c0	3.698275029	1.816725523	2.408898707	53.74922171	52.50087892	36.51413347	42.7957643	47.67864544	55.92357726	29.42222078	28.91228437	40.74520099	26.55992362	33.66207707	31.48638716

MYB Family

GeneID	T01_FPKM	T04_FPKM	T012_FPKM	T02_FPKM	T03_FPKM	T05_FPKM	T06_FPKM	T09_FPKM	T013_FPKM	T07_FPKM	T10_FPKM	T14_FPKM	T08_FPKM	T11_FPKM	T15_FPKM
c260427.graph_c0	32.78025528	13.89466645	26.74484683	2.728330102	1.511820505	2.247704057	3.620894509	1.343498898	1.668883903	8.837547597	4.356817193	6.410710582	9.910310398	9.771848234	7.334434991
c277171.graph_c0	3.900803257	3.086292643	5.311420736	11.86984458	6.848840993	17.56950273	16.8142825	34.35995633	21.31185669	17.21222732	19.33055001	20.40877408	16.34803375	16.48322737	21.08054331
c269840.graph_c0	1.959193271	0.696375102	2.469808279	0.230235553	0.031097131	0.082193354	0.023833397	0.122821641	0	0.858705493	0.173053132	0.164401879	0.298977686	0.205276952	0.214562698

c278667. graph_c1	44.39377466	42.45280802	63.73785625	11.495153	5.231624485	10.9284187	43.65301718	16.86358695	41.3326093	53.59158752	28.37568388	25.57634689	35.84321076	58.48628481	54.50929905
c275312. graph_c0	97.89420299	45.62761775	106.0843367	26.34701105	34.67800634	29.39524762	10.36461866	27.85076306	18.63065778	52.21165544	36.56875373	30.04481784	29.38087189	27.68799961	32.95792503
c276372. graph_c0	18.56321847	14.45163183	18.23207921	39.36570585	82.67003218	51.95358457	48.23018092	28.90949949	44.84697074	31.04766408	46.40072315	42.49363438	47.05363387	50.4790532	68.75884452
c270915. graph_c1	67.96556985	26.15194987	34.86488481	6.203840113	15.0195276	6.741757799	37.90225348	97.11213024	8.153825192	26.64501801	16.38971323	13.26187143	92.38996501	116.0068684	32.69834353
c275853. graph_c0	11.48872648	10.45235611	13.5724811	3.893075638	3.871454799	4.463021723	4.620490615	5.188204375	5.987378045	6.176505813	4.976483772	4.785887006	6.919038065	5.015149003	7.72458211
c278825. graph_c0	4.098550205	0.714825643	2.365123245	75.04566552	128.5804596	115.8479779	41.24211796	52.54452032	31.74613245	100.525468	76.30634148	66.49703482	80.31958154	21.39575708	32.56063723
c269643. graph_c0	12.74093904	11.46562413	11.21343561	20.49361899	19.27291675	27.32203515	28.58989433	31.55505506	17.52600495	26.25771937	25.57829494	13.65063109	26.1803585	31.37442581	12.90566102
c277379. graph_c1	7.338775051	11.20912665	7.728588858	24.0981572	47.99705627	25.76636084	64.14633655	73.68033466	57.35342728	27.65770043	37.00574989	33.71652542	95.80508213	101.810178	61.68860494
c274996. graph_c0	8.937006696	6.90125976	11.70859146	11.85860358	17.35875089	28.33404701	22.06095407	49.58205136	44.73021084	27.63834715	45.8000506	35.00519853	36.75740344	34.582385	60.66309498
c274509. graph_c0	2.737895185	1.672817661	2.61975409	35.02505223	22.71725294	16.49867416	3.717464245	5.650209823	5.530573173	11.64919489	9.518089769	8.655840976	5.071210763	2.229136384	3.627342518
c276896. graph_c1	20.05613136	10.57126558	20.50691217	1.000293202	0.796161497	0.658937224	5.140408659	3.220760986	4.488476299	3.696390933	9.455733991	5.293242309	11.59780181	17.4478331	11.29876475
c274202. graph_c0	5.783273477	3.204080805	6.576495292	9.576372788	7.783581088	17.34579083	15.28653122	9.968589228	8.929168985	17.30782688	21.77126096	27.18094225	28.76299812	18.21829069	22.18779514
c267575.	3.774407366	1.138305455	3.865391435	0.028225993	0.228743323	0	0.146094141	0.090344765	0.044890226	0.882943148	0.727393661	0.30232557	0.799713042	0.587210512	0.756256144

graph_c0															
c278838. graph_c0	26.90619798	20.73321478	26.98058354	52.02361264	65.89231326	66.31714531	32.9144451	24.5464123	31.08596976	41.90223057	53.58391472	52.25497044	45.42628633	54.20759274	77.94075926
c277424. graph_c0	12.17852303	12.63683907	13.01505479	28.89019445	25.29325193	27.40870512	35.53311039	30.6646877	35.84147649	28.95045923	29.62210986	25.52080475	33.16787247	41.91020482	37.5576562
c269974. graph_c2	4.01029641	3.530203427	8.427687826	0.127326103	0.429937569	0.454550101	0.263609467	0	0.404995317	1.685079094	0.751950361	0.37882671	0.601244534	0.693753886	1.112424528
c276789. graph_c0	3.336029751	2.173888696	5.554049674	0	0.748876355	0.476514458	0.22320364	0.920195713	0.930777405	1.408261894	0.69457317	0.989775215	1.309079062	0.976483577	1.327648909
c266819. graph_c0	2.883055841	1.951437387	2.713055299	11.5291625	4.717079883	6.359075425	8.733234764	9.225534255	13.8187835	7.644239828	9.939829044	10.99169983	7.798670176	11.88002489	12.3275574
c270175. graph_c0	0	0	0.028372706	4.599487929	1.64543896	1.272182064	0.437522344	1.061036315	1.304830964	0.089736509	0.213568294	0	0	0.049259845	0.02896207
c261125. graph_c0	24.30055879	25.66838289	23.96210043	3.470615555	8.404272699	4.926494498	4.208582855	7.114809155	11.63062487	13.86888622	10.94030253	10.8569898	5.706722939	4.641577119	13.31285633
c278730. graph_c0	7.950284337	6.834008422	7.392072406	4.585796254	7.045537027	17.95710019	24.81842848	15.10616498	10.14208736	17.96563843	14.52599224	20.03944924	18.10182067	16.49666663	16.56030364
c275164. graph_c1	8.495310823	4.592730021	6.213921839	0	0.048068359	0.254100584	0.257883143	0.113910855	0.226398688	0.813532889	0.573207218	1.058850148	1.680525279	2.009608707	2.072875439
c274804. graph_c0	18.39806045	17.9309947	18.99856832	22.4084018	17.8273838	30.19309256	27.67382098	20.9244792	27.81069313	37.14299391	43.31843006	34.63318689	45.27040822	52.80145182	45.97472287
c270609. graph_c0	11.99207535	6.98403787	12.97753566	0.920955671	2.56893101	1.453295027	2.922224434	4.293480099	3.152235448	6.744489537	4.299519541	3.669312949	4.254290122	7.259175118	5.364145845
c265658. graph_c0	37.67148437	31.52671608	40.2991336	9.188077637	11.87225563	11.55329124	14.90097959	23.4290705	22.55209808	23.06682888	16.57459688	8.784227642	20.24720742	20.44977769	16.12626094

c273403.graph_c0	20.22307778	21.28315246	17.21495271	2.421958414	7.511774605	4.145249217	6.654779701	6.157032896	5.635112261	6.871082915	6.790489332	6.921247927	5.894837499	4.798681996	3.848892554
c264841.graph_c0	6.584217299	4.944331898	7.169144799	0	0	0	0.167820962	0.172967754	0	0.292572877	0.174077115	0.289405674	0.334922556	0.883325214	0.755413049
c278482.graph_c0	51.82649786	32.40415943	47.57479006	98.87634458	98.61248735	89.41942087	66.67018752	61.67667349	71.94051786	76.89015641	75.97259746	71.54213888	75.79698319	57.7104521	62.4588096
c276801.graph_c0	7.436499046	6.949753247	7.991388017	14.88052151	9.433540283	15.01679131	17.00851293	10.33130104	19.34444999	17.61788203	17.09429812	22.80939926	24.52027976	14.09119708	18.86395734
c277256.graph_c0	9.655531832	10.17829691	11.66782387	39.46661732	69.22082396	61.97318573	35.27430171	61.13257185	32.17953924	62.82923192	67.86598058	60.396248	65.98727534	51.4507791	44.90891441
c260478.graph_c0	30.37068151	11.13752232	31.53890896	3.643526972	8.742961126	20.47953837	69.09408066	26.01221187	19.04825401	48.59280993	76.24803241	49.40452679	165.2782022	122.1470823	67.36817847
c269480.graph_c0	22.94733344	26.51423385	18.08450303	213.7478029	262.2802167	210.2953639	84.30331153	108.022969	77.39761968	307.0178128	310.8530179	363.1892492	283.998319	168.7019531	167.4702702
c265373.graph_c0	7.654862188	8.824105572	7.169392566	0.590644527	0.580191587	0.511171396	0.889338394	1.203054483	0.512374265	0.969024856	1.72967214	1.086339468	1.521312537	4.574643374	2.689637809
c270562.graph_c0	48.46507699	30.00266974	40.50229331	9.898600271	13.87741222	7.231551638	4.312726382	7.28577407	11.70731019	6.783742718	5.987089399	5.070009235	6.508382601	6.051271081	6.239857346
c262866.graph_c1	7.133191476	5.189586617	9.790275723	0	0	0.09722673	0	0	0.129940694	0.589799002	0	0.097235704	0.289359391	0.404704395	0.76142031
c273967.graph_c1	12.48827212	8.422426331	11.53113737	1.542519267	2.78570737	1.959328491	1.381480873	2.034069453	2.783969824	5.213036414	4.224501654	2.928950776	2.557551026	4.309626225	4.017774089
c269279.graph_c0	21.02472363	18.75687188	35.58519139	0.035036144	0.09464425	0.125077909	1.124324186	1.270947812	1.615908836	0.800903255	1.053375873	1.459376945	0.78585714	4.824545341	3.061042848
c257995.	2.251583819	1.81935393	2.1922135	0	0.078623767	0	0.06025866	0.186320081	0.27773443	0.490246054	0	0	0.412316639	0.230670041	0.271242504

graph_c1															
c270957.graph_c2	17.01451807	12.36717429	19.11301769	0.907914646	1.069741789	3.011351955	5.619103662	1.937347468	2.24270723	4.043957393	2.426845128	2.827713551	3.466286496	1.24390099	2.047766352
c275047.graph_c0	42.50150081	42.54753567	50.68028068	5.614350573	16.58173817	8.217651067	51.57032689	46.02371848	47.41290961	13.10422727	13.96600245	19.48809839	29.1625776	46.40496137	19.68601763
c263169.graph_c0	25.99716268	14.39966096	24.32891856	3.500600187	6.666679322	5.790290722	2.065527244	4.29509579	3.39605649	5.980548479	5.450289242	5.707503897	5.372291475	5.513976731	8.278094809
c272415.graph_c1	4.849841369	1.709461025	3.94580642	8.544222685	39.3361166	28.2871071	8.844761346	3.972738325	2.273581098	48.91882889	102.6685323	44.15569937	50.66865558	31.15701654	40.27769614
c260150.graph_c0	0.10025974	0.045733243	0	11.56704943	15.99082459	11.01173207	3.146087332	0.725949101	2.164244012	7.367605077	8.962089372	9.879083097	10.01377251	4.04436379	8.877354013
c273261.graph_c1	29.15733649	16.84345832	20.42080688	0.876207367	1.656848798	1.621942613	2.559833202	3.34466607	2.570248047	6.723361181	5.645042127	4.333303732	5.746562025	4.282256271	5.965433125

MYB_related Family

GeneID	T01_FPKM	T04_FPKM	T012_FPKM	T02_FPKM	T03_FPKM	T05_FPKM	T06_FPKM	T09_FPKM	T013_FPKM	T07_FPKM	T10_FPKM	T14_FPKM	T08_FPKM	T11_FPKM	T15_FPKM
c277133.graph_c0	5.282184599	4.754873346	19.04577408	53.735744	39.0991685	43.95271226	8.58058653	23.02421704	18.73869646	55.1747355	68.91888739	76.25346601	35.26106383	25.57871637	27.74699893
c278219.graph_c0	10.86268939	17.93015019	19.19974909	5.85187046	3.457965868	1.795319432	1.751055932	3.316852423	6.034834794	1.320097884	1.423612421	1.196990084	2.968391143	3.577973491	4.633357741
c272831.graph_c2	28.66764438	7.944698853	36.08257227	102.4403845	177.2338725	72.86220988	10.89316274	15.97923192	26.3100232	15.13370466	28.95760283	16.71733349	15.77387236	23.95268558	18.64410632
c261720.graph_c1	49.73657884	37.46439315	54.64087311	4.67515677	7.693620003	3.485109964	6.369362592	9.918717726	11.75119906	8.437185482	5.077718663	2.877879314	5.138491568	4.977529354	4.976636277

c274954. graph_c0	1.121084441	0.478387759	0.821576759	3.812311592	1.082870418	5.159663752	1.913923048	1.990076732	2.159797927	5.551262141	3.988112148	3.855500792	5.195872099	3.160766204	5.470237871
c269688. graph_c2	4.212464286	3.540411946	5.959784995	8.313772052	7.986058937	8.393232694	46.69720473	21.35879167	12.12424265	29.50193891	41.05790716	28.71822081	47.83280129	27.62226903	8.880632674
c262866. graph_c0	11.08113963	8.21630019	14.31492864	0.039948138	0	0	0	0	0	0.336437764	0.042894892	0.285253464	0.707394211	0.870651821	1.209934146
c262855. graph_c0	5.445378779	3.798900776	3.827498114	9.026584658	16.83377053	11.57918215	10.97287651	6.714954217	6.124243835	15.54288806	21.20773957	13.10785838	12.80683932	33.96429434	33.66776097
c263508. graph_c0	20.95565158	25.25197797	16.57051062	0.895848418	1.310824586	2.043262568	0.734159547	1.115100018	0.89046552	4.221446623	2.725468951	1.599222636	2.291396982	1.331223831	1.608854836
c265709. graph_c2	3.71218194	1.935203451	2.494717618	0.37322659	0.360074596	0.44413565	0.331161124	0.796407011	0.76316704	0.70563056	0.515259589	0.697991863	1.951236603	1.320504358	1.490656661
c253322. graph_c1	0.143623758	0.655136377	0.247185999	0.563163132	0.351067038	1.443417454	3.318456612	0.785728103	0.551167257	1.146631065	0.837283254	1.237329146	3.477550775	20.17037696	17.15780103
c272831. graph_c1	34.24749173	23.66857906	36.91094363	0.354718549	0.871102282	0.690727548	5.708222399	4.369456676	5.641394345	3.530553249	1.973672577	1.918864715	4.225595262	13.73678065	5.446920836
c260150. graph_c1	0.082663237	0.15082656	0.085361343	5.68475604	6.971003026	5.874693764	2.168052683	0.558635823	1.784399355	5.669554678	4.578066868	8.901872657	7.505694241	3.482742699	8.277776404
c273508. graph_c0	4.287417674	2.917191191	5.286176443	5.086235977	4.994629058	9.974382396	8.260362655	17.20735304	10.00817144	11.59870256	12.07921114	12.60038268	9.64994174	9.331948905	14.41951522
c278219. graph_c3	0.147940268	0.58484923	0.458306998	6.514841199	5.122923199	1.752298043	1.293369365	6.379524046	8.019217282	0.053686011	0.143741201	0.159314524	0.158032328	0.353643971	0.155942352
c259496. graph_c1	5.102638168	6.639259073	6.737648871	5.979678883	13.29052279	14.32158065	2.350637099	1.211363635	2.046455246	8.560296034	9.021583778	4.954463103	2.859396971	2.999411523	4.408721061
c271112.	3.98691987	7.82008353	4.665992139	8.357702152	3.979386394	5.759848066	0.342332664	0.737738472	0.717192785	1.229790603	0.968439256	0.679796357	0.53236201	2.025239083	1.575964587

graph_c0															
c278112.graph_c4	8.329334032	3.213427989	12.45248547	3.224768223	3.1907189	1.294020361	0.815141322	1.840307466	1.550514962	1.127847087	0.885792037	0.49088061	0.354130836	0.928678425	1.223066109
c266863.graph_c1	14.80520095	4.008090179	4.847553773	13.50568801	21.11032259	16.84928351	48.1997413	58.68389184	56.81610147	30.20481059	67.48074639	22.8782539	111.7991026	129.4257151	22.96494709

NAC Family

GeneID	T01_FPKM	T04_FPKM	T012_FPKM	T02_FPKM	T03_FPKM	T05_FPKM	T06_FPKM	T09_FPKM	T013_FPKM	T07_FPKM	T10_FPKM	T14_FPKM	T08_FPKM	T11_FPKM	T15_FPKM
c278698.graph_c0	3.388125364	2.166568287	4.316169168	0.429786516	0.696597598	2.659495053	33.60508488	5.135743323	9.569370735	14.09921696	15.44451519	19.19787056	30.17172108	79.39246494	103.6370345
c268480.graph_c0	10.59455982	16.37302581	10.64144644	1.047725843	2.15806957	5.080638876	48.20953937	6.176073284	14.9132827	15.91436089	12.37511884	12.62483835	41.34212309	72.91540258	45.21970399
c268037.graph_c0	0.0834454	0.025375615	0.057446024	10.52066664	1.393794593	5.930280361	4.872166761	1.315817202	4.082909076	9.871763377	6.296974511	11.98147006	5.110567492	6.208570513	23.13320612
c259996.graph_c0	0.023601192	0.172250049	0	0	0	0.050826807	0.486357945	0	0.169821369	1.07914356	2.247268096	0.71164097	1.966473417	2.602256958	0.721455616
c203597.graph_c0	5.445628222	2.458402089	28.40672503	0.05079981	0	0	0.052586667	0	0	0	0	0	0	0.050325417	0.177531404
c263188.graph_c0	3.361153681	2.373099573	3.712312044	29.17103746	35.47100978	17.49823367	3.230516252	4.430048868	7.613029216	12.63214286	9.42808486	7.333569657	8.304848276	16.47981382	15.49659183
c273598.graph_c1	22.06726022	35.28940936	27.11662929	166.6093322	35.20569523	109.0600596	122.648889	23.41483793	80.16927806	285.5434011	301.0973979	382.9640982	202.7300722	131.0928816	478.4475147
c275586.graph_c0	36.20319562	39.08799746	50.4200581	64.54752263	56.18714486	70.56767468	88.09278359	69.13159507	83.04417701	86.6297575	101.8895359	115.6325246	134.452853	139.0823819	116.6979271

c269036.graph_c2	8.830849714	20.56808642	6.252100855	160.8444018	31.39739097	166.4421917	82.65568919	45.14649856	139.7924032	173.7416636	140.2732543	195.2391123	195.5615863	106.1782638	269.4884186
c261856.graph_c2	2.809866614	6.292050623	5.275599859	22.88305722	55.57081872	37.90278545	17.46687742	51.14699299	19.26250205	37.09751684	43.78107987	52.76070531	65.05172336	67.00056528	33.38815213
c276126.graph_c1	15.86737242	17.67832374	17.2794612	14.48495852	12.78546767	21.34458042	92.22469475	54.72273499	164.9951402	64.38379252	65.5336213	36.87131426	146.8982576	258.2524796	111.1588866
c263014.graph_c0	1.826735624	1.274399387	2.700083628	2.949393694	1.882381958	2.931217231	13.31973002	7.365520803	8.350431708	8.539759282	10.92773211	9.604479634	13.31509601	41.67650968	25.56064862
c274411.graph_c0	18.89071758	21.62645832	26.38244141	13.5706742	36.65889425	71.04548888	236.1836131	202.8817609	92.89474048	192.4652773	230.5956009	114.119906	283.0036127	398.5479091	170.883383
c266011.graph_c0	0.194448707	0.025342091	0.057370132	13.82469444	10.79612521	6.341204661	7.49374481	17.37802267	16.94967078	0.544346601	0.188929567	0.329055137	0.029673348	0	0.058561837
c265087.graph_c0	0	0	0	0.159431461	0.16150398	0.047430433	0.165039381	0.595353034	0.316946965	0.911124644	2.696271491	0.948696213	3.434872312	19.70334094	6.964615407
c265471.graph_c0	0.272897581	0.18672238	0.598835384	72.41416711	5.461535599	23.25103553	20.96104507	24.93009386	103.7283783	6.870327181	5.236747761	10.57964652	5.757398927	5.840556001	22.00588306
c265700.graph_c0	0	0	0	0.035973046	0.340112966	0.214037697	0.372383791	0.498945445	0.17163309	0.779040206	1.390556839	0.470926393	2.802817671	17.35527351	7.249618576
c277460.graph_c0	0.032199918	0	0.099752744	0.976079978	0.806756363	1.057507592	0.422262187	4.010170237	0.486555577	0.350550633	1.595584504	2.184561545	0.395559793	0.533995526	0.967335895
c268319.graph_c0	11.64783303	9.062725682	17.56680155	27.56996013	93.28846064	30.65493543	143.7093786	65.93859271	111.5355013	79.02076482	251.630975	112.9244754	143.308139	419.7874125	262.5187962
c272369.graph_c1	9.471279056	6.883499339	10.94914845	33.11322054	26.23310644	44.79228021	39.29969174	49.59124564	41.40445104	32.44643137	32.37256261	38.27474053	35.63354839	27.14383603	54.71085681
c261856.	5.31866193	15.68498673	8.302255995	57.86424707	140.8911185	80.17875573	38.3496423	102.3370956	41.11821967	74.33083307	100.3495145	118.8140379	145.2082058	155.0073272	65.58123561

graph_c1															
c269036.graph_c1	6.73247171	16.19255104	7.268227719	123.0400741	22.31605415	86.22419509	90.77235441	51.80058693	135.9345896	173.3522555	126.5657999	177.62725	189.1645967	108.449216	276.9836536
c265893.graph_c0	29.28979247	47.66963675	23.96906524	846.919766	235.3758262	665.3385748	170.2743377	146.5672668	270.9614126	1041.045337	1116.752202	1425.846164	461.3060509	363.1220733	1348.979819
c276232.graph_c2	14.63534665	6.532824243	14.24943821	5.391823489	5.493855183	8.554949705	53.6605265	45.06244748	31.8932833	10.69790713	14.27072941	15.47913353	29.42490936	54.67980589	39.39387507
c275381.graph_c0	11.30102889	11.00625891	14.39991555	29.75129171	16.65613181	34.84239153	34.93088883	19.50191984	26.44446825	38.36859533	29.23247049	40.27895564	49.97828151	26.46936989	37.58164205
c265374.graph_c0	8.042363399	7.215234016	21.8476506	0.362354559	0.856484917	0.359331707	0.125033398	0.128867967	0.192094546	0.581276312	0.583625186	0.359364871	0.178236314	0.688027208	0.562813249
c263478.graph_c0	126.2034524	67.90821847	85.15220856	24.28461821	33.3716759	30.24059838	47.43173061	47.11895294	50.3934752	106.5650286	68.27435845	36.57730308	70.48539982	39.87525333	56.74295486
c273192.graph_c0	26.5225772	19.06572636	29.86215932	23.00012761	28.17905604	32.8768187	60.68467322	60.59377991	44.06116721	44.74501722	52.28242848	44.9193317	62.27203718	79.36792355	64.69016222
c263436.graph_c0	0.956778179	1.773005428	1.636386872	0	0.073084462	0.064390259	0.224052949	0.173193213	0	0.911413185	0.81341876	0.708358216	0.926229946	1.447325352	1.166113135
c268469.graph_c1	3.081537604	1.437582371	3.001316013	0.411921049	0.128392551	0.226237681	0.164003908	1.048008556	0.302360073	0.228734694	0.544376854	0.603356163	1.159594187	0.565025998	1.070435434

NF-YA Family

GeneID	T01_FPKM	T04_FPKM	T012_FPKM	T02_FPKM	T03_FPKM	T05_FPKM	T06_FPKM	T09_FPKM	T013_FPKM	T07_FPKM	T10_FPKM	T14_FPKM	T08_FPKM	T11_FPKM	T15_FPKM
c275151.graph_c0	14.59398114	5.01613781	14.55065848	6.864236856	7.380585282	8.128229033	2.845764886	3.418880762	3.165063429	8.66434797	8.14928304	7.747619688	5.355793638	4.644809862	7.426454044

c271026. graph_c1	52.51134806	39.62670399	74.48970952	8.141572994	4.976884661	12.19400153	17.58244461	14.04054953	18.0270813	15.19962929	10.62621212	11.73572147	15.41121855	19.08613422	18.35517475
c276805. graph_c0	35.53356759	30.20454188	39.33566445	1.952951527	1.74039427	3.31827642	4.335075096	6.691296629	5.763612758	6.080000436	5.837038134	4.684353157	7.78403424	8.486812109	8.45506295
c271154. graph_c1	19.64679384	25.04429942	25.90236595	4.360587479	2.667982351	4.701192825	6.365853595	6.541201776	9.424510988	14.39376253	10.0648236	9.358898449	13.85936712	12.79344993	11.78747602

NF-YB Family

GeneID	T01_FPKM	T04_FPKM	T012_FPKM	T02_FPKM	T03_FPKM	T05_FPKM	T06_FPKM	T09_FPKM	T013_FPKM	T07_FPKM	T10_FPKM	T14_FPKM	T08_FPKM	T11_FPKM	T15_FPKM
c260025. graph_c1	10.29689645	13.15133395	14.19647113	66.43015188	51.22280618	44.05050043	34.87868702	32.18694621	48.45943915	24.60121987	29.68942802	31.16785619	17.42054644	23.20057218	21.88371893
c268834. graph_c0	9.03159356	2.263882716	4.320528589	34.46512133	21.15948396	52.10531201	67.00326526	23.45581211	11.1701941	51.83224072	17.6906698	8.607336611	54.18741764	7.190779466	2.646165358
c267791. graph_c0	26.8264242	8.013910865	39.00011514	0.047596876	0	0	0.049271071	0.203128536	0.075697407	0.057264913	0.255539173	0.11329	1.573295061	3.960801006	3.714882602
c260025. graph_c0	5.571644999	8.495847023	9.53437479	37.59600798	30.35105315	31.88285673	27.58585916	22.43812606	40.89234397	15.51080794	22.89138016	19.88576729	15.81458784	17.9803054	17.11564397

NF-YC Family

GeneID	T01_FPKM	T04_FPKM	T012_FPKM	T02_FPKM	T03_FPKM	T05_FPKM	T06_FPKM	T09_FPKM	T013_FPKM	T07_FPKM	T10_FPKM	T14_FPKM	T08_FPKM	T11_FPKM	T15_FPKM
c265977. graph_c0	1.319002774	1.403873329	1.22584921	0	0.107470241	0	0.082367113	0.212232942	0.189816561	0.861574727	1.110689514	0.378776836	0.28179627	0.630602318	0.880553473
c263793. graph_c0	20.45252002	9.398680478	9.728419496	46.06089862	43.08474485	79.48554706	63.28060435	14.5523045	28.20849194	78.54317787	33.33654394	22.41771298	82.9434705	27.95054297	14.57541195

RAV Family

GeneID	T01_FPKM	T04_FPKM	T012_FPKM	T02_FPKM	T03_FPKM	T05_FPKM	T06_FPKM	T09_FPKM	T013_FPKM	T07_FPKM	T10_FPKM	T14_FPKM	T08_FPKM	T11_FPKM	T15_FPKM
c259122.graph_c0	0.385361982	0.301340499	1.250668873	2.889246884	7.670248818	2.904663301	5.569214893	6.005754916	2.772855662	13.96441478	17.2235063	12.3904216	7.762547928	10.11662878	8.936536373
c269493.graph_c0	4.120761728	1.392352388	7.801314233	2.175104517	2.145089186	1.561227653	0.964976874	1.510274673	0.988358826	16.49074388	13.08649437	11.01177966	11.57528317	16.55427767	11.78417132
c271309.graph_c0	44.8051811	71.66323256	59.31744929	74.94576192	170.9050468	200.6751384	513.042951	292.8446516	199.1711477	372.8314093	439.5204293	404.4416044	722.335256	568.3173101	341.4240847

S1Fa-like Family

GeneID	T01_FPKM	T04_FPKM	T012_FPKM	T02_FPKM	T03_FPKM	T05_FPKM	T06_FPKM	T09_FPKM	T013_FPKM	T07_FPKM	T10_FPKM	T14_FPKM	T08_FPKM	T11_FPKM	T15_FPKM
c265404.graph_c0	324.2547066	178.9215293	319.1605011	74.19820691	70.54630635	63.02277988	82.64770422	137.7221247	118.5315386	156.1818692	109.5303589	72.26963121	127.0796939	182.7113163	172.0178314

SBP Family

GeneID	T01_FPKM	T04_FPKM	T012_FPKM	T02_FPKM	T03_FPKM	T05_FPKM	T06_FPKM	T09_FPKM	T013_FPKM	T07_FPKM	T10_FPKM	T14_FPKM	T08_FPKM	T11_FPKM	T15_FPKM
c266547.graph_c0	10.00953039	7.538918089	15.34410648	3.229740702	2.750146086	1.838127207	2.36214605	6.367387091	7.70480186	2.74538556	2.450203876	1.086266322	2.030717974	2.260572703	3.394296436
c273345.graph_c1	49.89071687	12.84213063	37.22388136	72.30873219	62.92378622	45.72404219	24.11295694	19.0335318	30.94614892	96.90247567	50.81714263	50.12521045	55.70336124	17.49878644	43.44868457
c277167.graph_c1	3.925504259	4.928989567	6.233003563	6.454833464	5.880999326	17.81670798	25.18554365	18.25613561	17.43342802	18.19716288	12.877662	19.31836668	21.59770247	16.79989628	20.55569472

SRS Family

GeneID	T01_FPKM	T04_FPKM	T012_FPKM	T02_FPKM	T03_FPKM	T05_FPKM	T06_FPKM	T09_FPKM	T013_FPKM	T07_FPKM	T10_FPKM	T14_FPKM	T08_FPKM	T11_FPKM	T15_FPKM
c269435.graph_c0	6.775736189	2.912851961	6.267002573	0.132327094	0.178729696	0.131223189	0.79905952	0.870626238	0.561203108	2.653430303	1.207749144	1.049882398	2.030793813	2.16300744	2.517766482

TALE Family

GeneID	T01_FPKM	T04_FPKM	T012_FPKM	T02_FPKM	T03_FPKM	T05_FPKM	T06_FPKM	T09_FPKM	T013_FPKM	T07_FPKM	T10_FPKM	T14_FPKM	T08_FPKM	T11_FPKM	T15_FPKM
c269000.graph_c0	63.70767631	49.29233099	53.80523916	12.83688393	11.66592121	9.853804647	16.77457295	8.665646471	16.25686762	33.74877558	25.35536698	23.95308493	24.13448352	22.09776202	23.07684617
c271722.graph_c0	16.33064129	4.900778572	13.40217915	2.916531954	5.357394017	7.357760676	2.495805431	2.738305591	2.535665089	4.912526812	2.881134964	1.943738799	3.167584942	4.160586199	3.170993575
c269200.graph_c2	25.72817006	21.14391724	28.01709063	5.002449443	3.274370707	5.311783955	7.090435628	7.923720846	13.89209588	11.43527118	10.86686173	12.77693534	11.72013908	11.47443063	11.56514037
c264639.graph_c0	23.05606093	17.61275314	24.90819889	21.91004603	17.65404122	26.32198161	73.24615153	64.36304692	61.62910957	52.41856162	65.5405663	55.73994586	58.0608516	70.80206446	61.09951692
c273431.graph_c0	9.614784309	5.626049277	9.928608344	27.39397243	39.15844436	33.29276221	39.88435683	22.21810004	15.65184518	22.52152424	23.09596686	23.30406576	37.75896217	27.11302638	14.0351389
c277052.graph_c2	4.467859339	5.077930455	6.675673047	11.04437522	16.53406045	16.63665461	18.61053773	16.02457208	15.91250595	15.0811839	14.23571981	26.18028611	17.11752714	18.03402339	14.60216069
c273485.graph_c1	15.9978631	15.52240889	23.62504168	18.5856791	17.7709663	24.16055227	91.53011604	77.84536409	67.8477577	50.81008361	62.27394455	58.28997435	61.29661214	73.10157369	66.6310248
c276525.graph_c0	8.843116558	8.508002219	9.262956916	2.885729798	1.785779051	2.613114065	3.915537619	3.949756444	6.125327427	4.426312581	2.962799215	3.4616695	2.945203988	3.473818111	4.017862256

TCP Family

GeneID	T01_FPKM	T04_FPKM	T012_FPKM	T02_FPKM	T03_FPKM	T05_FPKM	T06_FPKM	T09_FPKM	T013_FPKM	T07_FPKM	T10_FPKM	T14_FPKM	T08_FPKM	T11_FPKM	T15_FPKM
c260062. graph_c1	18.50821426	16.71440128	14.51763916	49.53204162	42.40734366	73.47694293	93.9678706	30.14144924	87.65340588	36.79795992	34.14931862	32.73548923	47.96913236	15.88725211	16.27747551
c271433. graph_c0	10.77371079	6.781237511	8.325960755	17.9624799	7.673841473	23.69158586	32.45401776	29.85280859	11.85479095	42.21300662	39.08474711	45.61710397	49.49098476	31.85806767	12.70227474
c274498. graph_c0	2.287622945	1.212709011	0.893839594	36.7285077	27.88321579	31.22370629	17.95328011	25.72635894	17.61717636	31.73660078	27.72357896	25.8668005	21.03544446	19.89703181	9.091480399
c275253. graph_c0	2.776011847	2.532542488	4.281787052	10.96981117	24.54393729	12.46747578	2.287591224	2.849652116	5.233870413	6.694655978	8.330650597	6.887071355	5.067427323	5.890429418	5.648612035
c268333. graph_c0	0.117662337	0.026835699	0.364508415	1.996294342	3.41534719	3.357470732	6.282199442	11.84218577	3.047883537	0.448333827	0.571613326	0.411803281	0.188533386	0.052737386	0.124026689
c270100. graph_c0	22.17380122	14.96621072	20.90298831	4.823843136	6.074745407	5.213432299	11.62742187	20.08938742	19.53151833	28.51372412	15.98942058	21.2161904	30.56402834	26.61812325	22.47734516

Trihelix Family

GeneID	T01_FPKM	T04_FPKM	T012_FPKM	T02_FPKM	T03_FPKM	T05_FPKM	T06_FPKM	T09_FPKM	T013_FPKM	T07_FPKM	T10_FPKM	T14_FPKM	T08_FPKM	T11_FPKM	T15_FPKM
c275312. graph_c0	97.89420299	45.62761775	106.0843367	26.34701105	34.67800634	29.39524762	10.36461866	27.85076306	18.63065778	52.21165544	36.56875373	30.04481784	29.38087189	27.68799961	32.95792503
c269974. graph_c2	4.01029641	3.530203427	8.427687826	0.127326103	0.429937569	0.454550101	0.263609467	0	0.404995317	1.685079094	0.751950361	0.37882671	0.601244534	0.693753886	1.112424528
c262613. graph_c0	10.56532709	8.100899998	13.30357715	1.552517552	3.531675862	4.829375662	1.607126562	2.528211588	2.77232535	3.014807934	4.00674097	3.306320674	2.668784206	3.50776353	5.489080661
c263169.	25.99716268	14.39966096	24.32891856	3.500600187	6.666679322	5.790290722	2.065527244	4.29509579	3.39605649	5.980548479	5.450289242	5.707503897	5.372291475	5.513976731	8.278094809

graph_c0

WOX Family

GeneID	T01_FPKM	T04_FPKM	T012_FPKM	T02_FPKM	T03_FPKM	T05_FPKM	T06_FPKM	T09_FPKM	T013_FPKM	T07_FPKM	T10_FPKM	T14_FPKM	T08_FPKM	T11_FPKM	T15_FPKM
c267038.graph_c0	4.142116679	3.483612104	3.375068299	0.029281828	0	0.034845063	0	0	0	0	0.157208938	0.104544836	0.518517184	0.174050284	0.238774574

WRKY Family

GeneID	T01_FPKM	T04_FPKM	T012_FPKM	T02_FPKM	T03_FPKM	T05_FPKM	T06_FPKM	T09_FPKM	T013_FPKM	T07_FPKM	T10_FPKM	T14_FPKM	T08_FPKM	T11_FPKM	T15_FPKM
c268585.graph_c1	20.35274937	16.09757233	19.76043248	75.84106148	61.08974384	95.03281329	152.7144446	92.84099739	97.06240584	183.2871902	296.123493	297.3448525	244.3848861	317.3304921	250.2923371
c268317.graph_c0	4.968075716	1.527205563	4.238018071	17.73751749	28.34636146	10.87353798	9.68653719	13.91970319	5.129000084	17.48973949	13.69417128	7.443536444	8.710375192	17.69289442	7.086754848
c277066.graph_c0	20.79386608	13.31821573	20.65351553	41.54978758	41.23841933	40.18751664	53.43278074	40.50164558	45.00981131	49.31272447	62.38141256	47.94783208	54.11115031	64.91338844	46.30022977
c268349.graph_c0	1.422657809	0.96294619	0.900411831	0.290685006	0.56088333	3.01437642	14.70158833	2.569715752	5.085315185	16.73707481	18.90599264	19.91648875	23.97215923	49.24294687	37.44185856
c263579.graph_c0	0	0	0.048905196	4.456822528	1.099748918	3.263736084	1.463927272	0.59438502	7.974076948	0.360911068	0.184060683	0.408004663	0.455311075	0.806623546	3.694158861
c272673.graph_c0	1.338383202	0.898792159	0.902183068	104.1849565	70.586721	146.3368341	128.1693529	64.30031844	74.76841683	198.1192451	222.8010385	210.8077457	213.7973068	132.0893992	189.1224012
c274043.graph_c1	0.586113003	0.384989848	0.919970238	9.100902737	20.17194526	9.517252435	24.26626965	28.20185823	30.97222611	6.559495875	8.405481247	7.447874237	4.858504554	5.695367659	4.769538253
c276796.	0	1.463751011	0	0.120986349	0	0.119977051	0.062620991	0.365735017	0.545175838	0	0.194866267	0.167983373	1.928163431	1.71794345	1.691256437

graph_c0

c275502.graph_c0	7.693067121	2.477064273	7.330830404	17.50542069	19.70331258	19.21714501	65.19910355	31.34662876	29.54987378	24.50979491	21.59971561	25.61507219	30.33359379	17.51942269	16.06930383
c270738.graph_c0	5.30697458	2.261872073	4.274127232	194.9214981	47.1053042	191.9090861	123.5272212	24.01509256	52.81183054	234.6261432	247.6438691	302.1445898	235.296379	112.3194144	306.0300851
c277660.graph_c0	91.85542736	73.14013113	96.19414554	308.8439158	181.8993155	225.8680478	198.8341329	173.3204863	218.2934661	287.5005389	412.4928372	336.5809756	347.0618059	316.3558463	381.6697777
c265196.graph_c0	8.763967344	4.42659679	4.855161447	89.07060149	21.05445875	237.5427263	73.6712868	19.46381438	62.51945821	108.5551794	73.89598634	202.4058916	159.8339913	77.61779034	109.5873809
c269674.graph_c1	0.033712635	0.492094044	0.696260136	0.061011054	2.389760998	0.617121267	1.199984652	1.757538293	0.630702345	0.770740159	1.736054843	1.125442642	2.196757273	8.582665121	4.975061075
c271557.graph_c0	14.17069712	6.72754868	14.1557951	13.8476017	15.68041303	18.69388508	35.6418276	44.03278565	32.00325891	20.51088227	28.68246734	23.59978514	33.5837498	59.18342167	40.59601623
c271167.graph_c0	27.08057341	20.93418338	34.0316806	176.9683689	102.7066649	122.8221135	241.2757101	256.7864234	297.8077607	145.3462663	204.9169486	212.2792827	215.7839755	193.1235376	212.0426053
c278738.graph_c1	4.220192487	4.345391229	6.626105263	10.00460615	17.70549303	14.82856889	14.77190143	27.06654459	14.98776905	10.15602921	25.36977916	16.71690082	15.71237898	22.34439753	11.96662442
c269937.graph_c0	9.472766353	8.294141149	10.02423248	0.928812776	0.752709456	1.452650124	5.57660308	5.549434239	5.528839602	2.203024799	1.652711946	2.810821589	2.976168013	13.59176861	6.368236783
c269890.graph_c0	8.209709301	14.97935798	10.15137555	48.78082287	44.26890165	56.98632906	35.61169919	21.12427407	31.03213618	64.75007751	97.84281796	103.2830252	62.47977871	69.0767295	113.3904143
c274390.graph_c1	1.842750697	0.72519452	1.156663245	14.74547553	3.841929884	11.67203425	7.513612194	3.662722717	8.839622555	7.198524545	5.195806788	7.198418753	8.066817476	2.170112941	16.41539576
c274367.graph_c0	7.146475491	5.250528361	3.75874481	0.879461131	2.631921054	1.518522569	0.78543945	0.662340752	0.795329389	2.634877044	1.129497686	1.087690865	1.078936901	1.059164598	1.285636797

c274975. graph_c0	20.21519031	12.20123625	18.62617883	8.953278179	9.286643414	8.691670084	8.270431875	12.08910001	14.85233871	10.07610566	5.887823714	4.690366263	7.611074895	6.068720312	7.161096724
c268585. graph_c0	3.977870934	6.204407134	6.227814743	20.60977186	19.21139302	33.5065434	60.03767697	30.16520593	32.50050085	62.02516898	95.50203661	105.6417178	87.65749976	96.62275271	81.69653027
c276418. graph_c1	3.411127833	1.601741956	1.968436966	3.722106261	15.44981904	9.344672296	15.13018879	9.782701151	11.83917179	16.21965169	21.68922144	13.18098541	14.78964333	58.45794718	38.65308294
c272385. graph_c0	20.88823954	8.024570078	18.18536783	1.189698519	2.715862381	1.854404953	1.474385525	2.127443504	1.518993751	6.652772484	5.127816424	3.888627307	6.468447066	4.382357391	5.992510372
c278042. graph_c6	0.628618162	3.297540922	2.92111242	5.688172484	22.47225056	9.476410177	14.27900877	14.26175992	9.951029217	15.56912901	32.52380929	26.73948205	28.70665432	22.399338	16.73115745
c260649. graph_c0	2.637773411	1.48761017	3.417218279	0.694353473	0.058614973	0.309852452	0.494159188	0.463012943	0.621163171	1.253089325	0.698973845	0.774702623	1.741860016	0.687869266	0.657197388
c269839. graph_c0	2.081265001	0.271246889	1.565843488	24.53635307	7.812707678	37.8741571	25.76145968	7.54924334	10.09786909	43.43880297	28.59956353	43.96113019	47.4821166	28.03859238	26.60815188
c265802. graph_c0	1.046260318	1.908996686	1.337650451	25.20101889	8.768326747	10.83679154	23.47403985	14.71838709	41.2982201	11.44455489	15.58731099	20.28062009	21.34147186	7.637086253	18.80100934
c273095. graph_c2	3.131348137	4.712357456	6.743481217	6.345014469	12.03724459	11.75802952	18.05000731	14.39192949	16.02238692	12.79104435	17.1886521	19.42559633	19.41220206	22.09613165	25.22090642
c255446. graph_c0	1.994736742	0.379122688	1.416143042	9.588934742	2.234759541	11.09749406	41.33978939	31.81528895	45.57091125	1.673948397	0.565284731	1.118802245	1.242973637	0.707797585	0.788486994
c276248. graph_c0	18.67123004	15.11977886	22.22691134	26.69033819	23.64000794	39.89353423	63.80645331	45.59075171	39.54964692	44.23932948	40.48154104	46.04220069	56.1147179	44.4383342	38.85369834
c272496. graph_c0	18.52692887	14.57252711	22.24425223	54.63646636	26.74944317	35.70239486	68.06775042	100.9235084	86.36812399	22.57334235	17.70260596	20.24080511	18.84738788	26.36610711	22.82773907
c270278.	37.66585914	25.09893714	40.97345544	200.7918786	197.9726947	164.8670058	48.04884423	38.26230914	44.36419064	47.80259788	64.77205967	53.71293885	50.17810481	39.8120582	48.57435121

graph_c1															
c275362.graph_c0	7.700588824	4.421199322	7.973139178	30.9566172	100.363973	41.21615413	87.44228372	138.4967503	76.21358678	8.428106389	10.75415525	8.248414356	7.91880056	23.96706077	6.840020903
c274390.graph_c0	10.39932822	6.639031502	10.15891293	27.70167201	13.94510474	26.28952437	13.40179961	10.90713313	19.32919957	15.50277858	10.40968392	14.10138662	16.6271213	7.00671841	27.36905534
c275102.graph_c2	1.933562732	2.204975177	1.889709049	118.2244223	26.62768269	80.67869489	56.88993957	14.03365095	97.53914207	77.13359767	70.21571743	141.1449728	77.82376391	47.0152922	210.0749368
c265200.graph_c5	35.1189072	23.54605615	28.68662134	1.247944662	9.571551971	7.637352682	20.39262467	10.93698545	10.91592583	16.83748042	22.01426371	14.21527378	19.25715319	63.49236081	45.53325907
c278042.graph_c1	3.849731204	1.33793965	4.070037454	13.60225141	30.41478317	21.22015087	28.71948397	24.42353562	25.6560099	28.48939466	60.47080647	43.82612345	48.17322882	33.56481855	31.40090444
c268377.graph_c1	1.49994957	0.443804401	0.837247313	1.907498083	21.25523935	3.928677084	3.721349009	5.596664921	1.10845209	17.91830642	16.70073788	13.79529484	33.60440353	18.86050936	19.6139606
c270595.graph_c0	6.322362915	3.809768498	7.058730228	84.35706149	61.44565513	91.08918595	92.00080336	69.43876533	68.02020924	75.15382476	122.4048877	99.84054984	82.14041069	93.12664381	155.8619283
c263936.graph_c0	6.308052885	2.761144692	6.184958899	44.19347205	59.8853021	51.80074111	188.3633991	241.8140382	196.3661393	35.51616656	85.83706795	39.96916095	67.17950818	39.58261586	35.83209775

Whirly Family

GeneID	T01_FPKM	T04_FPKM	T012_FPKM	T02_FPKM	T03_FPKM	T05_FPKM	T06_FPKM	T09_FPKM	T013_FPKM	T07_FPKM	T10_FPKM	T14_FPKM	T08_FPKM	T11_FPKM	T15_FPKM
c265476.graph_c0	14.40671052	9.737055603	13.6616423	2.643959546	2.331138669	3.189981962	6.120145482	5.837690686	5.60655519	9.940654664	6.427157617	3.190276373	9.883957287	4.729235652	6.887157773

YABBY Family

GeneID	T01_FPKM	T04_FPKM	T012_FPKM	T02_FPKM	T03_FPKM	T05_FPKM	T06_FPKM	T09_FPKM	T013_FPKM	T07_FPKM	T10_FPKM	T14_FPKM	T08_FPKM	T11_FPKM	T15_FPKM
c233570.graph_c0	8.004695515	3.725839737	3.542557146	0	0.499140166	0.175904774	0.459059928	0.709707811	0.352637414	2.134155081	1.587243679	1.055526054	1.308788699	1.537620504	2.755157298
c265293.graph_c0	13.26046678	4.969212093	12.18042909	0.203948817	0.367288954	0.283146395	0.387058122	1.015454509	1.783965446	4.457656468	3.248396115	2.265380219	2.728679671	1.582679912	2.177832503

ZF-HD Family

GeneID	T01_FPKM	T04_FPKM	T012_FPKM	T02_FPKM	T03_FPKM	T05_FPKM	T06_FPKM	T09_FPKM	T013_FPKM	T07_FPKM	T10_FPKM	T14_FPKM	T08_FPKM	T11_FPKM	T15_FPKM
c278210.graph_c0	37.45038747	22.81568267	37.43157178	6.220378145	9.020179336	7.629244298	11.10066669	13.0493752	17.53999981	11.68495726	9.117341757	7.44828298	9.505727082	11.00138756	10.2691148
c270337.graph_c0	23.50229833	17.01761481	22.62958236	4.866389937	6.728131604	8.048055299	10.29328759	13.40940569	20.07988228	11.29482296	13.0222472	8.0715992	11.62545639	9.33820889	9.463044706
c249723.graph_c0	13.3890218	5.645468671	6.080357549	0.678729087	0.962573057	0.888448388	1.721377436	3.113848069	2.914491441	7.186034408	3.716855469	2.705978901	2.804388702	1.680976968	2.055709087
c267285.graph_c1	5.74109892	2.345183915	4.335759063	0.542755334	0.942532883	0.276802732	0.401318911	0.827253402	1.418098702	2.285505673	1.207210763	1.199589209	0.594967332	0.460874419	0.812906163
c265268.graph_c0	0.465062666	0.718002952	1.108251205	17.35076976	22.77926051	23.6518647	10.85706724	24.59044866	19.97506686	12.11224105	25.44333726	14.40816579	24.15153502	30.65752863	14.2163188
c258973.graph_c0	27.73290877	8.158527324	39.84431809	14.82039498	27.63138365	8.601661742	3.341289356	4.80186421	4.627268614	25.15997289	17.67094541	11.5781478	13.2023423	15.13236292	22.24248544

bHLH Family

GeneID	T01_FPKM	T04_FPKM	T012_FPKM	T02_FPKM	T03_FPKM	T05_FPKM	T06_FPKM	T09_FPKM	T013_FPKM	T07_FPKM	T10_FPKM	T14_FPKM	T08_FPKM	T11_FPKM	T15_FPKM
c268883.	0.025746974	0.093955371	0.026587348	7.175674732	8.905255911	5.184378393	11.91386122	19.21427595	21.49030506	0.056059882	0.225145657	0	0	0.023080081	0

graph_c0															
c275105. graph_c0	4.609163278	5.513502274	4.660858162	0.346115365	0.70122934	0.247124149	0.286631837	0.276958466	0.385319935	1.728140845	1.709573382	0.597271812	0.531175377	0.685766345	1.128939729
c270401. graph_c1	48.64225592	18.79909719	51.91873274	149.7182487	162.3579699	138.012263	101.3242429	124.2353677	114.5159601	97.41542939	123.9879697	124.640148	143.9520226	111.8788498	117.5690685
c271658. graph_c1	27.52832333	31.08531021	23.22010413	8.558224236	7.846545916	7.250337712	10.59003708	7.135447635	6.354748834	18.07019117	10.52838733	13.08553797	12.84640608	8.759041933	6.932467777
c278825. graph_c0	4.098550205	0.714825643	2.365123245	75.04566552	128.5804596	115.8479779	41.24211796	52.54452032	31.74613245	100.525468	76.30634148	66.49703482	80.31958154	21.39575708	32.56063723
c265634. graph_c0	6.842896701	4.063164181	7.431742589	44.17085747	56.70410442	39.85883633	25.72178319	42.31449317	26.78364933	32.39956336	31.46655575	31.03241207	20.47976138	26.81026336	19.58710394
c275639. graph_c0	35.06153483	13.61673127	40.21496756	52.65841744	72.27941697	65.54375985	76.65030264	65.81478094	54.77004029	42.68166645	31.0564852	36.76074882	35.97066994	23.42360919	29.40950243
c268390. graph_c0	48.73753874	44.51422143	33.49426425	10.28264	29.28568721	17.88604719	5.072481563	6.717903957	8.196872972	10.84396532	20.55559206	10.21290862	14.17700368	11.26815766	15.43874369
c276878. graph_c0	4.578358134	2.253141657	5.7262855	5.924331607	22.14019178	8.191421676	10.36669234	32.87489402	15.54122819	8.954016787	10.93425523	8.505813887	24.9636265	22.8703311	15.55863546
c266645. graph_c0	14.61263452	5.138508963	13.33370785	0.937597137	1.039081433	0.57216952	1.244329021	1.46203922	1.758782178	3.586604671	1.936074057	1.659444748	2.866465656	2.500726853	1.428281096
c264579. graph_c0	28.51448955	12.43634259	42.31670569	3.244096646	7.234884763	2.82799697	2.69437465	2.294049002	3.17960932	12.75298391	6.44023172	4.175047483	12.95871758	8.781900012	11.20539988
c269729. graph_c1	3.55729632	4.002542363	3.183618132	34.35626538	39.44760884	37.94691582	64.17645431	76.63070678	75.62875166	33.66684096	27.8580013	36.92887246	23.40778347	45.53647162	48.07128678
c263361. graph_c0	0.460388579	0.2400059	0.373540783	14.46167536	4.300451533	11.61446582	1.786585861	0.444470433	0.946487523	16.86216773	12.39718554	6.870165616	4.601795741	0.766444973	5.511519008

c277622.graph_c1	10.85615417	17.43201292	13.55378281	15.77344062	4.53826835	21.65795263	69.34681065	15.01168882	16.36518382	44.97305708	92.60207928	88.91687781	87.1820661	119.9468102	105.7084072
c273069.graph_c2	6.995424826	6.601118338	10.72534417	18.12007116	18.43720244	13.74262164	42.16671476	36.60323599	13.06411852	15.2895442	18.4449411	17.74054418	63.03251163	66.46604058	39.51459998
c274351.graph_c0	2.3634897	2.171601915	3.20768959	10.72379797	6.602515518	9.307317513	8.270404677	30.96689162	15.0385069	2.40764785	3.64144305	4.399567802	3.083765356	1.43767397	4.324238423
c275547.graph_c0	8.226568994	1.94812196	8.784276328	4.498058085	2.053647465	4.561053253	41.67694011	22.10278327	15.51636512	12.27164394	5.918273072	7.275739845	23.85783865	38.53544468	30.07365202
c269402.graph_c0	0.649367978	1.718004477	0.60350686	317.6724265	149.8131547	269.2266753	22.60704893	11.38897005	18.03584812	324.2765923	213.6141098	284.1231765	151.1729424	21.94539209	161.1979295
c277256.graph_c0	9.655531832	10.17829691	11.66782387	39.46661732	69.22082396	61.97318573	35.27430171	61.13257185	32.17953924	62.82923192	67.86598058	60.396248	65.98727534	51.4507791	44.90891441
c267928.graph_c1	15.17422471	14.41818484	15.12730993	0.332577252	1.411773816	0.734989121	1.229555311	0.811048799	0.906730671	5.716163734	1.632501226	2.09208518	4.655285246	2.68283924	3.818874957
c267928.graph_c0	15.56653744	14.42404369	16.26373837	0.497141688	1.566768779	0.657325879	1.086437717	1.178691552	1.844843485	4.319775617	1.601439269	2.037898289	3.586526681	2.571939948	2.380845664
c269480.graph_c0	22.94733344	26.51423385	18.08450303	213.7478029	262.2802167	210.2953639	84.30331153	108.022969	77.39761968	307.0178128	310.8530179	363.1892492	283.998319	168.7019531	167.4702702
c277926.graph_c1	11.77102421	10.07350897	12.03520267	28.6646421	13.21108449	38.46831145	41.66875757	22.13640699	40.34174726	42.24037127	37.14432702	46.58497549	39.85532144	29.8556408	56.70349638
c273271.graph_c0	6.073767649	3.148799653	5.90171043	0.405606318	0.794366014	0.627467334	0.398879647	1.29825041	1.548169518	3.049965228	1.371905831	0.917152281	0.933712185	0.82372807	1.275734564
c271045.graph_c0	38.3567948	18.30872356	33.69611126	19.6498956	56.5915293	32.62095938	246.693778	203.6405194	104.1697331	72.1954526	81.69955037	59.04072455	218.9611153	276.1859219	86.63607179
c263684.	25.91159054	16.11151191	27.76634381	9.136315449	16.09964157	9.430295603	2.84747375	4.402201626	4.843422895	15.87749878	9.107007273	8.300984903	8.156860622	7.493835504	10.14706321

graph_c0															
c262179.graph_c0	0.510783186	0.716900133	1.704085434	99.72681268	48.83688636	35.28484237	16.08325955	28.29765209	33.81293086	39.82345295	92.23272499	86.65470807	37.3544876	60.12261858	82.25270022
c268417.graph_c0	0.125897885	0.602994241	0.357519688	114.6900936	98.16866579	99.94523827	91.89558358	210.5157814	197.9375879	0.548244671	0.336392264	0.135577433	0.302594124	0.451429102	0.265415396
c275935.graph_c0	57.80122891	43.85151291	59.59448634	16.4161304	15.13784538	18.23701164	23.4296583	31.94545985	28.84021585	33.5629476	25.21079205	20.38060555	32.96461153	33.39012662	33.22751273
c256199.graph_c1	5.373845526	4.74438762	4.967470597	0	0.529660111	0.139995359	0.649505554	0.753102923	0.873132031	2.689269589	1.389543997	0.840049676	0.83328878	1.476244238	0.822268546
c256199.graph_c0	9.28289666	7.616184753	8.427861235	0.112749098	0.304572728	0.134170221	0.641932444	0.902208348	0.717258372	4.951264183	2.481852536	1.006369525	2.129642779	2.345620042	2.035807081
c268497.graph_c1	3.472047234	1.45535267	6.540884897	13.755765	13.76255034	10.35706392	5.93317375	10.8260528	9.858143885	5.15906822	7.521989426	5.254800287	3.157577296	3.743303476	5.489775227
c273261.graph_c1	29.15733649	16.84345832	20.42080688	0.876207367	1.656848798	1.621942613	2.559833202	3.34466607	2.570248047	6.723361181	5.645042127	4.333303732	5.746562025	4.282256271	5.965433125
c275367.graph_c1	9.909308113	5.317773503	13.29253682	39.91028657	37.99504058	26.41395499	19.06455098	20.44645349	22.50906361	24.06138119	19.72805709	28.66570941	15.87793894	18.98500781	15.71915584

bZIP Family

GeneID	T01_FPKM	T04_FPKM	T012_FPKM	T02_FPKM	T03_FPKM	T05_FPKM	T06_FPKM	T09_FPKM	T013_FPKM	T07_FPKM	T10_FPKM	T14_FPKM	T08_FPKM	T11_FPKM	T15_FPKM
c266540.graph_c1	16.54129216	12.30806718	20.59958139	70.43772952	57.05583872	42.54619416	29.95886215	36.1565623	68.64533897	37.27236715	34.94680343	43.31945544	33.16736124	32.73475147	45.09612577
c245117.graph_c0	0.061045245	0.027845644	0	0.027618983	0.111912056	0	0.314495129	0	0	0.83072608	0.8600322	0.95321049	1.760658552	0.383054886	0.675645391

c268451.graph_c0	7.3712502	2.527919496	4.94492177	0.219088172	0.953503654	0.37658484	0.655184072	0.883055178	1.045304321	4.012427044	2.064964313	1.535449121	1.178240598	1.374600734	2.211886672
c269678.graph_c1	35.01072831	32.72507523	33.10718516	0.500505299	0.200300874	1.014719029	4.509478681	5.10266578	2.122657727	1.338154704	0.656852553	0.992751535	1.903872547	3.672824732	2.094635676
c270853.graph_c0	7.601019536	3.400326741	7.611262853	16.21902791	17.37675876	13.03232652	26.02768486	17.06184551	15.06689008	5.74462596	4.536960061	3.314755723	3.042031231	2.721725686	3.310809267
c271806.graph_c2	17.1109044	9.648822687	17.39114157	0.42670047	1.481990867	0.507768995	1.135824276	1.886060332	1.890437186	3.923638816	2.159971683	1.922445749	1.223341492	2.294742562	3.017907037
c271918.graph_c0	30.03990238	16.77810976	33.74329841	43.34048246	85.29897535	64.76489783	104.5181653	99.24875302	95.82686923	77.10763433	107.1496892	80.37051986	195.7080883	188.1692822	106.112128
c273104.graph_c1	5.759565688	1.484433843	5.360801957	0.093482582	0.631318684	0.055621636	0.266119655	0.423888983	1.152218804	2.052597166	2.057754663	1.001281851	1.903678039	0.787181567	1.579030598
c243626.graph_c0	2.40280163	2.19206457	1.702803834	0.63947686	0.230325227	0.405850999	0.661970131	0.409362999	0.339005027	0.615495639	0.274659017	0.202944228	0.553604948	0.422336744	0.446959251
c276093.graph_c1	7.337710071	10.60141793	13.20463758	23.97503641	11.55705386	28.08373851	59.52952066	40.07353674	56.26651126	37.73571479	28.219457	51.70926519	49.55490495	110.70566	53.70535392
c268088.graph_c0	1.644985998	0.567342701	1.367228561	0.054457217	0.073553507	0.086404674	0.018790907	0.2905079	0.230954661	0.41495208	0.25338831	0.410460079	0.342868725	0.377640683	0.422917862
c261129.graph_c0	25.38299595	11.72536899	27.61633742	39.8462623	39.4235029	38.16460135	50.53698059	58.51563714	38.64081702	48.21279807	46.61184709	57.3292928	65.74035701	56.90055557	48.53055893
c277233.graph_c0	61.45364106	57.58143633	55.20251804	5.730087975	5.24433325	6.961224604	10.07493631	11.66137719	12.02378803	12.96484089	19.08275467	12.49879059	20.69731701	31.7041098	26.66026382
c272001.graph_c0	7.709887483	10.24574746	12.76499635	0.488350811	0.596779863	1.826415615	5.970048501	2.679593254	3.439522271	1.930509166	2.721749506	2.380094536	2.635466831	8.708226394	4.496892987
c256460.	0.815063414	2.921201251	0.721428746	3.529587878	4.411525836	1.629917423	0.163600268	0.168617619	0.418910996	2.154955333	0.45253088	1.253898347	0.684093691	0.469696149	0.61367869

graph_c0															
c269077.graph_c0	5.341926182	5.199982987	5.80062985	13.15575896	5.721090894	11.326309	13.7732618	13.74374315	15.81093088	13.69961444	11.31700191	11.03082673	13.38341933	24.9550494	19.0694954
c242338.graph_c1	1.725786978	1.497625972	9.606041045	0	0	0	0	0	0	0	0	0	0	0.075464805	0
c262929.graph_c1	24.84222858	17.5886808	21.7692103	48.10384948	98.07639235	72.59183145	62.79254266	22.21353731	19.46705108	93.28106958	80.97911139	80.93732161	147.7423005	118.2582855	87.51968779
c268648.graph_c0	9.484739609	5.760417939	7.130708181	1.62896346	1.313543538	1.273011345	2.239959195	2.905273779	2.977349543	3.393162731	1.383634703	1.591411043	2.669274219	1.2042921	2.633973098
c268990.graph_c0	4.090241201	3.023807976	4.2722944	1.893108521	1.379033074	5.366169941	10.01867897	6.354421763	6.562809418	15.25253122	7.308773758	8.277827923	12.75624698	14.13947606	10.35746876
c275663.graph_c1	11.47785108	13.23984273	10.02438996	33.86877388	26.55800945	37.53837512	13.64865276	29.33647564	38.43859484	23.65689483	43.30405171	36.51530494	20.69795549	25.35619637	33.85581919
c275796.graph_c2	0	0.020376866	0.069194575	0.040421999	0.081894924	0.144305239	0.585813507	0.582215892	0.225003041	0.826755067	0.737863016	0.962123713	1.980339204	26.00891489	10.02972984
